# The digital divide in adoption and advanced use of electronic health records among US hospitals: rural versus urban disparities from 2008 to 2023

**DOI:** 10.1093/jamia/ocag043

**Published:** 2026-04-27

**Authors:** A Jerrod Anzalone, Yinting Liu, Elizabeth Reisher, Emily Frankel, Jed R Hansen, Carol Reynolds Geary

**Affiliations:** Department of Biostatistics, College of Public Health, University of Nebraska Medical Center, Omaha, NE 68198, United States; Department of Biostatistics, College of Public Health, University of Nebraska Medical Center, Omaha, NE 68198, United States; Department of Neurological Sciences, College of Medicine, University of Nebraska Medical Center, Omaha, NE 68198, United States; Department of Family Medicine, College of Medicine, University of Nebraska Medical Center, Omaha, NE 68198, United States; Nebraska Rural Health Association, Papillion, NE 68133, United States; College of Nursing, Omaha Division, University of Nebraska Medical Center, Omaha, NE 68198, United States; College of Nursing, Omaha Division, University of Nebraska Medical Center, Omaha, NE 68198, United States

**Keywords:** electronic health records, rural health, interoperability, patient portals, public health informatics

## Abstract

**Objective:**

Using 15 years of hospital survey data (2008-2023), we examined rural–urban trends in electronic health records (EHR) adoption and advanced capabilities for data sharing, public health reporting, and care improvements.

**Materials and Methods:**

We conducted a repeated cross-sectional study using the American Hospital Association Annual Survey Information Technology Supplement (2008-2018, 2020-2023). We constructed hospital-year measures of basic and comprehensive EHR capability, certified EHR technology (CEHRT) adoption, and advanced functions in patient engagement, interoperability, public health reporting, and social determinants of health (SDOH). We compared rural and urban adoption using descriptive analyses, Cochran-Armitage trend tests, and logistic regression with clustered standard errors, adjusted for bed size, ownership, and census division.

**Results:**

The cohort included 41 838 hospital-years (45% rural). Comprehensive capability rose from 2% in 2008 to 75% in 2020, and CEHRT was nearly universal by 2023. Rural hospitals lagged urban hospitals in adopting basic and comprehensive EHRs, with the gap peaking at 25% in 2016. By 2021-2023, most hospitals reported patient portals, interoperability, public health reporting, and SDOH capabilities. Rural hospitals remained 4%-15% behind urban peers in advanced functions, particularly interoperability, public health reporting, and SDOH data collection and use.

**Discussion:**

Persistent rural–urban gaps in advanced EHR use may limit interoperability, data-driven care, and public health reporting in rural settings.

**Conclusion:**

EHR adoption is widespread, but substantial rural–urban gaps persist in advanced EHR use. Support for advanced capabilities that enable data sharing, reporting, and data-driven improvement is needed to ensure that rural hospitals and communities benefit from digital transformation beyond adoption.

## Background and significance

Electronic health records (EHRs) have become a central feature of United States (US) clinical care. The Health Information Technology for Economic and Clinical Health (HITECH) Act of 2009 created financial incentives for certified EHR technology (CEHRT) adoption and “meaningful use” (MU), tying Centers for Medicare & Medicaid Services (CMS) payments to standards for electronic documentation, ordering, and reporting.^1^ Federal monitoring by the Office of the National Coordinator for Health Information Technology (ONC) shows that, within a decade, EHR adoption among non-federal acute care hospitals increased from well under one-third to more than 90%.^2^^,^^3^ These EHR systems are expected to support information exchange, patient engagement, public health reporting, and population health analytics, positioning hospitals to participate in value-based payment reforms. These expectations align with the learning health system (LHS),^4^ in which data from routine care are captured, analyzed, and used to improve practice and system performance.

Despite these gains, EHR adoption has not been evenly distributed across the hospital landscape. Early MU evaluations showed that small, rural, independent, and for-profit hospitals adopted basic and comprehensive EHR usage more slowly than large, urban, and system-affiliated institutions.^3^^,^^5^ Subsequent work has documented persistent gaps in comprehensive EHR functions and highlighted limited capital, constrained broadband infrastructure, and scarce informatics expertise as barriers in rural settings.^6^ Conceptual analyses have also questioned early estimates of “basic” EHR capability, suggesting that survey definitions may have obscured variation in implementation.^7^ Together, these studies suggest that national adoption statistics may mask structural inequities in hospital digital capacity.

Increasing attention has shifted from whether hospitals have an EHR to usage patterns and the EHR-enabled capabilities that support data sharing and improvement. An “advanced use divide” has been observed: hospitals differ in decision support, patient engagement, and care coordination despite similar adoption levels, with more limited use among smaller and rural organizations.^8^ National monitoring shows steady growth in interoperable exchange of patient health information, but progress varies across hospitals.^9^^,^^10^ Having CEHRT does not guarantee the availability of patient portals, interoperability, or digital public health reporting.^11^ Early MU incentives focused on capabilities like patient access and e-prescribing, rather than on structured collection of patients’ health-related social needs data.^1^ More recently, hospitals have started collecting and using structured information on patients’ social needs related to health, but adoption varies and is affected by available resources and staffing.^12^ Evidence across these domains points to a complex, multi-dimensional digital divide.

Rural hospitals occupy a critical position within this landscape. They care for older populations,^13^ have higher rates of chronic illness,^14^ and serve patients with economic and geographic barriers to care.^15^ Simultaneously, they operate with narrower financial margins and more limited clinical and technical workforces.^6^^,^^16^^,^^17^ Rural communities stand to benefit disproportionately from capabilities such as electronic information exchange,^18^ patient portals,^19^ and electronic public health reporting,^20^ given longer travel distances, fragmented referral networks, and fewer local specialty services.^21^

A growing body of work has examined rural–urban differences in digital capacity across hospitals and clinician practices. Hospital-based analyses using national surveys and federal monitoring reports have documented gains in basic and comprehensive EHR adoption,^9^^,^^10^ disparities in smaller and rural facilities,^6^ and emerging gaps in advanced functions such as interoperability, patient portals, and structured collection of social needs data.^12^ Clinician-level analyses of participants in the CMS Quality Payment Program have shown that rural physicians are less likely than urban physicians to adopt EHR systems and to achieve higher Promoting Interoperability performance.^22^ Much of this literature focused on the early MU period,^5^^,^^8^ ended before COVID-19,^3^ or examined capabilities in isolation rather than providing a longitudinal view across advanced domains.^12^

## Objective

This study analyzes US hospital survey data to examine trends in EHR adoption and identify the EHR-enabled advanced capabilities in *patient portals*, *interoperability*, *public health reporting*, and health-related *social needs screening and use*. We compare rural and urban hospitals from 2008 to 2023, examining how rural–urban differences in EHR use have changed over time and across regions. We hypothesized that overall adoption of core EHR capabilities would be high by the end of the study period, but that rural hospitals would lag in the advanced use of these capabilities.

## Methods

### Study design and data source

We conducted a retrospective, repeated cross-sectional study of hospital health EHR capabilities from 2008 to 2023 using the American Hospital Association (AHA) Annual Survey IT Supplement.^23^ The AHA-IT Supplement collects information on EHRs, interoperability, patient engagement, public health reporting, and related health IT functions, and has been widely used to monitor national EHR adoption.^2^^,^^24^ AHA-IT data were available for the survey years 2008-2018 and 2020-2023 (no survey was fielded in 2019), and we analyzed all available survey years through 2023. Hospitals may contribute repeated observations over time, with a response rate of approximately 50% across all years (Table S1). Not all AHA-IT items were fielded in every year; for example, multi-item batteries for basic and comprehensive adoption were not fielded in 2021-2023. Because AHA Annual Survey core files were not available for all study years, primary analyses are unweighted and potential nonresponse bias is noted as a limitation. For 2011-2015, we conducted a sensitivity analysis using linked AHA Annual Survey data to assess participation patterns and weighted estimates.

### Study sample and rurality

This study included non-federal, non-specialty acute care hospitals in the 50 US states and the District of Columbia. We excluded federal facilities (eg, Veterans Affairs, Department of Defense, Indian Health Service), non-acute hospitals (eg, psychiatric, rehabilitation, long-term care), and hospitals in US territories. Acute care hospitals were identified using the AHA service category and survey-reported type, including general medical-surgical, cancer, and children’s general hospitals, in line with ONC definitions.^25^ We excluded responses with missing staffed beds, which were minimal.

Rurality was defined using Rural–Urban Commuting Area (RUCA) codes derived from hospital ZIP codes. For 2008-2018, we used the 2010 ZIP code RUCA crosswalk,^26^ and for 2020-2023, we used the 2020 crosswalk.^27^ RUCA primary codes 1-3 were coded as urban and 4-10 as rural for the primary binary rurality measure. Hospital-year observations with missing or unmappable RUCA data were excluded.

### Core EHR adoption measures

We examined 4 hospital-level measures of core EHR adoption, harmonized with prior national work^2^^,^^24^: (1) basic EHR without clinical notes, (2) basic EHR with notes, (3) comprehensive EHR capability, and (4) use of CEHRT. Each composite was derived from AHA-IT items on clinical and administrative functions. Basic and comprehensive measures required functionality across multiple domains (eg, demographics, problem and medication lists, test results, clinical notes, ordering, and decision support). In this context, clinical notes refers to clinician documentation functionality within the EHR. Patient access to notes is examined separately through portal capability items. We constructed each composite only in years when all required items were present. Multi-item batteries for basic and comprehensive adoption were not fielded in 2021-2023, so these measures are available only for 2008-2018 and 2020. For core measures, responses indicating the presence or absence of capability were coded as 1 or 0. Responses such as “do not know,” “not applicable,” or missing were treated as missing. Hospitals with missing data on any required component were excluded from the numerator and denominator for that measure in that year.

Certification measures evolved with federal policy but consistently indicated whether hospitals used EHR systems meeting national standards. In 2008, certification referred to voluntary Certification Commission for Healthcare Information Technology (CCHIT) standards.^28^^,^^29^ From 2011 onward, it reflected the use of ONC-CEHRT required for participation in federal incentive and Promoting Interoperability programs.^30–32^ Because payers and hospitals use CEHRT for interoperability, quality reporting, and value-based payment participation,^33^ our CEHRT measure harmonizes these items by defining certification as CCHIT-certified systems in 2009 and ONC-certified systems thereafter.

### Advanced EHR use measures

We defined advanced EHR use measures across 4 domains using individual AHA-IT items. *Patient engagement* included whether hospitals allowed patients to view clinical notes through a portal, submit patient-generated data, exchange secure messages with clinicians, and access their health information via application programming interface (API)-enabled functionality, including support for Fast Healthcare Interoperability Resources (FHIR)-based applications. *Interoperability* included querying external organizations, having external clinical information available electronically at the point of care, using external information often or sometimes, integrating external summaries into the EHR, and integrating prescription drug benefit information. *Public health reporting* included hospital engagement in electronic case reporting, immunization registry reporting, and electronic laboratory reporting. Engagement was defined as active, piloting/implementing, or testing/validating; in secondary analyses, we also examined production reporting and EHR-based reporting when available. *Social determinants of health (SDOH)* included the collection of social needs screening, the use of SDOH information in clinical decision-making, and the use of SDOH data for analytics or population health management.

For public health reporting, the primary indicators reflect engagement: hospitals reporting that electronic submission was active, being piloted or implemented, or being tested or validated. In sensitivity analyses, we constructed alternative indicators for production submission and for whether electronic submission was sent directly from the EHR, and we constructed alternative SDOH indicators for structured electronic recording of social needs data and for screening with structured electronic recording.

Each core and advanced-use measure was coded as a binary indicator based on survey responses. For frequency items, we treated “often” and “sometimes” as indicators of capability, and grouped “rarely,” “never,” or non-substantive or missing responses as indicating the absence of that capability. Because item availability and wording varied by year, each indicator was analyzed only in the years in which the corresponding question was asked.

### Hospital characteristics and covariates

We summarized hospitals by staffed-bed count (continuous and categorical for descriptive tables), ownership (non-governmental nonprofit, investor-owned, government non-federal), and US Census division based on hospital location. These measures were included as covariates in adjusted regression models. Primary service category (general medical/surgical, cancer, children’s) was summarized but not used as a covariate because all rural hospitals were general medical-surgical. System affiliation, critical access designation, and value-based payment participation were not available across all licensed years and, therefore, could not be included.

### Statistical analysis

We summarized hospital characteristics and core adoption measures overall and by rurality, using medians and interquartile ranges (IQRs) for continuous variables and counts and percentages for categorical variables. For each core and advanced measure, we calculated annual adoption overall and by rurality, and computed annual rural–urban gaps (rural minus urban), expressed as absolute differences (percentage points [PP]). To characterize temporal trends in adoption, we used Cochran-Armitage tests for trend across survey years overall and within rural and urban strata.^34^,^35^ For core adoption, we fit logistic regression models including survey year (centered on the first year), rurality, and a year-by-rurality interaction term. We estimated models without covariate adjustment and with adjustment for log-transformed bed size (to account for right skewness in hospital size), Census division, and ownership. To account for repeated observations per hospital, we used hospital-clustered robust variance estimators.^36^ We report odds ratios with 95% confidence intervals for calendar year effects and year-by-rural interaction terms.

Secondary analyses examined geographic variation in adoption and sensitivity to alternative rural classification methods. We repeated descriptive and regression analyses stratified by US Census division and summarized rural–urban differences within each division. We then implemented a 4-category rural classification using RUCA codes (metropolitan: 1-3; micropolitan: 4-6; small town: 7-9; and rural: 10) based on commuting patterns to urban centers.^37^ In these models, we replaced the binary rural indicator with micropolitan, small-town, and rural hospitals, using metropolitan hospitals as the reference group and including year-by-category interaction terms. We summarized the public health and SDOH sensitivity indicators using the same descriptive approach as the primary advanced measures, reporting annual percentages overall, by rurality, and rural-minus-urban gaps. To assess whether rural hospitals were catching up or following parallel trajectories, we summarized annualized percentage-point changes and changes in rural–urban gaps over time for advanced measures. As a sensitivity analysis, we estimated inverse-probability weights for survey response using the AHA Annual Survey (2011-2015) and re-estimated core adoption models using weighted logistic regression with robust standard errors.

The University of Nebraska Medical Center Institutional Review Board determined that this analysis did not constitute human subjects research. All tests were 2-sided with statistical significance defined as *P* < .05. Analyses were conducted in R version 4.4.1 using standard packages for data management, regression, robust variance estimation, and table and figure generation. Field mappings for the 4 core EHR measures and changes in federal definitions over time are summarized in Table S2. Analytic code is available in a public GitHub repository.^38^

## Results

### Study sample and hospital characteristics

**Figure 1.**
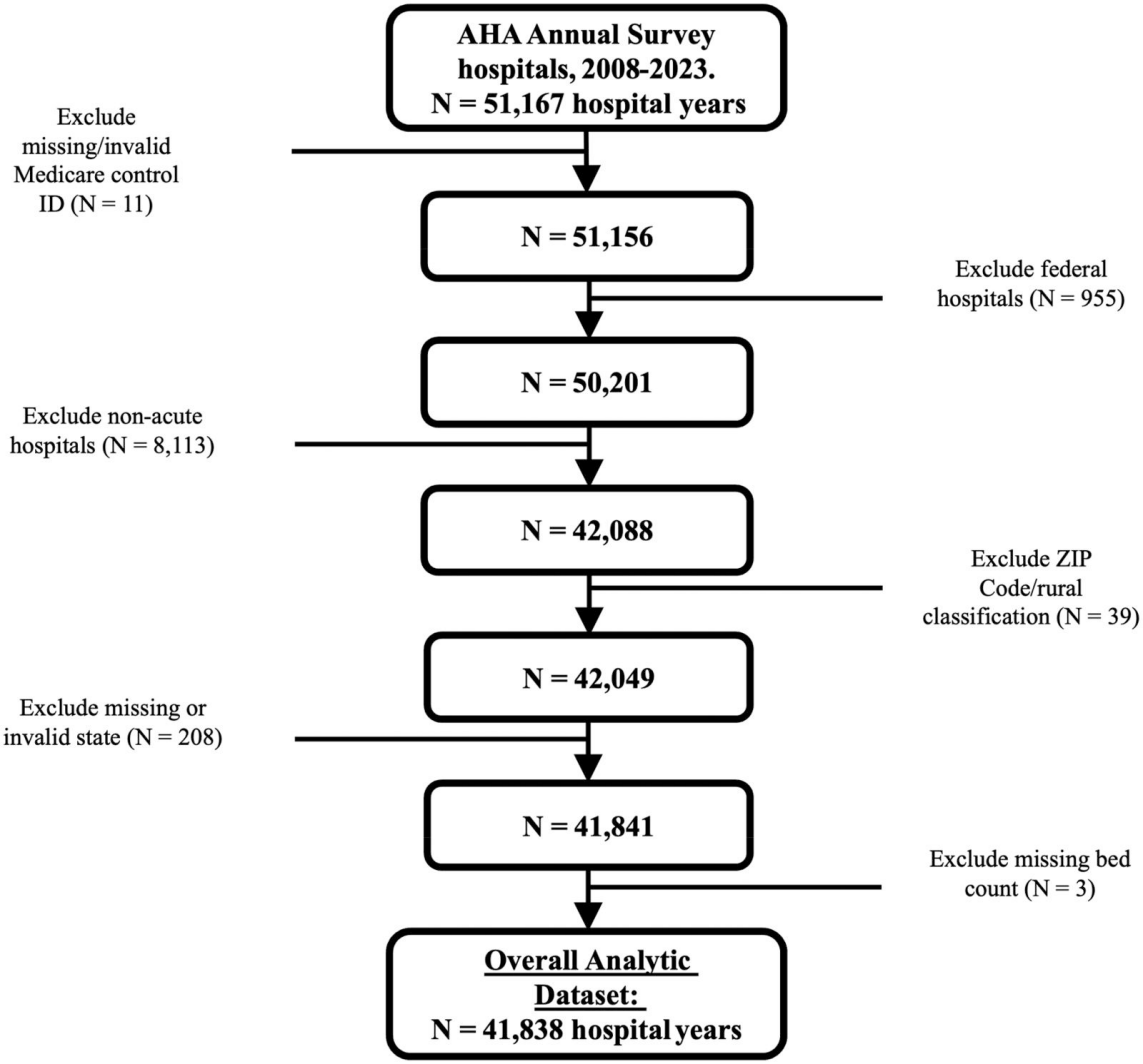
Flow of hospital-year observations into the analytic cohort, 2008-2023. Flow diagram showing the number of hospitals in the initial AHA Annual Survey frame, exclusions based on non-acute or federal status, and missing key variables, and the resulting analytic sample of non-federal acute care hospital-year observations.

After applying inclusion and exclusion criteria (Figure 1), the analytic cohort included 41 838 hospital-year observations from non-federal acute care hospitals between 2008 and 2023. Across all years, 22 852 hospital-years (55%) were classified as urban, and 18 986 (45%) as rural. The number of contributing hospitals per year ranged from 2363 to 3236 (Table 1). Urban hospitals were larger than rural hospitals (median 228 beds, IQR 126-376 vs 43 beds, IQR 25-88), and small hospitals were more common in rural areas (41% vs 5.5%). Ownership patterns differed: rural hospitals were more often government-owned and less often investor-owned. Rural hospitals were more often located in the West North Central, Mountain, and East South Central divisions. Urban hospitals were more often located in the South Atlantic, Middle Atlantic, and Pacific divisions.

**Table 1.** Characteristics of non‑federal acute care hospitals and core EHR adoption, by rurality, 2008‑2023.

Characteristic	Urban, *N* = 22 852	Rural, *N *= 18 986	Total, *N* = 41 838	***P*-value** ^a^
**Survey year** ^b^				<.001
2008	1540 (54%)	1326 (46%)	2866 (100%)	
2009	1691 (52%)	1545 (48%)	3236 (100%)	
2010	1582 (52%)	1435 (48%)	3017 (100%)	
2011	1422 (53%)	1271 (47%)	2693 (100%)	
2012	1572 (54%)	1351 (46%)	2923 (100%)	
2013	1479 (54%)	1244 (46%)	2723 (100%)	
2014	1538 (56%)	1217 (44%)	2755 (100%)	
2015	1574 (55%)	1275 (45%)	2849 (100%)	
2016	1621 (55%)	1303 (45%)	2924 (100%)	
2017	1631 (57%)	1233 (43%)	2864 (100%)	
2018	1620 (56%)	1254 (44%)	2874 (100%)	
2019	^c^	^c^	^c^	
2020	1508 (57%)	1150 (43%)	2658 (100%)	
2021	1336 (56%)	1027 (44%)	2363 (100%)	
2022	1351 (53%)	1197 (47%)	2548 (100%)	
2023	1387 (54%)	1158 (46%)	2545 (100%)	
**Beds (count; Median [IQR])**	227 [125, 375]	43 [25, 88]	114 [36, 258]	<.001
**Beds (categories)**				<.001
≤25	1254/22 852 (5.5%)	7784/18 986 (41%)	9038/41 838 (22%)	
26-100	3134/22 852 (14%)	7371/18 986 (39%)	10 505/41 838 (25%)	
101-300	10 184/22 852 (45%)	3566/18 986 (19%)	13 750/41 838 (33%)	
301-500	5086/22 852 (22%)	231/18 986 (1.2%)	5317/41 838 (13%)	
>500	3194/22 852 (14%)	34/18 986 (0.2%)	3228/41 838 (7.7%)	
**Census division**				<.001
East North Central	3882/22 852 (17%)	3192/18 986 (17%)	7074/41 838 (17%)	
East South Central	1238/22 852 (5.4%)	1557/18 986 (8.2%)	2795/41 838 (6.7%)	
Middle Atlantic	3029/22 852 (13%)	946/18 986 (5.0%)	3975/41 838 (9.5%)	
Mountain	1403/22 852 (6.1%)	1714/18 986 (9.0%)	3117/41 838 (7.5%)	
New England	1058/22 852 (4.6%)	660/18 986 (3.5%)	1718/41 838 (4.1%)	
Pacific	2818/22 852 (12%)	1181/18 986 (6.2%)	3999/41 838 (9.6%)	
South Atlantic	4457/22 852 (20%)	1794/18 986 (9.4%)	6251/41 838 (15%)	
West North Central	1951/22 852 (8.5%)	5359/18 986 (28%)	7310/41 838 (17%)	
West South Central	3016/22 852 (13%)	2583/18 986 (14%)	5599/41 838 (13%)	
**Ownership (non-federal)**				<.001
Non-government nonprofit	16 484/22 852 (72%)	11 135/18 986 (59%)	27 619/41 838 (66%)	
Investor-owned (for-profit)	3596/22 852 (16%)	1335/18 986 (7.0%)	4931/41 838 (12%)	
Government—nonfederal	2772/22 852 (12%)	6516/18 986 (34%)	9288/41 838 (22%)	
**Primary service category**				<.001
General medical and surgical	22 132/22 852 (97%)	18 986/18 986 (100%)	41 118/41 838 (98%)	
Cancer	143/22 852 (0.6%)	0/18 986 (0%)	143/41 838 (0.3%)	
Children’s general	577/22 852 (2.5%)	0/18 986 (0%)	577/41 838 (1.4%)	
**Basic (with notes)**	11 475/18 778 (61%)	7767/15 604 (50%)	19 242/34 382 (56%)	<.001
(Missing)	4074	3382	7456	
**Basic (without notes)**	12 607/18 778 (67%)	8417/15 604 (54%)	21 024/34 382 (61%)	<.001
(Missing)	4074	3382	7456	
**Comprehensive**	7243/18 778 (39%)	3790/15 604 (24%)	11 033/34 382 (32%)	<.001
(Missing)	4074	3382	7456	
**Certified**	17 770/18 591 (96%)	14 055/14 863 (95%)	31 825/33 454 (95%)	<.001
(Missing)	4261	4123	8384	

a Pearson’s Chi-squared test.

b Percentages use within-year denominators of observed responses for survey year (*N* [row%]) and across all hospital-years for remaining characteristics; no AHA Health IT survey was fielded for 2019; missing shown where present.

c No Health IT supplement representing 2019 was fielded by the AHA.

Baseline characteristics of the analytic cohort and cross-sectional distributions of core EHR adoption measures, summarized overall, and stratified by urban vs rural status.

### Core EHR adoption

Core EHR adoption was higher in urban hospitals than in rural ones (Figure 2A). Across all hospital-years, 61% met criteria for a basic EHR without notes (67% of urban vs 54% of rural hospital-years; *P* < .001), 56% met criteria for a basic EHR with notes (61% vs 50%; *P < *.001), and 32% met criteria for comprehensive EHR capability (39% vs 24%; *P* < .001). Adoption of CEHRT was high at 95%, with a small but significant urban-rural difference (96% vs 95%; *P* < .001).

**Figure 2.**
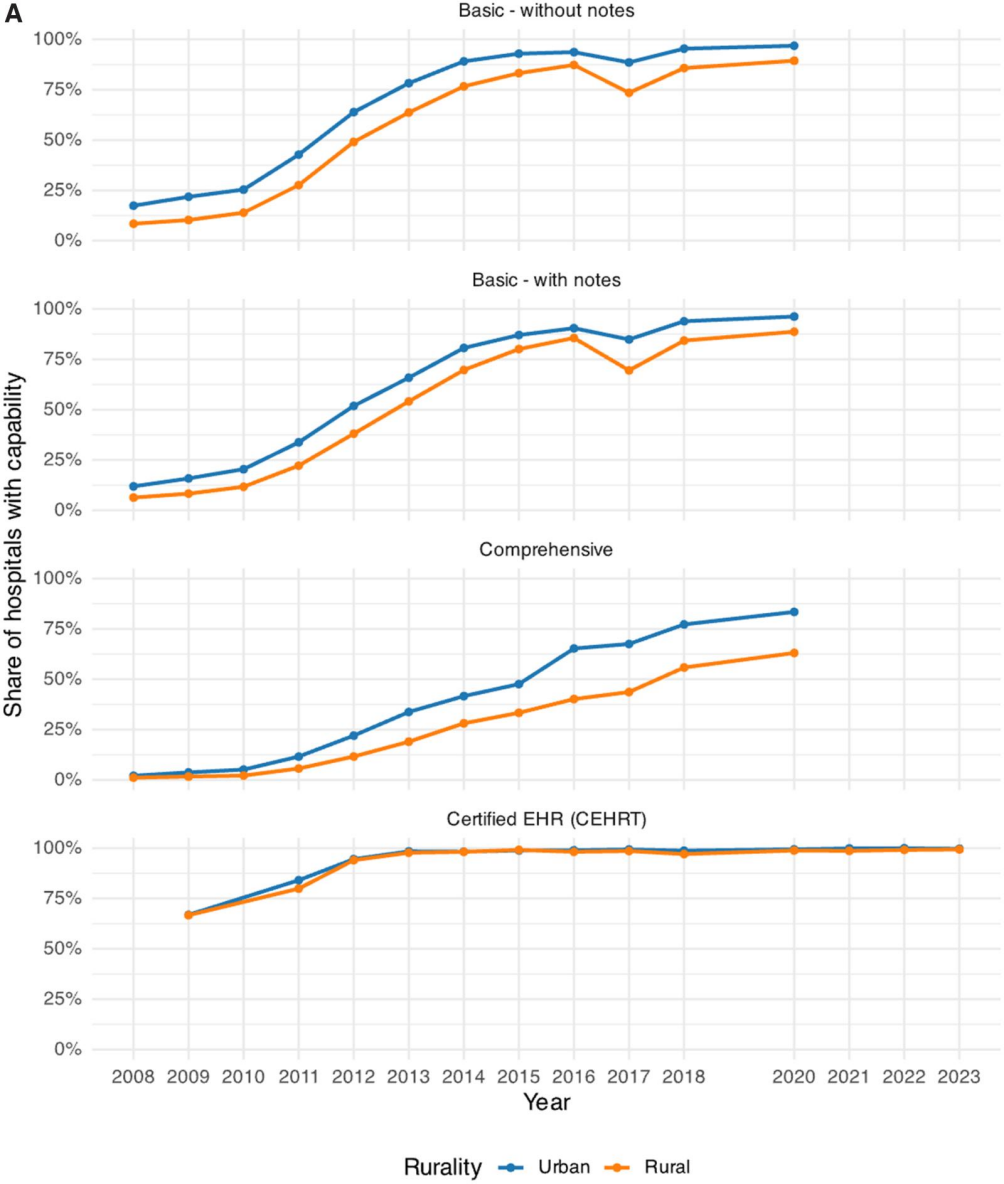
Core EHR adoption by rurality and rural–urban gaps, 2008-2023. (A) Core EHR adoption over time by rurality, 2008-2023. (B) Rural‑urban gaps in core EHR adoption over time, 2008‑2023. Panel A shows the share of hospitals reporting each core EHR capability (basic with notes, basic without notes, comprehensive, and CEHRT) by survey year, by urban vs rural status. Panel B shows the rural–urban percentage-point differences in adoption for each core EHR capability by survey year. Negative values indicate lower adoption in rural hospitals than in urban hospitals; zero indicates parity. Each panel displays the percentage of respondents who reported that hospitals had the capability in that year.

**Figure 2.**
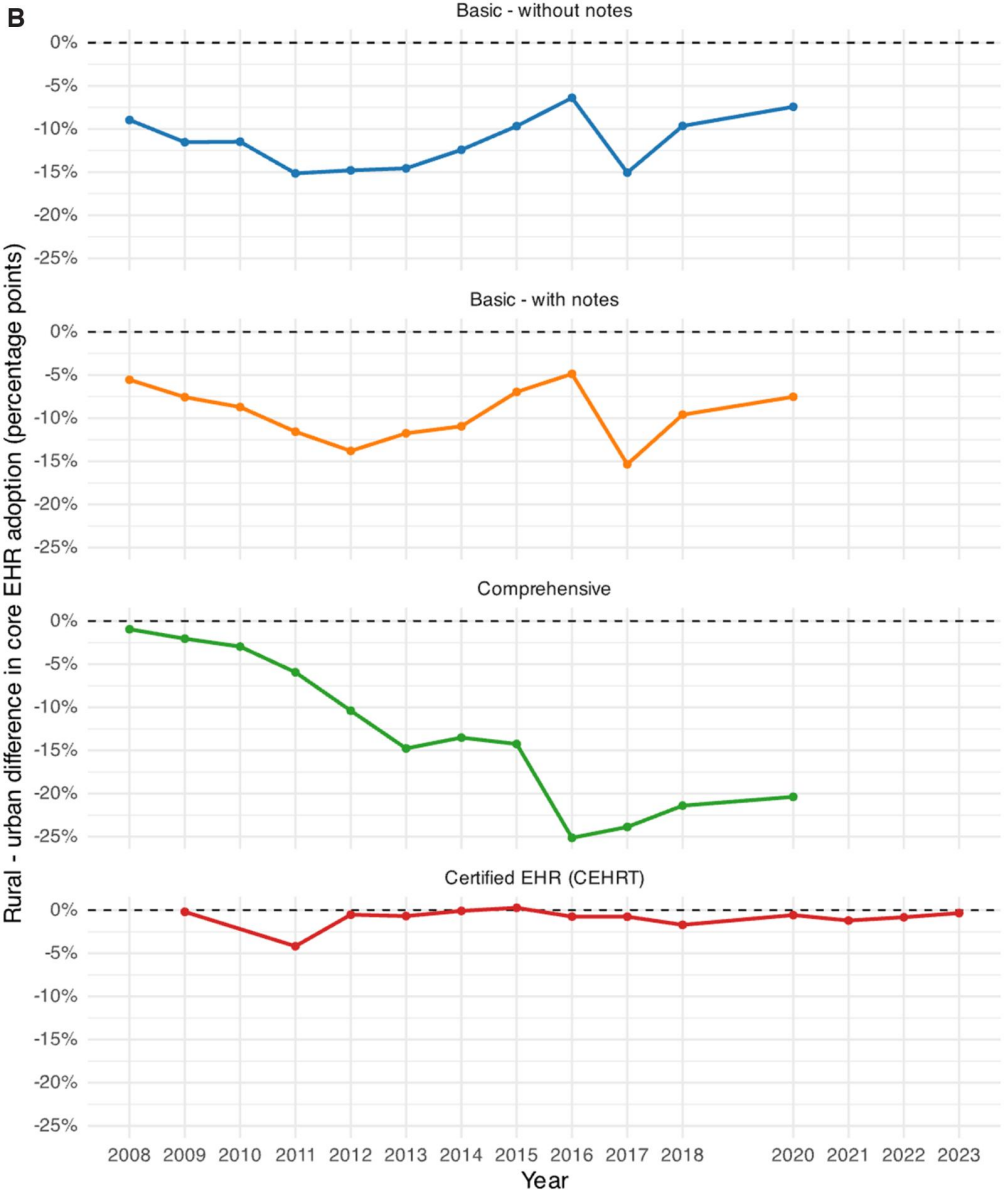
Continued.

Core EHR adoption increased over time (Figure S1 and Table S3). Basic EHRs without notes increased from 13% in 2008 to 94% in 2020. Basic EHRs with notes grew from 9% to 93%. Comprehensive capability rose from 2% in 2008 to 75% in 2020. CEHRT status, first measured in 2009 and thereafter in 2011, increased from 67% in 2008 to 94% in 2012, with nearly complete adoption by 2023.

### Rural–urban differences in core EHR adoption

Rural–urban differences in core EHR adoption persisted throughout the study period, especially for comprehensive capability (Figure 2B; Table S4). Rural hospitals lagged urban hospitals in basic EHR adoption without notes by 9.0% in 2008, with differences ranging from 6 to 15 PP. By 2020, the gap had narrowed to 7.4%. A comparable pattern was observed for basic EHR adoption with notes, where rural hospitals trailed urban hospitals by 5-15 PP in most years. This gap peaked at 15.4% in 2017 and was 7.5% in 2020.

Comprehensive EHR capability showed the largest rural–urban gaps. During the early stages of nationwide EHR adoption, the difference was small. As adoption increased, the gap widened, reaching 10.4% by 2012 and 15-25 PP from 2013 to 2020, peaking at 25.1% in 2016. CEHRT adoption gaps were small (0.2%-4.2% from 2009 to 2011), then remained within ∼±2%; by 2023, the gap was −0.3% (99% vs 100%).

### Advanced EHR use

Advanced EHR use was more variable across functions and less complete than core adoption (Figure 3A; Figure S2 and Table S5). By 2021-2023, *patient portal* functions were widely available. In 2023, 95% of hospitals allowed patients to view clinical notes, 65% accepted patient-generated data, 86% supported application access via APIs, 74% reported FHIR-based application support, and 94% enabled secure messaging. *Interoperability* measures also increased over 2012-2023: querying external organizations rose from 40% to 89% of hospitals, use of outside information often or sometimes increased from 52% to 80%, outside information available electronically at the point of care increased from 42% to 77%, and integration of external summaries increased from 41% to 81%. Integration of prescription benefit information increased from 65% in 2022 to 72% in 2023. *Public health reporting* functions expanded in the early 2020s; by 2022, 79% of hospitals reported engaging in electronic case reporting, 94% immunization registry reporting, and 90% electronic laboratory reporting. *SDOH capabilities* were also common in 2023: 89% of hospitals collected social needs data. Among hospitals that collected social needs data, 82% used SDOH data for clinical decisions, and 70% reported using SDOH data for analytics or population health.

**Figure 3.**
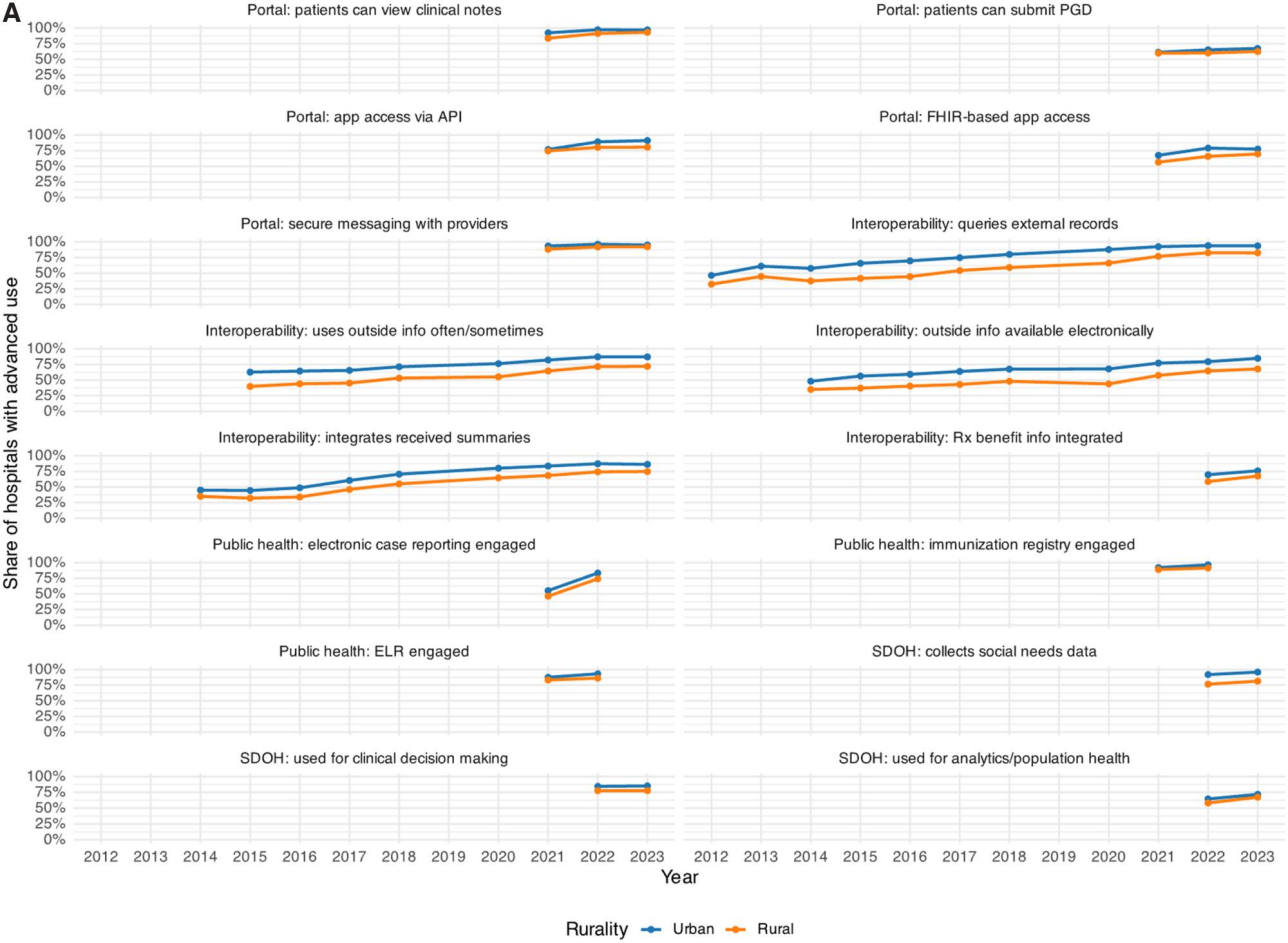
Advanced EHR use by rurality and rural–urban gaps, 2008-2023. (A) Advanced EHR use over time by rurality. (B) Rural‑urban gaps in advanced EHR use over time. Panel A shows time trends in advanced EHR use measures from the AHA IT supplement, by urban vs rural hospitals. Panels display patient engagement functions (portal capabilities), interoperability measures (use, availability, and integration of external information), public health reporting, and SDOH functions for the years in which each item was fielded. Panel B shows rural–urban percentage-point differences in advanced EHR use for each portal, interoperability, public health, and SDOH measure. Panels highlight the magnitude and direction of gaps for each capability across the years in which the item was fielded.

**Figure 3.**
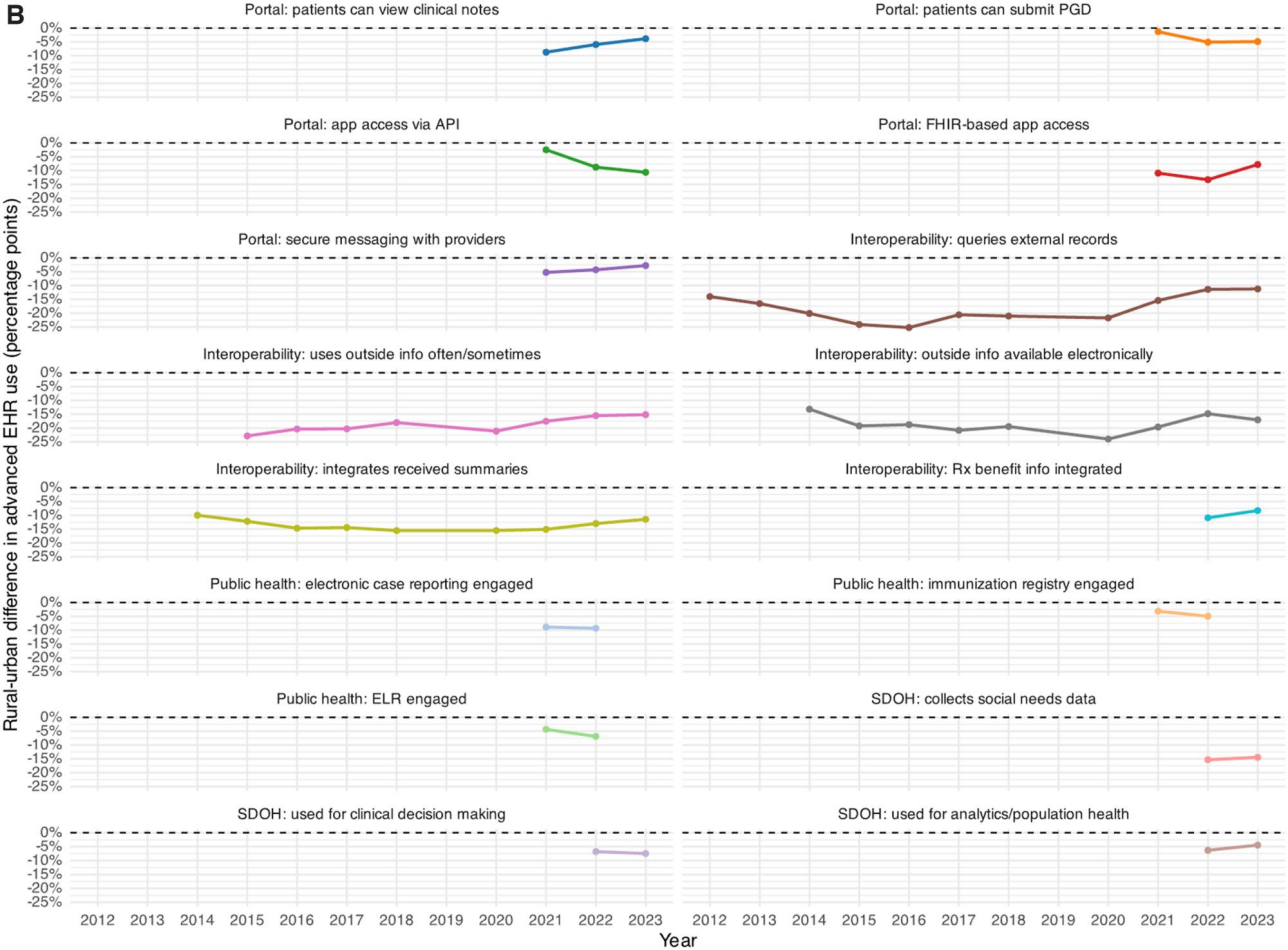
Continued.

### Rural–urban differences in advanced EHR use

Rural and urban patterns in advanced EHR use paralleled the core adoption findings (Figure 3B; Table S5). For *patient engagement* in 2023, adoption was high in both rural and urban hospitals, with rural hospitals 3-11 PP lower across note viewing, patient-generated data, API-based access, FHIR-based applications, and secure messaging. Rural–urban differences were larger for *interoperability* functions: rural hospitals were typically 5-15 PP less likely than urban hospitals to query external records, use outside information often or sometimes, or have external summaries and prescription benefit information integrated into their EHRs.

*Public health reporting* and *SDOH capabilities* were also more frequent in urban hospitals. In 2022, electronic case reporting, immunization registry reporting, and electronic laboratory reporting were each ∼5-9 PP higher in urban hospitals than in rural hospitals. In 2023, rural hospitals were 14.5 PP less likely to report collecting social needs data and 7.5 PP less likely to report using SDOH information in clinical decision making; differences in SDOH data use for analytics and population health were smaller but still present (4.5 PP).

### Trend tests and regression models

Cochran-Armitage tests indicated statistically significant increasing temporal trends for all 4 core EHR measures overall and within both urban and rural strata (*P* < .001 for all tests; Table S6). For advanced EHR use, most portal, interoperability, public health reporting, and SDOH collection measures also showed statistically significant increasing trends overall and by rurality. Trend tests for SDOH use measures were not reported because those items were fielded only in the most recent years (Table S7).

Logistic regression models provided summary estimates of time trends and rural–urban differences in core EHR adoption (Table S8). In adjusted models for urban hospitals, each additional survey year was associated with higher odds of possessing each core capability (adjusted OR 1.58-1.76 for basic with notes, basic without notes, comprehensive, and CEHRT). Year-by-rural interaction terms were consistently below 1.0 for basic and comprehensive EHRs (adjusted interaction OR 0.92-0.95) and for CEHRT (0.87), indicating that annual increases in adoption odds were smaller in rural hospitals than in otherwise similar urban hospitals. Segmented logistic models that allowed changes in slope after 2011 or 2014 fit some measures better than models with a single linear trend, particularly during early adoption periods, but did not materially change the pattern of increasing adoption over time with persistent rural–urban differences.

### Regional differences and 4-category rurality

**Figure 4.**
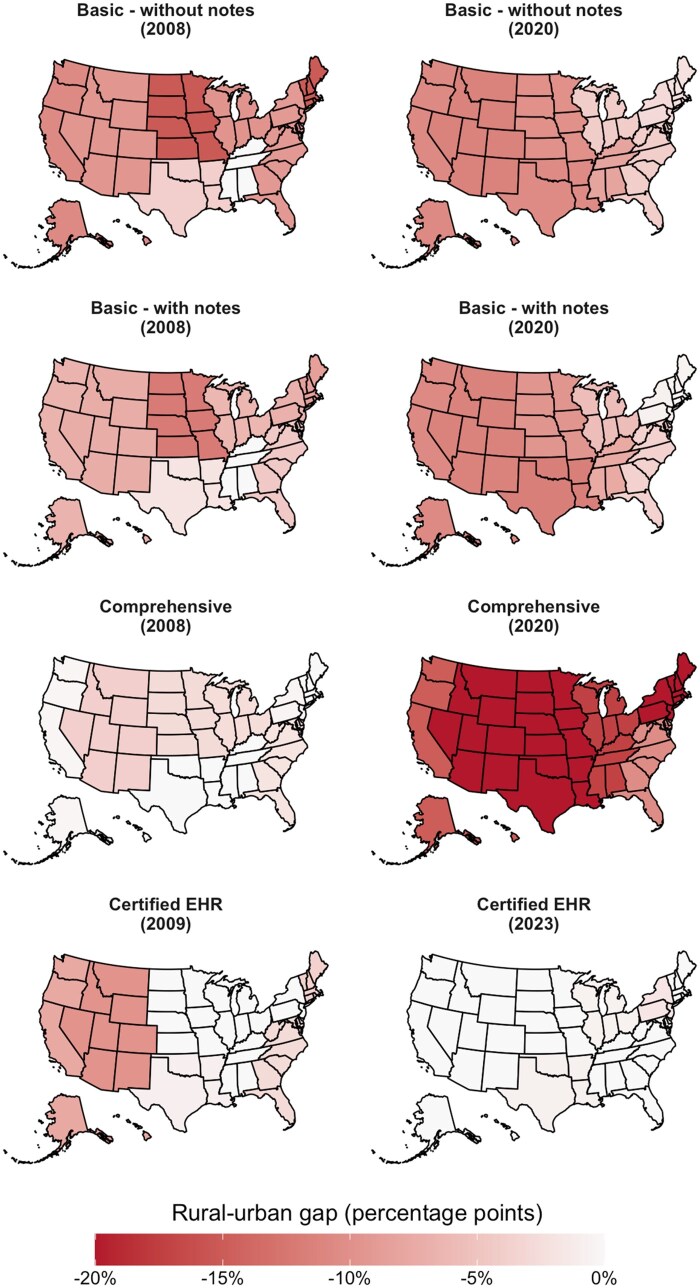
Rural–urban gaps in core EHR adoption by US Census division, 2008-2023. Choropleth maps show rural–urban differences in hospital EHR capabilities by Census division at early and latest survey years. Each division is shaded based on the percentage-point gap, with a color scale from −20 to 0. Darker shades indicate larger rural deficits; lighter shades show minimal gaps.

Regional differences in rural–urban gaps in core EHR adoption were modest for basic EHR and CEHRT and larger for comprehensive EHR (Table S9). Over the study window, rural–urban gaps increased for nearly all measures across Census divisions (Figure 4). For basic EHR with notes, rural hospitals had lower adoption than urban hospitals across divisions, with gaps of roughly 5%-20% in 2014 that narrowed by 2018 and 2020, with smaller gaps in New England and the Middle Atlantic. Comprehensive EHR gaps were larger and more persistent, ranging from roughly 8% to 30% in 2014 and remaining substantial in several divisions in 2018 and 2020. CEHRT adoption showed minimal rural–urban variation across divisions, generally within ±5%, and was nearly universal by 2020. Regression models stratified by Census division showed little evidence of meaningful baseline differences in EHR adoption after adjustment, with rural–urban odds ratios close to 1.0 in most divisions (Table S10).

**Figure 5.**
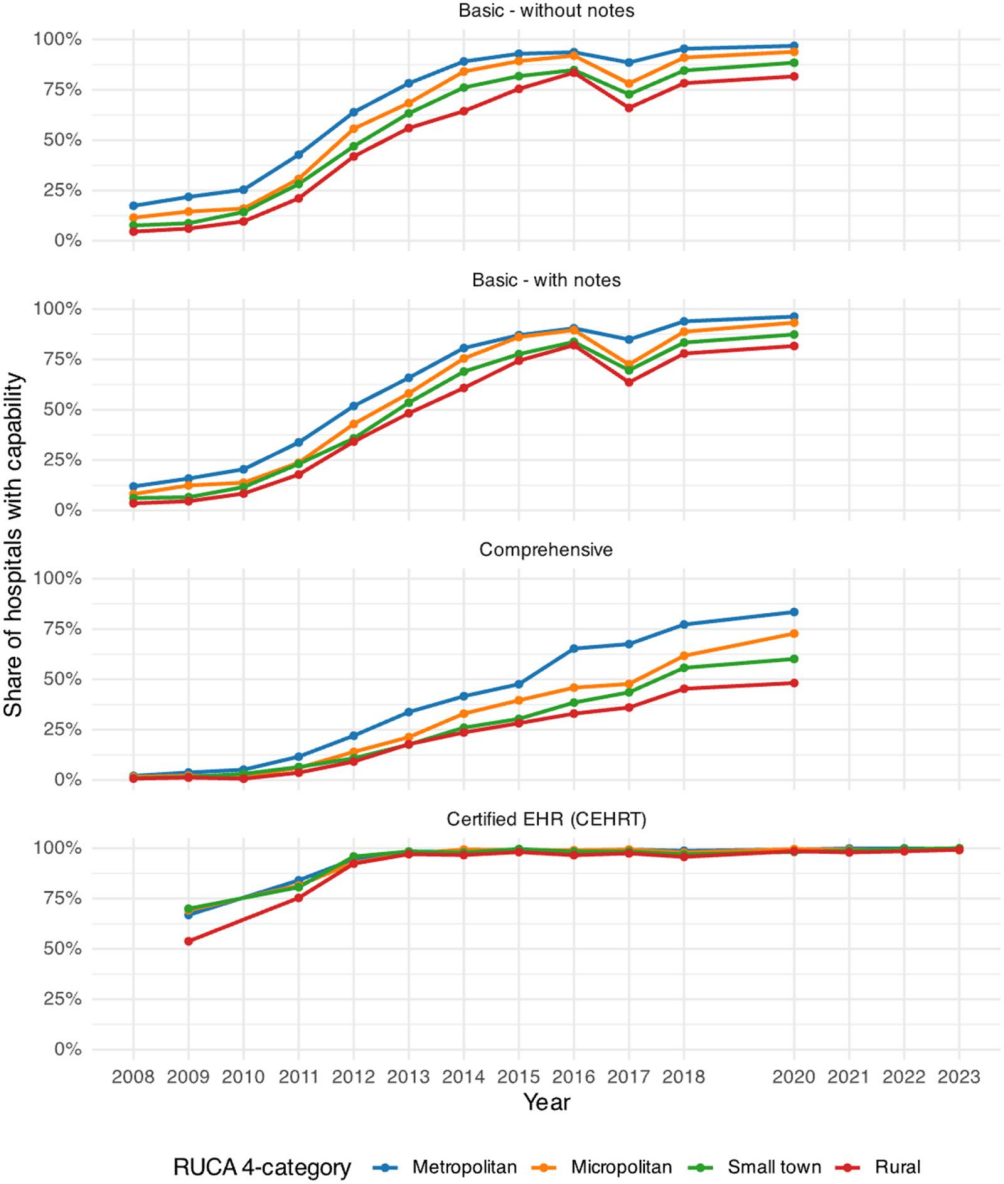
Core EHR adoption over time by 4-categories of rurality, 2008-2023. Share of hospitals reporting each core EHR capability (basic with notes, basic without notes, comprehensive, and CEHRT) by survey year, by 4 category RUCA rurality (metropolitan, micropolitan, small town, and rural). Each panel displays the percentage of respondents whose hospitals reported having the indicated capability in that year; lines within panels show adoption for metropolitan, micropolitan, small-town, and rural hospitals separately.

Analyses using a categorical rural measure showed a gradient in core adoption across metropolitan, micropolitan, small-town, and rural hospitals (Figure 5; Table S11). For basic EHR with notes, metropolitan hospitals had the highest adoption in each year, with micropolitan, small-town, and rural hospitals progressively lower; in 2014, adoption was 81% in metropolitan hospitals compared with 75%, 69%, and 61% in micropolitan, small-town, and rural hospitals, respectively. Gaps narrowed over time, but remained largest for comprehensive EHR capability, with rural hospitals ∼18% behind metropolitan hospitals in 2014 and more than 30% behind by 2018-2020. CEHRT adoption was high across all RUCA categories by 2014, with differences of only a few percent. In regression models (Table S12), year-by-RUCA interactions were near 1.0 for micropolitan hospitals and below 1.0 for small-town and rural hospitals, indicating smaller annual increases in adoption odds outside urban areas.

### Sensitivity analyses

In a sensitivity analysis restricted to 2011-2015, we evaluated survey participation and the impact of nonresponse weighting. Overall participation ranged from 56% to 62% across these years, with modest, inconsistent rural–urban differences that did not exceed 5 PP in any year (Table S13). Inverse-probability weights derived from annual survey characteristics were stable after trimming and scaling (mean 1.6-1.8; 99th percentile ≤4.6; Table S14). Weighted logistic regression models yielded estimates highly similar to those from unweighted models across all core adoption measures, including rural–urban contrasts and time trends, and no substantive inferences changed with weighting (Table S15).

Using stricter public health reporting definitions, rates were lower when limited to production submissions or submissions sent directly from the EHR; for electronic case reporting, production reporting was 53% in 2022, and EHR-direct reporting was 60% in 2023. Rural–urban gaps widened under the EHR-direct definition (2023: 71% urban vs 46% rural; −24.6 PP), with similar patterns for immunization registry and laboratory reporting (Table S16). For social needs, 80% of hospitals reported structured electronic recording in 2023, while 76% reported screening with structured electronic recording; rural–urban differences were minimal for recording but persisted for screening (Table S16).

Annualized growth analyses showed mixed convergence: across 16 measures, gaps narrowed for 8 and widened for 8. The largest narrowing was the use of outside information often or sometimes (gap improved by 7.7 PP, 2015-2023), while the largest widening was patient app access via an API (gap worsened by 8.1 PP, 2021-2023; Table S17).

## Discussion

In this repeated cross-sectional study of non-federal acute care hospitals, core EHR adoption was high by 2020, and CEHRT adoption was nearly universal by 2023, but rural hospitals lagged in advanced EHR use. The comprehensive EHR adoption gap between rural and urban hospitals peaked at over 25 PP in 2016 and remained significant through 2020. Currently, rural–urban gaps persist in advanced use related to interoperability, public health reporting, and health-related social risk. The sensitivity analyses using EHR-direct public health reporting and structured electronic social-needs documentation support this interpretation. Although some activities can occur outside the EHR, the advanced measures in this study reflect noticeable rural–urban disparities in EHR-enabled capabilities and related workflows.

Prior work documented rapid gains in EHR capabilities over the decade after the HITECH Act, noting significant variation in adoption by hospital size, ownership, and teaching status. Early in the adoption process, significant gaps were evident: smaller and rural hospitals were less likely to meet basic or comprehensive EHR standards, while advanced functionalities were mainly found in larger, urban, and system-affiliated facilities.^3^^,^^7^ More recent federal monitoring indicates that nearly all non-federal acute care hospitals now report using certified EHR systems, and most have at least some capacity to exchange information across organizational boundaries.^2^^,^^25^ Our study extends this research through 2023, differentiates between core adoption and advanced use, and explicitly examines differences between rural and urban hospitals beyond variation in organizational characteristics.

The persistent gap we observed in advanced use aligns with earlier evidence that adopting certified systems alone is insufficient to ensure robust clinical use of key functions. Advanced EHR capabilities, such as computerized provider order entry with clinical decision support, have been associated with reductions in medication errors, more appropriate prescribing, and improved adherence to evidence-based care.^8^^,^^39^ EHR‑tethered tools, including patient portals, that enable patients to view results, exchange secure messages, and share home‑collected data have been associated with better chronic disease self‑management, improved adherence to preventive services and medications, and higher satisfaction with care.^40–42^ Interoperability and health information exchanges (HIE) have been linked to fewer duplicative tests, more informed decision-making, and lower rates of avoidable utilization.^18^^,^^43^

These advanced capabilities are central to the implementation of an LHS,^44^ in which routine care processes generate data that can be analyzed and fed back to clinicians, health systems, and policymakers. The LHS framework emphasizes digital infrastructure, interoperable data flows, and feedback loops as prerequisites for continuous improvement in quality, safety, and value.^45^^,^^46^ EHR-based decision support, population health dashboards, and automated public health reporting all enable measurement and timely feedback on clinical performance.^47^^,^^48^ Structured data on social risk and patient experience can inform targeted interventions.^49^^,^^50^ When rural hospitals lack these capabilities, they are less able to participate fully in regional learning collaboratives, statewide quality improvement initiatives, and value-based payment models that depend on robust data capture and exchange.^51^ Communities facing higher chronic disease, workforce shortages, and financial constraints may also have fewer opportunities to benefit from an LHS.

The rural advanced use gap observed in this study likely reflects a combination of financial, technical, and organizational factors. Rural hospitals tend to operate with lower margins, more limited access to capital, and smaller technical teams than urban facilities.^52^^,^^53^ Implementing advanced functions often requires substantial configuration, workflow redesign, and ongoing analytical support, all of which are more difficult to sustain for small organizations with high staff turnover and limited access to specialist expertise.^54^ Contracting for participation in HIE, public health interfaces, or third-party analytic platforms may introduce additional recurring costs.^55^ Vendor product configurations and optional interface or module pricing may further shape hospitals’ ability to operationalize advanced capabilities, particularly API/FHIR functions and public health reporting interfaces. Recent rural hospital redesign efforts, including the emergence of rural emergency hospital^56^ designations, may also influence investment capacity and priorities for advanced health IT. Even when core functionality is in place, these barriers can slow or prevent the transition from basic adoption to sophisticated use, reinforcing a digital gradient across the rural–urban continuum.

These findings have several implications for policy and practice. Incentive programs focused solely on certified adoption are unlikely to close the advanced use gap. Programs emphasizing interoperability and patient access could provide rural-targeted incentives, technical assistance, or shared services that reduce fixed implementation costs, and regional partners can support implementation and training tailored to smaller hospitals,^57^ particularly in light of recent CMS initiatives such as the Rural Health Transformation Program,^58^ which may create opportunities for targeted investment in rural digital infrastructure. Payers and regulators could align measures and reporting with staged development of advanced use. Measurement should move beyond adoption toward routine assessment of how advanced functions support coordination and engagement.^59^^,^^60^

From a rural health equity perspective, closing the advanced use gap is particularly important. Rural hospitals often serve populations with higher levels of clinical complexity and social risk. They also often function as the only source of emergency, inpatient, and outpatient care in their communities.^61^ Limited use of advanced EHRs in these hospitals may result in fewer opportunities to identify gaps in preventive care, manage chronic disease across fragmented care networks, or respond rapidly to public health threats.^47^^,^^62^ The COVID-19 pandemic highlighted the importance of timely electronic case reporting, immunization registry interfaces, and analytic dashboards for situational awareness.^63^ Persistent rural shortfalls in these capabilities may leave communities less prepared for future emergencies and less able to participate in new models of care that depend on data sharing and analytics, such as remote patient monitoring and electronic dashboards to improve patient outcomes.^64^

### Limitations

This study has several limitations. First, the analysis relied on self-reported data from a voluntary survey, which may be subject to nonresponse bias and misclassification. Prior analyses of the AHA-IT Supplement suggest that weighting does not materially affect national adoption estimates.^3^^,^^65^^,^^66^ Accordingly, we present unweighted models with hospital-clustered standard errors. In a sensitivity analysis limited to 2011-2015, when the full AHA Annual Survey was available to construct nonresponse weights, weighted models yielded results that were substantively similar to the primary analyses, with no meaningful changes in rural–urban differences or time trends. Second, the measures assessed whether hospitals had specific functionalities available, rather than how consistently or effectively those functions were used. Third, there are inherent limitations in assuming that rural challenges are universal. Rurality is primarily classified using population-density measures. More nuanced measures of rurality^67^,^68^ or a classification of hospital catchment areas^69^ would be preferable, but were not available for this study. Fourth, the study focused on a set of EHR functions; other capabilities, such as advanced analytics or specialty-specific tools, were not examined. Finally, the observational design precludes causal inference regarding the impact of policies or market forces on rural–urban differences in advanced EHR use.

## Conclusions

This national analysis of hospitals indicates that the United States has largely achieved widespread adoption of core EHR systems in both rural and urban settings. However, there remains a persistent gap in advanced EHR use between rural and urban hospitals for interoperability, patient engagement, public health reporting, and use of social risk information. Addressing this advanced-use divide will require policy, research, and implementation efforts that move beyond adoption to focus on the capabilities that enable an LHS, supporting equitable digital transformation in rural settings, including the ability to capture, exchange, and act on structured social risk information to support health equity.

## Supplementary Material

ocag043_Supplementary_Data

## Data Availability

The data used in this study were obtained from the AHA as part of its annual Health IT supplement. More information on accessing the data is available here: https://www.ahadata.com/aha-healthcare-it-database. File layouts and survey instruments are publicly available: https://www.ahadata.com/aha-data-resources.

## Code availability

All code used for data preparation and statistical analyses was written in R (version 4.4.1). Reproducible scripts are available in a public GitHub repository: https://github.com/Anzalone-Lab/AHA-Project. This includes file layouts for all AHA Health IT database years used, processing code to harmonize field names across years, and all statistical analysis code used in this study.
