# Supplementary material for: The digital divide in adoption and advanced use of electronic health records among US hospitals: rural versus urban disparities from 2008 to 2023
Source: J Am Med Inform Assoc. 2026 Apr 27;33(7):1354–69. doi: 10.1093/jamia/ocag043 (PMC13317969; doi:10.1093/jamia/ocag043)
Supplement: ocag043_Supplementary_Data [file ocag043_supplementary_data.docx]

**Supplemental Materials - Table of Contents**

**I. Supplemental Tables**

**Supplemental Table 1.** AHA Health IT Yearly Survey Availability, Number of Responses, and Approximate Response Rate

**Supplemental Table 2.** AHA IT survey item mappings and coding for core EHR adoption and advanced use indicators

**Supplemental Table 3.** Core EHR adoption, 2008‑2023

**Supplemental Table 4.** Rural-urban differences in core EHR adoption over time, 2008-2023

**Supplemental Table 5.** Advanced EHR use over time, 2012-2023

**Supplemental Table 6.** Tests for linear trends in core EHR adoption, 2008-2023

**Supplemental Table 7.** Tests for linear trends in advanced EHR use, 2012-2023

**Supplemental Table 8.** Logistic regression for core functionality, 2008-2023

**Supplemental Table 9.** Core EHR adoption by Census division and rurality, 2008-2023

**Supplemental Table 10.** Logistic regression models for core EHR adoption by Census division, 2008-2023

**Supplemental Table 11.** Core EHR adoption by four‑category RUCA rurality, 2008-2023

**Supplemental Table 12.** Logistic regression models for core EHR adoption using four-category RUCA rurality, 2008-2023

**Supplemental Table 13.** Health IT Survey Response Rates, 2011-2015

**Supplemental Table 14.** Distribution of Nonresponse Weights, 2011-2015

**Supplemental Table 15.** Weighted vs Unweighted Core Adoption Models, 2011-2015 **Supplemental Table 16.** Sensitivity analyses for public health reporting and structured electronic social needs capture, 2021-2023

**Supplemental Table 17.** Annualized rural and urban growth rates in advanced EHR use measures, 2012-2023

**II. Supplemental Figures**

**Supplemental Figure 1.** Core EHR adoption over time - overall

**Supplemental Figure 2.** Advanced EHR use over time - overall

**Supplemental Table 1. AHA Health IT Yearly Survey Availability, Number of Responses, and Approximate Response Rate**

| **Year on IT Survey** | **Year IT Data Represent** | **Year IT Data Released** | **N Responses** | **Approximate Response Rate** |
| --- | --- | --- | --- | --- |
| 2023 | 2023 | 2024 | 3128 | 51.28% |
| 2022 | 2022 | 2023 | 3127 | 51.26% |
| 2020 | 2021 | 2022 | 2885 | 47.30% |
| 2019 | 2020 | 2021 | 3374 | 55.31% |
| 2018 | 2018 | 2019 | 3540 | 58.03% |
| 2017 | 2017 | 2018 | 3548 | 58.16% |
| 2016 | 2016 | 2017 | 3656 | 59.93% |
| 2015 | 2015 | 2016 | 3538 | 58.00% |
| 2014 | 2014 | 2015 | 3307 | 54.21% |
| 2013 | 2013 | 2014 | 3283 | 53.82% |
| 2012 | 2012 | 2013 | 3487 | 57.16% |
| 2010 | 2011 | 2012 | 3233 | 53.00% |
| 2009 | 2010 | 2011 | 3615 | 59.26% |
| 2008 | 2009 | 2010 | 3995 | 65.49% |
| 2007 | 2008 | 2009 | 3451 | 56.57% |

The year on the AHA IT supplement, the year the supplement represents, the year the supplement was released, the number of responses, and the approximate response rate using a baseline of 6,100 AHA member hospitals as of December 2025. The number of hospitals has declined since 2008, so the response rates are likely overestimates in the early years.

**Supplemental Table 2. AHA IT survey item mappings and coding for core EHR adoption and advanced use indicators**

| **Domain** | **Short label** | **AHA IT items (field names)** | **Capability Years** | **Definition** |
| --- | --- | --- | --- | --- |
| EHR adoption | Basic EHR - without notes | CSEDPD, CSEDPL, CSEDML, CSEDDS, CSRVLR, CSRVRR, CSRVDR, CSCPM | 2008-2018, 2020 | Basic EHR functions without physician notes (documentation, results, medication CPOE). |
| EHR adoption | Basic EHR - with notes | CSEDPD, CSEDPL, CSEDML, CSEDDS, CSEDPN, CSEDNA, CSRVLR, CSRVRR, CSRVDR, CSCPM | 2008-2018, 2020 | Basic EHR functions including physician notes, core results, and medication CPOE. |
| EHR adoption | Comprehensive EHR | CSEDPD, CSEDPL, CSEDML, CSEDDS, CSEDPN, CSEDNA, CSEDAD, CSRVLR, CSRVRR, CSRVDR, CSRVRI, CSRVDI, CSRVCR, CSCPM, CSCPLT, CSCPRT, CSCPCR, CSCPNO, CSDSCG, CSDSCR, CSDSDA, CSDSDD, CSDSDL, CSDSDS | 2008-2018, 2020 | Comprehensive EHR: basic functions plus advanced results, CPOE, and decision support. |
| EHR adoption | Certified EHR technology (CEHRT) in use | CCHIT, EMRHRCRT | 2009, 2011-2018, 2020-2023 | Hospital uses CCHIT-certified EHR (2009) or ONC-certified CEHRT (2011+). |
| Patient engagement (portal functionality) | Patients can view clinical notes through a portal | PEFVCNIS, PEFVCNOS | 2021–2023 | Indicator coded 1 if either inpatient (PEFVCNIS) or outpatient (PEFVCNOS) item indicated availability (value = 1). Indicator coded 0 if both items indicated absence. Other responses treated as missing. |
| Patient engagement (portal functionality) | Patients can submit patient-generated data through portal | PEFSDIS, PEFSDOS | 2021–2023 | Indicator coded 1 if either inpatient (PEFSDIS) or outpatient (PEFSDOS) item indicated capability. Indicator coded 0 if both indicated absence. |
| Patient engagement (portal functionality) | Third-party applications can access portal data via API | PEFAPIIS, PEFAPIOS | 2021–2023 | Indicator coded 1 if either inpatient or outpatient API field indicated availability. Indicator coded 0 otherwise. |
| Patient engagement (portal functionality) | Third-party applications can access portal data via FHIR | PEFFHIIS, PEFFHIOS | 2021–2023 | Indicator coded 1 if either inpatient or outpatient FHIR access field indicated availability. Indicator coded 0 otherwise. |
| Patient engagement (portal functionality) | Secure messaging between patients and providers | PERSMIS, PERSMOS | 2021–2023 | Indicator coded 1 if either inpatient or outpatient messaging field indicated availability. Indicator coded 0 otherwise. |
| Interoperability | Queries external organizations for patient information | EQPHIOS | 2012–2023 | Indicator coded 1 if hospital reported querying external organizations electronically (value = 1). All other response categories coded 0. |
| Interoperability | Uses outside electronic health information often or sometimes | PHIOUT | 2015–2023 | Indicator coded 1 if hospital reported using outside information often or sometimes (values = 1 or 2). Indicator coded 0 if responses indicated rarely, never, or do not know (values = 3, 4, or 5). |
| Interoperability | Outside patient information available electronically at point of care | CIAOUT | 2014–2023 | Indicator coded 1 if hospital reported information available electronically (value = 1). Responses indicating no or do not know coded 0. |
| Interoperability | Electronic summaries received from outside organizations are integrated into EHR | SOCINT | 2014–2023 | Indicator coded 1 if hospital reported summaries integrated routinely or not routinely (values = 1 or 2). Indicator coded 0 if responses indicated no, do not know, or not applicable (values = 3–5). |
| Interoperability | Medication benefit information integrated into prescribing workflow | PRESBEN | 2022–2023 | Indicator coded 1 if benefit information was integrated for all or a limited set of payers (values = 1 or 2). Indicator coded 0 if responses indicated no or do not know. |
| Public health reporting | Electronic case reporting engagement | ECRAE | 2021–2022 | Indicator coded 1 if hospital reported actively submitting production data, testing/validating, or completed registration (values = 1–3). Indicator coded 0 if hospital had not completed registration (value = 4). |
| Public health reporting | Immunization registry engagement | IRRAE | 2021–2022 | Coding identical to electronic case reporting engagement (values 1–3 = 1; value 4 = 0). |
| Public health reporting | Electronic laboratory reporting engagement | ERLAE | 2021–2022 | Coding identical to electronic case reporting engagement (values 1–3 = 1; value 4 = 0). |
| Public health reporting (sensitivity) | Electronic case reporting production submission | ECRAE | 2021–2022 | Indicator coded 1 if hospital reported active production submission (value = 1). Other engagement categories coded 0. |
| Public health reporting (sensitivity) | Electronic case reporting sent directly from EHR | ECRESD, ECREHR | 2021–2023 | Indicator coded 1 if submission method indicated transmission directly from the EHR system. |
| Public health reporting (sensitivity) | Electronic case reporting production submission sent from EHR | ECRAE, ECRESD, ECREHR | 2021–2022 | Indicator coded 1 only if hospital both submitted production data and reported transmission directly from the EHR. |
| Public health reporting (sensitivity) | Immunization registry production submission | IRRAE | 2021–2022 | Indicator coded 1 if hospital reported active production submission. |
| Public health reporting (sensitivity) | Immunization registry reporting sent directly from EHR | IRRESD, IIREHR | 2021–2023 | Indicator coded 1 if reporting occurred directly from EHR. |
| Public health reporting (sensitivity) | Immunization registry production submission sent from EHR | IRRAE, IRRESD, IIREHR | 2021–2022 | Indicator coded 1 if hospital reported production submission and EHR-direct transmission. |
| Public health reporting (sensitivity) | Electronic laboratory reporting production submission | ERLAE | 2021–2022 | Indicator coded 1 if hospital reported active production submission. |
| Public health reporting (sensitivity) | Electronic laboratory reporting sent directly from EHR | ERLESD, ERLEHR | 2021–2023 | Indicator coded 1 if reporting occurred directly from EHR. |
| Public health reporting (sensitivity) | Electronic laboratory reporting production submission sent from EHR | ERLAE, ERLESD, ERLEHR | 2021–2022 | Indicator coded 1 if hospital reported production submission and EHR-direct transmission. |
| Social determinants of health | Collects social needs data | SDHSN | 2022–2023 | Indicator coded 1 if hospital reported collecting social needs data routinely or not routinely (values = 1 or 2). Indicator coded 0 if hospital reported not collecting or did not know (values = 3 or 4). |
| Social determinants of health | Social needs data used for clinical decision making | HSNCDM | 2022–2023 | Indicator calculated only among hospitals reporting social needs data collection. Indicator coded 1 if reported use for clinical decisions. |
| Social determinants of health | Social needs data used for analytics or population health | HSNPHA | 2022–2023 | Indicator calculated only among hospitals reporting social needs data collection. Indicator coded 1 if reported use for analytics or population health. |
| Social determinants of health (sensitivity) | Social needs recorded electronically in structured format | SNESTL, SNREC | 2022–2023 | Indicator coded 1 if hospital reported structured electronic recording of social needs. |
| Social determinants of health (sensitivity) | Social needs screening with structured electronic recording | SDHSN, SNESTL, SNREC | 2022–2023 | Indicator coded 1 if hospital both screened for social needs and recorded responses in structured electronic format. |

Conceptual domains, short labels, AHA IT supplement field names, analysis years, and coding rules were used to construct hospital-level indicators of core EHR adoption and advanced EHR use.

**Supplemental Table 3. Core EHR adoption, 2008‑2023**

| **Hospital Strata** | **Year** | **Basic - with notes, % (n/N)** | **Basic - without notes, % (n/N)** | **Comprehensive, % (n/N)** | **Certified EHR, % (n/N)** |
| --- | --- | --- | --- | --- | --- |
| Overall | 2008 | 9% (267/2,866) | 13% (380/2,866) | 2% (47/2,866) | NA |
| Overall | 2009 | 12% (396/3,236) | 16% (528/3,236) | 3% (89/3,236) | 67% (1,483/2,220) |
| Overall | 2010 | 16% (491/3,017) | 20% (602/3,017) | 4% (112/3,017) | NA |
| Overall | 2011 | 28% (762/2,693) | 36% (959/2,693) | 9% (237/2,693) | 82% (1,978/2,408) |
| Overall | 2012 | 45% (1,329/2,923) | 57% (1,667/2,923) | 17% (503/2,923) | 94% (2,497/2,648) |
| Overall | 2013 | 60% (1,647/2,723) | 72% (1,949/2,723) | 27% (735/2,723) | 98% (2,563/2,613) |
| Overall | 2014 | 76% (2,088/2,755) | 84% (2,303/2,755) | 36% (982/2,755) | 98% (2,673/2,722) |
| Overall | 2015 | 84% (2,391/2,849) | 89% (2,523/2,849) | 41% (1,174/2,849) | 99% (2,742/2,771) |
| Overall | 2016 | 88% (2,581/2,924) | 91% (2,655/2,924) | 54% (1,581/2,924) | 99% (2,827/2,867) |
| Overall | 2017 | 78% (2,241/2,864) | 82% (2,350/2,864) | 57% (1,639/2,864) | 99% (2,751/2,779) |
| Overall | 2018 | 90% (2,578/2,874) | 91% (2,620/2,874) | 68% (1,951/2,874) | 98% (2,760/2,816) |
| Overall | 2020 | 93% (2,471/2,658) | 94% (2,488/2,658) | 75% (1,983/2,658) | 99% (2,569/2,591) |
| Overall | 2021 | NA | NA | NA | 99% (2,243/2,258) |
| Overall | 2022 | NA | NA | NA | 100% (2,429/2,440) |
| Overall | 2023 | NA | NA | NA | 100% (2,310/2,321) |

Year-specific adoption of basic with notes, basic without notes, comprehensive, and certified EHR capabilities is presented for all analytic hospitals combined. Cells indicate the percentage of hospitals with the capability and the corresponding n/N. Composite basic and comprehensive measures are unavailable in 2019 and after 2020 because the underlying multi-item batteries were not fielded. Percentages are calculated among hospitals with non-missing data for each capability in the specified year.

**Supplemental Table 4. Rural-urban differences in core EHR adoption over time, 2008-2023**

| **Measure** | **Year** | **Rural hospitals** | **Urban hospitals** | **Gap (Rural − Urban, percentage points)** |
| --- | --- | --- | --- | --- |
| Basic - with notes | 2008 | 6% (84/1,326) | 12% (183/1,540) | -5.5 |
| Basic - with notes | 2009 | 8% (128/1,545) | 16% (268/1,691) | -7.6 |
| Basic - with notes | 2010 | 12% (168/1,435) | 20% (323/1,582) | -8.7 |
| Basic - with notes | 2011 | 22% (282/1,271) | 34% (480/1,422) | -11.6 |
| Basic - with notes | 2012 | 38% (514/1,351) | 52% (815/1,572) | -13.8 |
| Basic - with notes | 2013 | 54% (673/1,244) | 66% (974/1,479) | -11.8 |
| Basic - with notes | 2014 | 70% (848/1,217) | 81% (1,240/1,538) | -10.9 |
| Basic - with notes | 2015 | 80% (1,021/1,275) | 87% (1,370/1,574) | -7 |
| Basic - with notes | 2016 | 86% (1,115/1,303) | 90% (1,466/1,621) | -4.9 |
| Basic - with notes | 2017 | 70% (857/1,233) | 85% (1,384/1,631) | -15.4 |
| Basic - with notes | 2018 | 84% (1,057/1,254) | 94% (1,521/1,620) | -9.6 |
| Basic - with notes | 2020 | 89% (1,020/1,150) | 96% (1,451/1,508) | -7.5 |
| Basic - without notes | 2008 | 8% (112/1,326) | 17% (268/1,540) | -9 |
| Basic - without notes | 2009 | 10% (159/1,545) | 22% (369/1,691) | -11.5 |
| Basic - without notes | 2010 | 14% (200/1,435) | 25% (402/1,582) | -11.5 |
| Basic - without notes | 2011 | 28% (351/1,271) | 43% (608/1,422) | -15.1 |
| Basic - without notes | 2012 | 49% (663/1,351) | 64% (1,004/1,572) | -14.8 |
| Basic - without notes | 2013 | 64% (792/1,244) | 78% (1,157/1,479) | -14.6 |
| Basic - without notes | 2014 | 77% (933/1,217) | 89% (1,370/1,538) | -12.4 |
| Basic - without notes | 2015 | 83% (1,061/1,275) | 93% (1,462/1,574) | -9.7 |
| Basic - without notes | 2016 | 87% (1,137/1,303) | 94% (1,518/1,621) | -6.4 |
| Basic - without notes | 2017 | 73% (906/1,233) | 89% (1,444/1,631) | -15.1 |
| Basic - without notes | 2018 | 86% (1,075/1,254) | 95% (1,545/1,620) | -9.6 |
| Basic - without notes | 2020 | 89% (1,028/1,150) | 97% (1,460/1,508) | -7.4 |
| Comprehensive | 2008 | 1% (15/1,326) | 2% (32/1,540) | -0.9 |
| Comprehensive | 2009 | 2% (26/1,545) | 4% (63/1,691) | -2 |
| Comprehensive | 2010 | 2% (31/1,435) | 5% (81/1,582) | -3 |
| Comprehensive | 2011 | 6% (72/1,271) | 12% (165/1,422) | -5.9 |
| Comprehensive | 2012 | 12% (157/1,351) | 22% (346/1,572) | -10.4 |
| Comprehensive | 2013 | 19% (236/1,244) | 34% (499/1,479) | -14.8 |
| Comprehensive | 2014 | 28% (342/1,217) | 42% (640/1,538) | -13.5 |
| Comprehensive | 2015 | 33% (425/1,275) | 48% (749/1,574) | -14.3 |
| Comprehensive | 2016 | 40% (523/1,303) | 65% (1,058/1,621) | -25.1 |
| Comprehensive | 2017 | 44% (538/1,233) | 68% (1,101/1,631) | -23.9 |
| Comprehensive | 2018 | 56% (700/1,254) | 77% (1,251/1,620) | -21.4 |
| Comprehensive | 2020 | 63% (725/1,150) | 83% (1,258/1,508) | -20.4 |
| Certified EHR (CEHRT) | 2009 | 67% (669/1,003) | 67% (814/1,217) | -0.2 |
| Certified EHR (CEHRT) | 2011 | 80% (886/1,109) | 84% (1,092/1,299) | -4.2 |
| Certified EHR (CEHRT) | 2012 | 94% (1,131/1,203) | 95% (1,366/1,445) | -0.5 |
| Certified EHR (CEHRT) | 2013 | 98% (1,155/1,182) | 98% (1,408/1,431) | -0.7 |
| Certified EHR (CEHRT) | 2014 | 98% (1,170/1,192) | 98% (1,503/1,530) | -0.1 |
| Certified EHR (CEHRT) | 2015 | 99% (1,223/1,234) | 99% (1,519/1,537) | 0.3 |
| Certified EHR (CEHRT) | 2016 | 98% (1,248/1,271) | 99% (1,579/1,596) | -0.7 |
| Certified EHR (CEHRT) | 2017 | 99% (1,164/1,181) | 99% (1,587/1,598) | -0.8 |
| Certified EHR (CEHRT) | 2018 | 97% (1,185/1,221) | 99% (1,575/1,595) | -1.7 |
| Certified EHR (CEHRT) | 2020 | 99% (1,095/1,108) | 99% (1,474/1,483) | -0.6 |
| Certified EHR (CEHRT) | 2021 | 99% (950/963) | 100% (1,293/1,295) | -1.2 |
| Certified EHR (CEHRT) | 2022 | 99% (1,113/1,123) | 100% (1,316/1,317) | -0.8 |
| Certified EHR (CEHRT) | 2023 | 99% (1,066/1,073) | 100% (1,244/1,248) | -0.3 |

For each core EHR capability and year, the table reports adoption percentages for rural and urban hospitals, the underlying counts (n/N), and the rural minus urban percentage‑point gap. Positive values indicate higher adoption in rural hospitals; negative values indicate higher adoption in urban hospitals.

**Supplemental Table 5. Advanced EHR use over time, 2012-2023**

| **Measure** | **Year** | **All hospitals** | **Urban hospitals** | **Rural hospitals** | **Gap (Rural - Urban, percentage points)** |
| --- | --- | --- | --- | --- | --- |
| Portal: patients can view clinical notes | 2021 | 89% (2,051/2,314) | 92% (1,223/1,324) | 84% (828/ 990) | -8.7 |
| Portal: patients can view clinical notes | 2022 | 94% (2,389/2,529) | 97% (1,308/1,345) | 91% (1,081/1,184) | -5.9 |
| Portal: patients can view clinical notes | 2023 | 95% (2,411/2,530) | 97% (1,343/1,384) | 93% (1,068/1,146) | -3.8 |
| Portal: patients can submit PGD | 2021 | 61% (1,384/2,280) | 61% (800/1,306) | 60% (584/974) | -1.3 |
| Portal: patients can submit PGD | 2022 | 63% (1,561/2,485) | 65% (867/1,330) | 60% (694/1,155) | -5.1 |
| Portal: patients can submit PGD | 2023 | 65% (1,635/2,508) | 67% (926/1,374) | 63% (709/1,134) | -4.9 |
| Portal: app access via API | 2021 | 76% (1,737/2,291) | 77% (1,007/1,310) | 74% (730/981) | -2.5 |
| Portal: app access via API | 2022 | 85% (2,125/2,502) | 89% (1,185/1,331) | 80% (940/1,171) | -8.8 |
| Portal: app access via API | 2023 | 86% (2,181/2,527) | 91% (1,261/1,384) | 80% (920/1,143) | -10.6 |
| Portal: FHIR-based app access | 2021 | 63% (1,430/2,278) | 67% (877/1,300) | 57% (553/ 978) | -10.9 |
| Portal: FHIR-based app access | 2022 | 73% (1,801/2,473) | 79% (1,041/1,317) | 66% (760/1,156) | -13.3 |
| Portal: FHIR-based app access | 2023 | 74% (1,862/2,521) | 77% (1,068/1,380) | 70% (794/1,141) | -7.8 |
| Portal: secure messaging with providers | 2021 | 91% (2,114/2,317) | 93% (1,237/1,323) | 88% (877/994) | -5.3 |
| Portal: secure messaging with providers | 2022 | 94% (2,376/2,523) | 96% (1,289/1,340) | 92% (1,087/1,183) | -4.3 |
| Portal: secure messaging with providers | 2023 | 94% (2,368/2,528) | 95% (1,313/1,383) | 92% (1,055/1,145) | -2.8 |
| Interoperability: queries external records | 2012 | 40% (1,145/2,867) | 46% (718/1,548) | 32% (427/1,319) | -14 |
| Interoperability: queries external records | 2013 | 54% (1,436/2,677) | 61% (892/1,458) | 45% (544/1,219) | -16.6 |
| Interoperability: queries external records | 2014 | 49% (1,331/2,729) | 58% (880/1,527) | 38% (451/1,202) | -20.1 |
| Interoperability: queries external records | 2015 | 55% (1,529/2,779) | 66% (1,014/1,542) | 42% (515/1,237) | -24.1 |
| Interoperability: queries external records | 2016 | 58% (1,677/2,869) | 70% (1,111/1,595) | 44% (566/1,274) | -25.2 |
| Interoperability: queries external records | 2017 | 66% (1,813/2,743) | 75% (1,186/1,586) | 54% (627/1,157) | -20.6 |
| Interoperability: queries external records | 2018 | 71% (1,938/2,734) | 80% (1,236/1,544) | 59% (702/1,190) | -21.1 |
| Interoperability: queries external records | 2020 | 78% (2,062/2,631) | 88% (1,315/1,499) | 66% (747/1,132) | -21.7 |
| Interoperability: queries external records | 2021 | 86% (1,988/2,319) | 92% (1,221/1,322) | 77% (767/997) | -15.4 |
| Interoperability: queries external records | 2022 | 89% (2,224/2,509) | 94% (1,259/1,340) | 83% (965/1,169) | -11.4 |
| Interoperability: queries external records | 2023 | 89% (2,210/2,495) | 94% (1,283/1,370) | 82% (927/1,125) | -11.2 |
| Interoperability: uses outside info often/sometimes | 2015 | 52% (1,495/2,849) | 63% (987/1,574) | 40% (508/1,275) | -22.9 |
| Interoperability: uses outside info often/sometimes | 2016 | 55% (1,616/2,924) | 64% (1,043/1,621) | 44% (573/1,303) | -20.4 |
| Interoperability: uses outside info often/sometimes | 2017 | 57% (1,625/2,864) | 65% (1,068/1,631) | 45% (557/1,233) | -20.3 |
| Interoperability: uses outside info often/sometimes | 2018 | 63% (1,821/2,874) | 71% (1,154/1,620) | 53% (667/1,254) | -18 |
| Interoperability: uses outside info often/sometimes | 2020 | 67% (1,784/2,658) | 76% (1,150/1,508) | 55% (634/1,150) | -21.1 |
| Interoperability: uses outside info often/sometimes | 2021 | 74% (1,760/2,363) | 82% (1,097/1,336) | 65% (663/1,027) | -17.6 |
| Interoperability: uses outside info often/sometimes | 2022 | 80% (2,034/2,548) | 87% (1,177/1,351) | 72% (857/1,197) | -15.5 |
| Interoperability: uses outside info often/sometimes | 2023 | 80% (2,039/2,545) | 87% (1,207/1,387) | 72% (832/1,158) | -15.2 |
| Interoperability: outside info available electronically | 2014 | 42% (1,136/2,682) | 48% (722/1,499) | 35% (414/1,183) | -13.2 |
| Interoperability: outside info available electronically | 2015 | 48% (1,337/2,796) | 56% (875/1,552) | 37% (462/1,244) | -19.2 |
| Interoperability: outside info available electronically | 2016 | 51% (1,460/2,872) | 59% (946/1,599) | 40% (514/1,273) | -18.8 |
| Interoperability: outside info available electronically | 2017 | 55% (1,523/2,773) | 64% (1,014/1,589) | 43% (509/1,184) | -20.8 |
| Interoperability: outside info available electronically | 2018 | 59% (1,676/2,837) | 68% (1,085/1,607) | 48% (591/1,230) | -19.5 |
| Interoperability: outside info available electronically | 2020 | 58% (1,504/2,611) | 68% (1,015/1,496) | 44% (489/1,115) | -24 |
| Interoperability: outside info available electronically | 2021 | 69% (1,562/2,272) | 77% (1,005/1,303) | 57% (557/969) | -19.6 |
| Interoperability: outside info available electronically | 2022 | 73% (1,797/2,475) | 79% (1,059/1,333) | 65% (738/1,142) | -14.8 |
| Interoperability: outside info available electronically | 2023 | 77% (1,897/2,461) | 85% (1,149/1,356) | 68% (748/1,105) | -17 |
| Interoperability: integrates received summaries | 2014 | 41% (1,118/2,755) | 45% (692/1,538) | 35% (426/1,217) | -10 |
| Interoperability: integrates received summaries | 2015 | 39% (1,108/2,849) | 44% (698/1,574) | 32% (410/1,275) | -12.2 |
| Interoperability: integrates received summaries | 2016 | 42% (1,232/2,924) | 49% (789/1,621) | 34% (443/1,303) | -14.7 |
| Interoperability: integrates received summaries | 2017 | 54% (1,555/2,864) | 61% (987/1,631) | 46% (568/1,233) | -14.4 |
| Interoperability: integrates received summaries | 2018 | 64% (1,831/2,874) | 70% (1,142/1,620) | 55% (689/1,254) | -15.5 |
| Interoperability: integrates received summaries | 2020 | 73% (1,949/2,658) | 80% (1,207/1,508) | 65% (742/1,150) | -15.5 |
| Interoperability: integrates received summaries | 2021 | 77% (1,817/2,363) | 83% (1,115/1,336) | 68% (702/1,027) | -15.1 |
| Interoperability: integrates received summaries | 2022 | 81% (2,066/2,548) | 87% (1,178/1,351) | 74% (888/1,197) | -13 |
| Interoperability: integrates received summaries | 2023 | 81% (2,060/2,545) | 86% (1,195/1,387) | 75% (865/1,158) | -11.5 |
| Interoperability: Rx benefit info integrated | 2022 | 65% (1,644/2,548) | 70% (941/1,351) | 59% (703/1,197) | -10.9 |
| Interoperability: Rx benefit info integrated | 2023 | 72% (1,834/2,545) | 76% (1,052/1,387) | 68% (782/1,158) | -8.3 |
| Public health: electronic case reporting engaged | 2021 | 51% (1,214/2,363) | 55% (738/1,336) | 46% (476/1,027) | -8.9 |
| Public health: electronic case reporting engaged | 2022 | 79% (2,014/2,548) | 83% (1,127/1,351) | 74% (887/1,197) | -9.3 |
| Public health: immunization registry engaged | 2021 | 91% (2,145/2,363) | 92% (1,231/1,336) | 89% (914/1,027) | -3.1 |
| Public health: immunization registry engaged | 2022 | 94% (2,398/2,548) | 96% (1,303/1,351) | 91% (1,095/1,197) | -5 |
| Public health: ELR engaged | 2021 | 86% (2,025/2,363) | 88% (1,170/1,336) | 83% (855/1,027) | -4.3 |
| Public health: ELR engaged | 2022 | 90% (2,292/2,548) | 93% (1,259/1,351) | 86% (1,033/1,197) | -6.9 |
| SDOH: collects social needs data | 2022 | 85% (2,159/2,548) | 92% (1,242/1,351) | 77% (917/1,197) | -15.3 |
| SDOH: collects social needs data | 2023 | 89% (2,273/2,545) | 96% (1,330/1,387) | 81% (943/1,158) | -14.5 |
| SDOH: used for clinical decision making | 2022 | 82% (1,737/2,129) | 84% (1,038/1,229) | 78% (699/ 900) | -6.8 |
| SDOH: used for clinical decision making | 2023 | 82% (1,848/2,251) | 85% (1,127/1,323) | 78% (721/ 928) | -7.5 |
| SDOH: used for analytics/population health | 2022 | 62% (1,317/2,129) | 65% (793/1,229) | 58% (524/ 900) | -6.3 |
| SDOH: used for analytics/population health | 2023 | 70% (1,575/2,251) | 72% (950/1,323) | 67% (625/ 928) | -4.5 |

Year-specific adoption of advanced EHR use measures (portal capabilities, interoperability, public health reporting, and SDOH functions) among all analytic hospitals combined. Cells show the percentage with the capability, the percentage (n/N), and the rural minus urban percentage-point gaps for each advanced EHR use measure and year.

**Supplemental Table 6. Tests for linear trends in core EHR adoption, 2008-2023**

| **Stratum** | **Measure** | **χ² statistic** | **Degrees of Freedom** | ***p* value** |
| --- | --- | --- | --- | --- |
| Overall | Basic - with notes | 12499.59 | 1 | <0.001 |
| Rural | Basic - with notes | 5517.14 | 1 | <0.001 |
| Urban | Basic - with notes | 7026.02 | 1 | <0.001 |
| Overall | Basic - without notes | 11886.13 | 1 | <0.001 |
| Rural | Basic - without notes | 5339.5 | 1 | <0.001 |
| Urban | Basic - without notes | 6632.57 | 1 | <0.001 |
| Overall | Comprehensive | 9862.02 | 1 | <0.001 |
| Rural | Comprehensive | 3573.58 | 1 | <0.001 |
| Urban | Comprehensive | 6367.75 | 1 | <0.001 |
| Overall | Certified EHR (CEHRT) | 2508.38 | 1 | <0.001 |
| Rural | Certified EHR (CEHRT) | 1052.99 | 1 | <0.001 |
| Urban | Certified EHR (CEHRT) | 1468.14 | 1 | <0.001 |

Cochran-Armitage tests for trend in proportions over survey year. All tests have 1 degree of freedom.

**Supplemental Table 7. Tests for linear trends in advanced EHR use, 2012-2023**

| **Stratum** | **Measure** | **χ² statistic** | ***p* value** |
| --- | --- | --- | --- |
| Overall | Portal: patients can view clinical notes | 79.73 | <0.001 |
| Rural | Portal: patients can view clinical notes | 50.83 | <0.001 |
| Urban | Portal: patients can view clinical notes | 34.35 | <0.001 |
| Overall | Portal: patients can submit PGD | 10.35 | 0.001 |
| Rural | Portal: patients can submit PGD | 1.52 | 0.218 |
| Urban | Portal: patients can submit PGD | 10.99 | <0.001 |
| Overall | Portal: app access via API | 89.62 | <0.001 |
| Rural | Portal: app access via API | 11.03 | <0.001 |
| Urban | Portal: app access via API | 110.57 | <0.001 |
| Overall | Portal: FHIR‑based app access | 68.18 | <0.001 |
| Rural | Portal: FHIR‑based app access | 38.26 | <0.001 |
| Urban | Portal: FHIR‑based app access | 34.03 | <0.001 |
| Overall | Portal: secure messaging with providers | 10.64 | 0.001 |
| Rural | Portal: secure messaging with providers | 9.41 | 0.002 |
| Urban | Portal: secure messaging with providers | 2.77 | 0.096 |
| Overall | Interoperability: queries external records | 3278.15 | <0.001 |
| Rural | Interoperability: queries external records | 1496.56 | <0.001 |
| Urban | Interoperability: queries external records | 1907.3 | <0.001 |
| Overall | Interoperability: uses outside info often/sometimes | 9146.56 | <0.001 |
| Rural | Interoperability: uses outside info often/sometimes | 3493.49 | <0.001 |
| Urban | Interoperability: uses outside info often/sometimes | 5875.21 | <0.001 |
| Overall | Interoperability: outside info available electronically | 1091.79 | <0.001 |
| Rural | Interoperability: outside info available electronically | 461.8 | <0.001 |
| Urban | Interoperability: outside info available electronically | 681.2 | <0.001 |
| Overall | Interoperability: integrates received summaries | 8334.83 | <0.001 |
| Rural | Interoperability: integrates received summaries | 3307.74 | <0.001 |
| Urban | Interoperability: integrates received summaries | 5159.58 | <0.001 |
| Overall | Interoperability: Rx benefit info integrated | 8960.31 | <0.001 |
| Rural | Interoperability: Rx benefit info integrated | 3750.49 | <0.001 |
| Urban | Interoperability: Rx benefit info integrated | 5218.81 | <0.001 |
| Overall | Public health: electronic case reporting engaged | 5682.67 | <0.001 |
| Rural | Public health: electronic case reporting engaged | 2381.32 | <0.001 |
| Urban | Public health: electronic case reporting engaged | 3307.01 | <0.001 |
| Overall | Public health: immunization registry engaged | 8049.26 | <0.001 |
| Rural | Public health: immunization registry engaged | 3535.24 | <0.001 |
| Urban | Public health: immunization registry engaged | 4515.95 | <0.001 |
| Overall | Public health: ELR engaged | 7592.38 | <0.001 |
| Rural | Public health: ELR engaged | 3290.58 | <0.001 |
| Urban | Public health: ELR engaged | 4305 | <0.001 |
| Overall | SDOH: collects social needs data | 11779.54 | <0.001 |
| Rural | SDOH: collects social needs data | 4814.56 | <0.001 |
| Urban | SDOH: collects social needs data | 6984.33 | <0.001 |
| Overall | SDOH: used for clinical decision making | - | - |
| Rural | SDOH: used for clinical decision making | - | - |
| Urban | SDOH: used for clinical decision making | - | - |
| Overall | SDOH: used for analytics/population health | - | - |
| Rural | SDOH: used for analytics/population health | - | - |
| Urban | SDOH: used for analytics/population health | - | - |

Cochran-Armitage tests for trend in proportions over survey year. All tests have 1 degree of freedom.

**Supplemental Table 8. Logistic regression for core functionality, 2008-2023**

| **Measure** | **Model** | **Term** | **Odds ratio (95% CI)** |
| --- | --- | --- | --- |
| Basic - without notes | Adjusted | Year | 1.72 (1.68–1.75) |
| Basic - without notes | Adjusted | Rural (reference: urban) | 0.92 (0.90–0.95) |
| Basic - without notes | Unadjusted | Year | 1.69 (1.65–1.73) |
| Basic - without notes | Unadjusted | Rural (reference: urban) | 0.92 (0.90–0.95) |
| Basic - with notes | Adjusted | Year | 1.68 (1.65–1.71) |
| Basic - with notes | Adjusted | Rural (reference: urban) | 0.94 (0.92–0.97) |
| Basic - with notes | Unadjusted | Year | 1.66 (1.63–1.69) |
| Basic - with notes | Unadjusted | Rural (reference: urban) | 0.94 (0.92–0.97) |
| Comprehensive | Adjusted | Year | 1.58 (1.56–1.61) |
| Comprehensive | Adjusted | Rural (reference: urban) | 0.93 (0.91–0.95) |
| Comprehensive | Unadjusted | Year | 1.56 (1.54–1.59) |
| Comprehensive | Unadjusted | Rural (reference: urban) | 0.94 (0.91–0.96) |
| Certified EHR (CEHRT) | Adjusted | Year | 1.76 (1.67–1.85) |
| Certified EHR (CEHRT) | Adjusted | Rural (reference: urban) | 0.87 (0.80–0.93) |
| Certified EHR (CEHRT) | Unadjusted | Year | 1.73 (1.65–1.82) |
| Certified EHR (CEHRT) | Unadjusted | Rural (reference: urban) | 0.87 (0.81–0.94) |

Logistic regression estimates summarizing time trends and rural-urban differences in core EHR adoption across all hospitals.

**Supplemental Table 9. Core EHR adoption by Census division and rurality, 2008-2023**

| **Measure** | **Census division** | **Year** | **All hospitals** | **Urban hospitals** | **Rural hospitals** | **Gap (Rural - Urban, percentage points)** |
| --- | --- | --- | --- | --- | --- | --- |
| Basic - with notes | East North Central | 2008 | 10% (49/514) | 12% (34/272) | 6% (15/242) | -6.3 |
| Basic - with notes | East North Central | 2009 | 13% (71/560) | 15% (45/298) | 10% (26/262) | -5.2 |
| Basic - with notes | East North Central | 2010 | 18% (89/507) | 24% (65/269) | 10% (24/238) | -14.1 |
| Basic - with notes | East North Central | 2011 | 31% (142/460) | 35% (88/250) | 26% (54/210) | -9.5 |
| Basic - with notes | East North Central | 2012 | 55% (278/507) | 66% (187/284) | 41% (91/223) | -25 |
| Basic - with notes | East North Central | 2013 | 68% (330/485) | 76% (206/270) | 58% (124/215) | -18.6 |
| Basic - with notes | East North Central | 2014 | 78% (358/460) | 84% (217/259) | 70% (141/201) | -13.6 |
| Basic - with notes | East North Central | 2015 | 87% (414/478) | 92% (248/270) | 80% (166/208) | -12 |
| Basic - with notes | East North Central | 2016 | 91% (442/486) | 92% (248/269) | 89% (194/217) | -2.8 |
| Basic - with notes | East North Central | 2017 | 84% (394/468) | 91% (244/267) | 75% (150/201) | -16.8 |
| Basic - with notes | East North Central | 2018 | 93% (457/490) | 97% (281/289) | 88% (176/201) | -9.7 |
| Basic - with notes | East North Central | 2020 | 96% (435/455) | 98% (251/256) | 92% (184/199) | -5.6 |
| Basic - with notes | East South Central | 2008 | 7% (14/204) | 5% (4/88) | 9% (10/116) | 4.1 |
| Basic - with notes | East South Central | 2009 | 7% (18/268) | 5% (5/109) | 8% (13/159) | 3.6 |
| Basic - with notes | East South Central | 2010 | 11% (24/210) | 11% (10/91) | 12% (14/119) | 0.8 |
| Basic - with notes | East South Central | 2011 | 22% (41/190) | 21% (17/80) | 22% (24/110) | 0.6 |
| Basic - with notes | East South Central | 2012 | 34% (68/199) | 35% (28/81) | 34% (40/118) | -0.7 |
| Basic - with notes | East South Central | 2013 | 52% (93/179) | 54% (37/69) | 51% (56/110) | -2.7 |
| Basic - with notes | East South Central | 2014 | 72% (126/175) | 80% (63/79) | 66% (63/96) | -14.1 |
| Basic - with notes | East South Central | 2015 | 84% (137/164) | 87% (67/77) | 80% (70/87) | -6.6 |
| Basic - with notes | East South Central | 2016 | 84% (161/191) | 87% (80/92) | 82% (81/99) | -5.1 |
| Basic - with notes | East South Central | 2017 | 68% (128/188) | 71% (65/91) | 65% (63/97) | -6.5 |
| Basic - with notes | East South Central | 2018 | 90% (159/176) | 91% (82/90) | 90% (77/86) | -1.6 |
| Basic - with notes | East South Central | 2020 | 91% (167/184) | 95% (76/80) | 88% (91/104) | -7.5 |
| Basic - with notes | Middle Atlantic | 2008 | 11% (31/271) | 13% (27/207) | 6% (4/64) | -6.8 |
| Basic - with notes | Middle Atlantic | 2009 | 11% (33/298) | 15% (32/218) | 1% (1/80) | -13.4 |
| Basic - with notes | Middle Atlantic | 2010 | 15% (45/294) | 18% (39/219) | 8% (6/75) | -9.8 |
| Basic - with notes | Middle Atlantic | 2011 | 26% (71/268) | 28% (56/200) | 22% (15/68) | -5.9 |
| Basic - with notes | Middle Atlantic | 2012 | 45% (126/278) | 46% (97/211) | 43% (29/67) | -2.7 |
| Basic - with notes | Middle Atlantic | 2013 | 56% (156/277) | 56% (119/214) | 59% (37/63) | 3.1 |
| Basic - with notes | Middle Atlantic | 2014 | 73% (192/264) | 74% (150/203) | 69% (42/61) | -5 |
| Basic - with notes | Middle Atlantic | 2015 | 80% (212/264) | 80% (165/206) | 81% (47/58) | 0.9 |
| Basic - with notes | Middle Atlantic | 2016 | 88% (248/282) | 88% (190/217) | 89% (58/65) | 1.7 |
| Basic - with notes | Middle Atlantic | 2017 | 72% (199/278) | 75% (165/219) | 58% (34/59) | -17.7 |
| Basic - with notes | Middle Atlantic | 2018 | 86% (238/276) | 90% (190/210) | 73% (48/66) | -17.7 |
| Basic - with notes | Middle Atlantic | 2020 | 95% (227/240) | 95% (180/190) | 94% (47/50) | -0.7 |
| Basic - with notes | Mountain | 2008 | 9% (17/198) | 13% (10/77) | 6% (7/121) | -7.2 |
| Basic - with notes | Mountain | 2009 | 14% (33/229) | 20% (18/92) | 11% (15/137) | -8.6 |
| Basic - with notes | Mountain | 2010 | 18% (36/204) | 29% (22/77) | 11% (14/127) | -17.5 |
| Basic - with notes | Mountain | 2011 | 23% (43/187) | 30% (23/76) | 18% (20/111) | -12.2 |
| Basic - with notes | Mountain | 2012 | 44% (86/196) | 56% (45/80) | 35% (41/116) | -20.9 |
| Basic - with notes | Mountain | 2013 | 65% (136/209) | 76% (69/91) | 57% (67/118) | -19 |
| Basic - with notes | Mountain | 2014 | 77% (157/203) | 88% (81/92) | 68% (76/111) | -19.6 |
| Basic - with notes | Mountain | 2015 | 85% (176/207) | 89% (76/85) | 82% (100/122) | -7.4 |
| Basic - with notes | Mountain | 2016 | 88% (179/204) | 92% (89/97) | 84% (90/107) | -7.6 |
| Basic - with notes | Mountain | 2017 | 79% (171/217) | 93% (94/101) | 66% (77/116) | -26.7 |
| Basic - with notes | Mountain | 2018 | 92% (194/212) | 99% (98/99) | 85% (96/113) | -14 |
| Basic - with notes | Mountain | 2020 | 94% (198/210) | 100% (105/105) | 89% (93/105) | -11.4 |
| Basic - with notes | New England | 2008 | 10% (13/130) | 14% (10/73) | 5% (3/57) | -8.4 |
| Basic - with notes | New England | 2009 | 20% (28/139) | 23% (21/90) | 14% (7/49) | -9 |
| Basic - with notes | New England | 2010 | 20% (29/144) | 20% (18/89) | 20% (11/55) | -0.2 |
| Basic - with notes | New England | 2011 | 31% (36/115) | 35% (26/75) | 25% (10/40) | -9.7 |
| Basic - with notes | New England | 2012 | 46% (53/116) | 53% (39/73) | 33% (14/43) | -20.9 |
| Basic - with notes | New England | 2013 | 62% (65/105) | 66% (45/68) | 54% (20/37) | -12.1 |
| Basic - with notes | New England | 2014 | 79% (83/105) | 82% (58/71) | 74% (25/34) | -8.2 |
| Basic - with notes | New England | 2015 | 86% (90/105) | 88% (58/66) | 82% (32/39) | -5.8 |
| Basic - with notes | New England | 2016 | 92% (109/119) | 90% (65/72) | 94% (44/47) | 3.3 |
| Basic - with notes | New England | 2017 | 75% (86/114) | 82% (56/68) | 65% (30/46) | -17.1 |
| Basic - with notes | New England | 2018 | 90% (112/125) | 93% (70/75) | 84% (42/50) | -9.3 |
| Basic - with notes | New England | 2020 | 95% (93/98) | 95% (58/61) | 95% (35/37) | -0.5 |
| Basic - with notes | Pacific | 2008 | 9% (26/279) | 11% (22/194) | 5% (4/85) | -6.6 |
| Basic - with notes | Pacific | 2009 | 16% (51/317) | 19% (41/221) | 10% (10/96) | -8.1 |
| Basic - with notes | Pacific | 2010 | 21% (61/289) | 28% (56/201) | 6% (5/88) | -22.2 |
| Basic - with notes | Pacific | 2011 | 31% (78/255) | 36% (62/172) | 19% (16/83) | -16.8 |
| Basic - with notes | Pacific | 2012 | 50% (148/297) | 55% (114/206) | 37% (34/91) | -18 |
| Basic - with notes | Pacific | 2013 | 61% (157/256) | 64% (117/184) | 56% (40/72) | -8 |
| Basic - with notes | Pacific | 2014 | 74% (196/264) | 78% (145/187) | 66% (51/77) | -11.3 |
| Basic - with notes | Pacific | 2015 | 86% (271/314) | 88% (196/222) | 82% (75/92) | -6.8 |
| Basic - with notes | Pacific | 2016 | 90% (248/277) | 93% (176/190) | 83% (72/87) | -9.9 |
| Basic - with notes | Pacific | 2017 | 86% (244/285) | 89% (182/205) | 78% (62/80) | -11.3 |
| Basic - with notes | Pacific | 2018 | 89% (231/261) | 91% (169/185) | 82% (62/76) | -9.8 |
| Basic - with notes | Pacific | 2020 | 91% (203/224) | 94% (149/159) | 83% (54/65) | -10.6 |
| Basic - with notes | South Atlantic | 2008 | 10% (45/434) | 12% (36/309) | 7% (9/125) | -4.5 |
| Basic - with notes | South Atlantic | 2009 | 14% (61/424) | 16% (46/282) | 11% (15/142) | -5.7 |
| Basic - with notes | South Atlantic | 2010 | 17% (72/418) | 20% (55/276) | 12% (17/142) | -8 |
| Basic - with notes | South Atlantic | 2011 | 33% (123/368) | 38% (101/263) | 21% (22/105) | -17.5 |
| Basic - with notes | South Atlantic | 2012 | 47% (176/377) | 50% (130/260) | 39% (46/117) | -10.7 |
| Basic - with notes | South Atlantic | 2013 | 66% (225/341) | 70% (171/246) | 57% (54/95) | -12.7 |
| Basic - with notes | South Atlantic | 2014 | 83% (329/397) | 85% (252/296) | 76% (77/101) | -8.9 |
| Basic - with notes | South Atlantic | 2015 | 85% (362/425) | 87% (271/312) | 81% (91/113) | -6.3 |
| Basic - with notes | South Atlantic | 2016 | 89% (420/471) | 90% (309/342) | 86% (111/129) | -4.3 |
| Basic - with notes | South Atlantic | 2017 | 83% (388/465) | 87% (302/349) | 74% (86/116) | -12.4 |
| Basic - with notes | South Atlantic | 2018 | 93% (427/461) | 94% (316/336) | 89% (111/125) | -5.2 |
| Basic - with notes | South Atlantic | 2020 | 96% (431/449) | 97% (320/330) | 93% (111/119) | -3.7 |
| Basic - with notes | West North Central | 2008 | 8% (33/429) | 17% (17/100) | 5% (16/329) | -12.1 |
| Basic - with notes | West North Central | 2009 | 12% (63/539) | 23% (33/142) | 8% (30/397) | -15.7 |
| Basic - with notes | West North Central | 2010 | 17% (93/539) | 26% (36/140) | 14% (57/399) | -11.4 |
| Basic - with notes | West North Central | 2011 | 31% (163/528) | 50% (71/142) | 24% (92/386) | -26.2 |
| Basic - with notes | West North Central | 2012 | 46% (237/519) | 59% (82/140) | 41% (155/379) | -17.7 |
| Basic - with notes | West North Central | 2013 | 57% (284/502) | 72% (98/137) | 51% (186/365) | -20.6 |
| Basic - with notes | West North Central | 2014 | 73% (368/502) | 82% (111/135) | 70% (257/367) | -12.2 |
| Basic - with notes | West North Central | 2015 | 82% (436/532) | 90% (123/137) | 79% (313/395) | -10.5 |
| Basic - with notes | West North Central | 2016 | 87% (450/518) | 92% (126/137) | 85% (324/381) | -6.9 |
| Basic - with notes | West North Central | 2017 | 77% (379/495) | 92% (125/136) | 71% (254/359) | -21.2 |
| Basic - with notes | West North Central | 2018 | 87% (429/492) | 95% (127/134) | 84% (302/358) | -10.4 |
| Basic - with notes | West North Central | 2020 | 91% (382/421) | 98% (118/121) | 88% (264/300) | -9.5 |
| Basic - with notes | West South Central | 2008 | 10% (39/407) | 10% (23/220) | 9% (16/187) | -1.9 |
| Basic - with notes | West South Central | 2009 | 8% (38/462) | 11% (27/239) | 5% (11/223) | -6.4 |
| Basic - with notes | West South Central | 2010 | 10% (42/412) | 10% (22/220) | 10% (20/192) | 0.4 |
| Basic - with notes | West South Central | 2011 | 20% (65/322) | 22% (36/164) | 18% (29/158) | -3.6 |
| Basic - with notes | West South Central | 2012 | 36% (157/434) | 39% (93/237) | 32% (64/197) | -6.8 |
| Basic - with notes | West South Central | 2013 | 54% (201/369) | 56% (112/200) | 53% (89/169) | -3.3 |
| Basic - with notes | West South Central | 2014 | 72% (279/385) | 75% (163/216) | 69% (116/169) | -6.8 |
| Basic - with notes | West South Central | 2015 | 81% (293/360) | 83% (166/199) | 79% (127/161) | -4.5 |
| Basic - with notes | West South Central | 2016 | 86% (324/376) | 89% (183/205) | 82% (141/171) | -6.8 |
| Basic - with notes | West South Central | 2017 | 71% (252/354) | 77% (151/195) | 64% (101/159) | -13.9 |
| Basic - with notes | West South Central | 2018 | 87% (331/381) | 93% (188/202) | 80% (143/179) | -13.2 |
| Basic - with notes | West South Central | 2020 | 89% (335/377) | 94% (194/206) | 82% (141/171) | -11.7 |
| Basic - without notes | East North Central | 2008 | 14% (73/514) | 19% (51/272) | 9% (22/242) | -9.7 |
| Basic - without notes | East North Central | 2009 | 18% (99/560) | 21% (64/298) | 13% (35/262) | -8.1 |
| Basic - without notes | East North Central | 2010 | 24% (121/507) | 32% (87/269) | 14% (34/238) | -18.1 |
| Basic - without notes | East North Central | 2011 | 40% (182/460) | 47% (117/250) | 31% (65/210) | -15.8 |
| Basic - without notes | East North Central | 2012 | 67% (340/507) | 78% (221/284) | 53% (119/223) | -24.5 |
| Basic - without notes | East North Central | 2013 | 79% (385/485) | 88% (237/270) | 69% (148/215) | -18.9 |
| Basic - without notes | East North Central | 2014 | 88% (403/460) | 92% (237/259) | 83% (166/201) | -8.9 |
| Basic - without notes | East North Central | 2015 | 90% (432/478) | 96% (260/270) | 83% (172/208) | -13.6 |
| Basic - without notes | East North Central | 2016 | 93% (451/486) | 94% (253/269) | 91% (198/217) | -2.8 |
| Basic - without notes | East North Central | 2017 | 88% (411/468) | 94% (250/267) | 80% (161/201) | -13.5 |
| Basic - without notes | East North Central | 2018 | 95% (466/490) | 98% (284/289) | 91% (182/201) | -7.7 |
| Basic - without notes | East North Central | 2020 | 96% (438/455) | 98% (251/256) | 94% (187/199) | -4.1 |
| Basic - without notes | East South Central | 2008 | 9% (19/204) | 7% (6/88) | 11% (13/116) | 4.4 |
| Basic - without notes | East South Central | 2009 | 9% (24/268) | 8% (9/109) | 9% (15/159) | 1.2 |
| Basic - without notes | East South Central | 2010 | 14% (30/210) | 14% (13/91) | 14% (17/119) | 0 |
| Basic - without notes | East South Central | 2011 | 30% (57/190) | 32% (26/80) | 28% (31/110) | -4.3 |
| Basic - without notes | East South Central | 2012 | 53% (106/199) | 56% (45/81) | 52% (61/118) | -3.9 |
| Basic - without notes | East South Central | 2013 | 72% (129/179) | 80% (55/69) | 67% (74/110) | -12.4 |
| Basic - without notes | East South Central | 2014 | 81% (141/175) | 89% (70/79) | 74% (71/96) | -14.6 |
| Basic - without notes | East South Central | 2015 | 87% (143/164) | 90% (69/77) | 85% (74/87) | -4.6 |
| Basic - without notes | East South Central | 2016 | 89% (170/191) | 92% (85/92) | 86% (85/99) | -6.5 |
| Basic - without notes | East South Central | 2017 | 74% (139/188) | 80% (73/91) | 68% (66/97) | -12.2 |
| Basic - without notes | East South Central | 2018 | 90% (159/176) | 91% (82/90) | 90% (77/86) | -1.6 |
| Basic - without notes | East South Central | 2020 | 93% (171/184) | 98% (78/80) | 89% (93/104) | -8.1 |
| Basic - without notes | Middle Atlantic | 2008 | 15% (40/271) | 17% (35/207) | 8% (5/64) | -9.1 |
| Basic - without notes | Middle Atlantic | 2009 | 20% (59/298) | 24% (52/218) | 9% (7/80) | -15.1 |
| Basic - without notes | Middle Atlantic | 2010 | 20% (59/294) | 24% (52/219) | 9% (7/75) | -14.4 |
| Basic - without notes | Middle Atlantic | 2011 | 37% (99/268) | 39% (78/200) | 31% (21/68) | -8.1 |
| Basic - without notes | Middle Atlantic | 2012 | 64% (178/278) | 64% (135/211) | 64% (43/67) | 0.2 |
| Basic - without notes | Middle Atlantic | 2013 | 75% (208/277) | 77% (164/214) | 70% (44/63) | -6.8 |
| Basic - without notes | Middle Atlantic | 2014 | 88% (232/264) | 92% (186/203) | 75% (46/61) | -16.2 |
| Basic - without notes | Middle Atlantic | 2015 | 92% (244/264) | 93% (192/206) | 90% (52/58) | -3.5 |
| Basic - without notes | Middle Atlantic | 2016 | 93% (262/282) | 93% (202/217) | 92% (60/65) | -0.8 |
| Basic - without notes | Middle Atlantic | 2017 | 79% (219/278) | 82% (179/219) | 68% (40/59) | -13.9 |
| Basic - without notes | Middle Atlantic | 2018 | 92% (253/276) | 95% (199/210) | 82% (54/66) | -12.9 |
| Basic - without notes | Middle Atlantic | 2020 | 96% (231/240) | 97% (184/190) | 94% (47/50) | -2.8 |
| Basic - without notes | Mountain | 2008 | 11% (22/198) | 17% (13/77) | 7% (9/121) | -9.4 |
| Basic - without notes | Mountain | 2009 | 17% (38/229) | 25% (23/92) | 11% (15/137) | -14.1 |
| Basic - without notes | Mountain | 2010 | 21% (43/204) | 31% (24/77) | 15% (19/127) | -16.2 |
| Basic - without notes | Mountain | 2011 | 25% (47/187) | 34% (26/76) | 19% (21/111) | -15.3 |
| Basic - without notes | Mountain | 2012 | 51% (100/196) | 61% (49/80) | 44% (51/116) | -17.3 |
| Basic - without notes | Mountain | 2013 | 70% (147/209) | 84% (76/91) | 60% (71/118) | -23.3 |
| Basic - without notes | Mountain | 2014 | 82% (166/203) | 95% (87/92) | 71% (79/111) | -23.4 |
| Basic - without notes | Mountain | 2015 | 86% (179/207) | 93% (79/85) | 82% (100/122) | -11 |
| Basic - without notes | Mountain | 2016 | 88% (179/204) | 92% (89/97) | 84% (90/107) | -7.6 |
| Basic - without notes | Mountain | 2017 | 81% (175/217) | 93% (94/101) | 70% (81/116) | -23.2 |
| Basic - without notes | Mountain | 2018 | 92% (194/212) | 99% (98/99) | 85% (96/113) | -14 |
| Basic - without notes | Mountain | 2020 | 94% (198/210) | 100% (105/105) | 89% (93/105) | -11.4 |
| Basic - without notes | New England | 2008 | 21% (27/130) | 27% (20/73) | 12% (7/57) | -15.1 |
| Basic - without notes | New England | 2009 | 27% (37/139) | 32% (29/90) | 16% (8/49) | -15.9 |
| Basic - without notes | New England | 2010 | 26% (37/144) | 29% (26/89) | 20% (11/55) | -9.2 |
| Basic - without notes | New England | 2011 | 50% (58/115) | 56% (42/75) | 40% (16/40) | -16 |
| Basic - without notes | New England | 2012 | 71% (82/116) | 77% (56/73) | 60% (26/43) | -16.2 |
| Basic - without notes | New England | 2013 | 79% (83/105) | 78% (53/68) | 81% (30/37) | 3.1 |
| Basic - without notes | New England | 2014 | 90% (95/105) | 90% (64/71) | 91% (31/34) | 1 |
| Basic - without notes | New England | 2015 | 91% (96/105) | 94% (62/66) | 87% (34/39) | -6.8 |
| Basic - without notes | New England | 2016 | 92% (110/119) | 92% (66/72) | 94% (44/47) | 2 |
| Basic - without notes | New England | 2017 | 82% (93/114) | 85% (58/68) | 76% (35/46) | -9.2 |
| Basic - without notes | New England | 2018 | 91% (114/125) | 96% (72/75) | 84% (42/50) | -12 |
| Basic - without notes | New England | 2020 | 96% (94/98) | 97% (59/61) | 95% (35/37) | -2.1 |
| Basic - without notes | Pacific | 2008 | 13% (37/279) | 16% (32/194) | 6% (5/85) | -10.6 |
| Basic - without notes | Pacific | 2009 | 21% (65/317) | 24% (54/221) | 11% (11/96) | -13 |
| Basic - without notes | Pacific | 2010 | 22% (63/289) | 29% (58/201) | 6% (5/88) | -23.2 |
| Basic - without notes | Pacific | 2011 | 33% (83/255) | 38% (66/172) | 20% (17/83) | -17.9 |
| Basic - without notes | Pacific | 2012 | 57% (169/297) | 63% (130/206) | 43% (39/91) | -20.2 |
| Basic - without notes | Pacific | 2013 | 67% (171/256) | 69% (127/184) | 61% (44/72) | -7.9 |
| Basic - without notes | Pacific | 2014 | 79% (208/264) | 82% (153/187) | 71% (55/77) | -10.4 |
| Basic - without notes | Pacific | 2015 | 90% (283/314) | 92% (205/222) | 85% (78/92) | -7.6 |
| Basic - without notes | Pacific | 2016 | 92% (255/277) | 95% (180/190) | 86% (75/87) | -8.5 |
| Basic - without notes | Pacific | 2017 | 89% (255/285) | 94% (192/205) | 79% (63/80) | -14.9 |
| Basic - without notes | Pacific | 2018 | 90% (236/261) | 93% (172/185) | 84% (64/76) | -8.8 |
| Basic - without notes | Pacific | 2020 | 91% (203/224) | 94% (149/159) | 83% (54/65) | -10.6 |
| Basic - without notes | South Atlantic | 2008 | 16% (70/434) | 19% (58/309) | 10% (12/125) | -9.2 |
| Basic - without notes | South Atlantic | 2009 | 18% (78/424) | 21% (59/282) | 13% (19/142) | -7.5 |
| Basic - without notes | South Atlantic | 2010 | 23% (95/418) | 27% (74/276) | 15% (21/142) | -12 |
| Basic - without notes | South Atlantic | 2011 | 45% (164/368) | 48% (125/263) | 37% (39/105) | -10.4 |
| Basic - without notes | South Atlantic | 2012 | 61% (229/377) | 63% (164/260) | 56% (65/117) | -7.5 |
| Basic - without notes | South Atlantic | 2013 | 75% (257/341) | 80% (196/246) | 64% (61/95) | -15.5 |
| Basic - without notes | South Atlantic | 2014 | 90% (357/397) | 92% (271/296) | 85% (86/101) | -6.4 |
| Basic - without notes | South Atlantic | 2015 | 92% (389/425) | 93% (290/312) | 88% (99/113) | -5.3 |
| Basic - without notes | South Atlantic | 2016 | 92% (435/471) | 94% (322/342) | 88% (113/129) | -6.6 |
| Basic - without notes | South Atlantic | 2017 | 85% (396/465) | 88% (308/349) | 76% (88/116) | -12.4 |
| Basic - without notes | South Atlantic | 2018 | 94% (432/461) | 96% (321/336) | 89% (111/125) | -6.7 |
| Basic - without notes | South Atlantic | 2020 | 96% (432/449) | 97% (321/330) | 93% (111/119) | -4 |
| Basic - without notes | West North Central | 2008 | 9% (40/429) | 21% (21/100) | 6% (19/329) | -15.2 |
| Basic - without notes | West North Central | 2009 | 13% (72/539) | 27% (38/142) | 9% (34/397) | -18.2 |
| Basic - without notes | West North Central | 2010 | 19% (103/539) | 28% (39/140) | 16% (64/399) | -11.8 |
| Basic - without notes | West North Central | 2011 | 35% (185/528) | 55% (78/142) | 28% (107/386) | -27.2 |
| Basic - without notes | West North Central | 2012 | 53% (277/519) | 70% (98/140) | 47% (179/379) | -22.8 |
| Basic - without notes | West North Central | 2013 | 65% (324/502) | 84% (115/137) | 57% (209/365) | -26.7 |
| Basic - without notes | West North Central | 2014 | 79% (397/502) | 90% (122/135) | 75% (275/367) | -15.4 |
| Basic - without notes | West North Central | 2015 | 85% (451/532) | 96% (131/137) | 81% (320/395) | -14.6 |
| Basic - without notes | West North Central | 2016 | 89% (461/518) | 96% (132/137) | 86% (329/381) | -10 |
| Basic - without notes | West North Central | 2017 | 79% (389/495) | 94% (128/136) | 73% (261/359) | -21.4 |
| Basic - without notes | West North Central | 2018 | 88% (432/492) | 96% (128/134) | 85% (304/358) | -10.6 |
| Basic - without notes | West North Central | 2020 | 91% (384/421) | 98% (119/121) | 88% (265/300) | -10 |
| Basic - without notes | West South Central | 2008 | 13% (52/407) | 15% (32/220) | 11% (20/187) | -3.9 |
| Basic - without notes | West South Central | 2009 | 12% (56/462) | 17% (41/239) | 7% (15/223) | -10.4 |
| Basic - without notes | West South Central | 2010 | 12% (51/412) | 13% (29/220) | 11% (22/192) | -1.7 |
| Basic - without notes | West South Central | 2011 | 26% (84/322) | 30% (50/164) | 22% (34/158) | -9 |
| Basic - without notes | West South Central | 2012 | 43% (186/434) | 45% (106/237) | 41% (80/197) | -4.1 |
| Basic - without notes | West South Central | 2013 | 66% (245/369) | 67% (134/200) | 66% (111/169) | -1.3 |
| Basic - without notes | West South Central | 2014 | 79% (304/385) | 83% (180/216) | 73% (124/169) | -10 |
| Basic - without notes | West South Central | 2015 | 85% (306/360) | 87% (174/199) | 82% (132/161) | -5.4 |
| Basic - without notes | West South Central | 2016 | 88% (332/376) | 92% (189/205) | 84% (143/171) | -8.6 |
| Basic - without notes | West South Central | 2017 | 77% (273/354) | 83% (162/195) | 70% (111/159) | -13.3 |
| Basic - without notes | West South Central | 2018 | 88% (334/381) | 94% (189/202) | 81% (145/179) | -12.6 |
| Basic - without notes | West South Central | 2020 | 89% (337/377) | 94% (194/206) | 84% (143/171) | -10.5 |
| Certified EHR (CEHRT) | East North Central | 2009 | 69% (307/445) | 68% (162/240) | 71% (145/205) | 3.2 |
| Certified EHR (CEHRT) | East North Central | 2011 | 82% (351/429) | 85% (196/231) | 78% (155/198) | -6.6 |
| Certified EHR (CEHRT) | East North Central | 2012 | 95% (453/476) | 95% (257/270) | 95% (196/206) | 0 |
| Certified EHR (CEHRT) | East North Central | 2013 | 99% (471/474) | 99% (262/264) | 100% (209/210) | 0.3 |
| Certified EHR (CEHRT) | East North Central | 2014 | 99% (456/459) | 99% (257/259) | 100% (199/200) | 0.3 |
| Certified EHR (CEHRT) | East North Central | 2015 | 100% (468/469) | 100% (264/265) | 100% (204/204) | 0.4 |
| Certified EHR (CEHRT) | East North Central | 2016 | 100% (482/482) | 100% (266/266) | 100% (216/216) | 0 |
| Certified EHR (CEHRT) | East North Central | 2017 | 100% (457/459) | 100% (265/266) | 99% (192/193) | -0.1 |
| Certified EHR (CEHRT) | East North Central | 2018 | 98% (471/482) | 99% (280/284) | 96% (191/198) | -2.1 |
| Certified EHR (CEHRT) | East North Central | 2020 | 99% (441/444) | 100% (251/252) | 99% (190/192) | -0.6 |
| Certified EHR (CEHRT) | East North Central | 2021 | 100% (397/397) | 100% (217/217) | 100% (180/180) | 0 |
| Certified EHR (CEHRT) | East North Central | 2022 | 100% (415/415) | 100% (212/212) | 100% (203/203) | 0 |
| Certified EHR (CEHRT) | East North Central | 2023 | 100% (356/357) | 100% (186/186) | 99% (170/171) | -0.6 |
| Certified EHR (CEHRT) | East South Central | 2009 | 65% (112/173) | 56% (41/73) | 71% (71/100) | 14.8 |
| Certified EHR (CEHRT) | East South Central | 2011 | 81% (132/163) | 87% (58/67) | 77% (74/96) | -9.5 |
| Certified EHR (CEHRT) | East South Central | 2012 | 98% (182/185) | 99% (76/77) | 98% (106/108) | -0.6 |
| Certified EHR (CEHRT) | East South Central | 2013 | 98% (171/174) | 100% (68/68) | 97% (103/106) | -2.8 |
| Certified EHR (CEHRT) | East South Central | 2014 | 99% (168/170) | 100% (78/78) | 98% (90/92) | -2.2 |
| Certified EHR (CEHRT) | East South Central | 2015 | 100% (158/158) | 100% (76/76) | 100% (82/82) | 0 |
| Certified EHR (CEHRT) | East South Central | 2016 | 98% (186/189) | 99% (90/91) | 98% (96/98) | -0.9 |
| Certified EHR (CEHRT) | East South Central | 2017 | 98% (184/187) | 99% (90/91) | 98% (94/96) | -1 |
| Certified EHR (CEHRT) | East South Central | 2018 | 99% (168/170) | 99% (89/90) | 99% (79/80) | -0.1 |
| Certified EHR (CEHRT) | East South Central | 2020 | 100% (177/177) | 100% (79/79) | 100% (98/98) | 0 |
| Certified EHR (CEHRT) | East South Central | 2021 | 100% (128/128) | 100% (65/65) | 100% (63/63) | 0 |
| Certified EHR (CEHRT) | East South Central | 2022 | 98% (176/179) | 100% (76/76) | 97% (100/103) | -2.9 |
| Certified EHR (CEHRT) | East South Central | 2023 | 100% (120/120) | 100% (51/51) | 100% (69/69) | 0 |
| Certified EHR (CEHRT) | Middle Atlantic | 2009 | 68% (165/243) | 66% (123/186) | 74% (42/57) | 7.6 |
| Certified EHR (CEHRT) | Middle Atlantic | 2011 | 84% (210/250) | 83% (156/188) | 87% (54/62) | 4.1 |
| Certified EHR (CEHRT) | Middle Atlantic | 2012 | 97% (252/261) | 95% (187/196) | 100% (65/65) | 4.6 |
| Certified EHR (CEHRT) | Middle Atlantic | 2013 | 99% (262/264) | 99% (200/202) | 100% (62/62) | 1 |
| Certified EHR (CEHRT) | Middle Atlantic | 2014 | 98% (259/263) | 98% (198/202) | 100% (61/61) | 2 |
| Certified EHR (CEHRT) | Middle Atlantic | 2015 | 99% (253/256) | 99% (198/200) | 98% (55/56) | -0.8 |
| Certified EHR (CEHRT) | Middle Atlantic | 2016 | 99% (275/279) | 99% (211/214) | 98% (64/65) | -0.1 |
| Certified EHR (CEHRT) | Middle Atlantic | 2017 | 99% (266/270) | 98% (210/214) | 100% (56/56) | 1.9 |
| Certified EHR (CEHRT) | Middle Atlantic | 2018 | 98% (263/268) | 98% (202/206) | 98% (61/62) | 0.3 |
| Certified EHR (CEHRT) | Middle Atlantic | 2020 | 98% (229/234) | 98% (182/186) | 98% (47/48) | 0.1 |
| Certified EHR (CEHRT) | Middle Atlantic | 2021 | 99% (206/209) | 99% (155/156) | 96% (51/53) | -3.1 |
| Certified EHR (CEHRT) | Middle Atlantic | 2022 | 100% (232/232) | 100% (180/180) | 100% (52/52) | 0 |
| Certified EHR (CEHRT) | Middle Atlantic | 2023 | 100% (219/220) | 100% (161/161) | 98% (58/59) | -1.7 |
| Certified EHR (CEHRT) | Mountain | 2009 | 64% (105/163) | 70% (43/61) | 61% (62/102) | -9.7 |
| Certified EHR (CEHRT) | Mountain | 2011 | 76% (126/166) | 82% (58/71) | 72% (68/95) | -10.1 |
| Certified EHR (CEHRT) | Mountain | 2012 | 92% (161/175) | 91% (69/76) | 93% (92/99) | 2.1 |
| Certified EHR (CEHRT) | Mountain | 2013 | 98% (197/201) | 99% (89/90) | 97% (108/111) | -1.6 |
| Certified EHR (CEHRT) | Mountain | 2014 | 98% (196/199) | 99% (90/91) | 98% (106/108) | -0.8 |
| Certified EHR (CEHRT) | Mountain | 2015 | 96% (195/203) | 92% (78/85) | 99% (117/118) | 7.4 |
| Certified EHR (CEHRT) | Mountain | 2016 | 96% (190/198) | 96% (92/96) | 96% (98/102) | 0.2 |
| Certified EHR (CEHRT) | Mountain | 2017 | 98% (205/209) | 100% (96/96) | 96% (109/113) | -3.5 |
| Certified EHR (CEHRT) | Mountain | 2018 | 98% (204/209) | 100% (98/98) | 95% (106/111) | -4.5 |
| Certified EHR (CEHRT) | Mountain | 2020 | 100% (201/202) | 100% (101/101) | 99% (100/101) | -1 |
| Certified EHR (CEHRT) | Mountain | 2021 | 98% (179/183) | 100% (102/102) | 95% (77/81) | -4.9 |
| Certified EHR (CEHRT) | Mountain | 2022 | 100% (201/202) | 100% (104/104) | 99% (97/98) | -1 |
| Certified EHR (CEHRT) | Mountain | 2023 | 100% (215/215) | 100% (110/110) | 100% (105/105) | 0 |
| Certified EHR (CEHRT) | New England | 2009 | 77% (96/125) | 78% (64/82) | 74% (32/43) | -3.6 |
| Certified EHR (CEHRT) | New England | 2011 | 89% (100/112) | 89% (66/74) | 89% (34/38) | 0.3 |
| Certified EHR (CEHRT) | New England | 2012 | 95% (107/113) | 94% (67/71) | 95% (40/42) | 0.9 |
| Certified EHR (CEHRT) | New England | 2013 | 98% (103/105) | 97% (66/68) | 100% (37/37) | 2.9 |
| Certified EHR (CEHRT) | New England | 2014 | 98% (103/105) | 97% (69/71) | 100% (34/34) | 2.8 |
| Certified EHR (CEHRT) | New England | 2015 | 98% (100/102) | 98% (62/63) | 97% (38/39) | -1 |
| Certified EHR (CEHRT) | New England | 2016 | 99% (114/115) | 100% (69/69) | 98% (45/46) | -2.2 |
| Certified EHR (CEHRT) | New England | 2017 | 100% (113/113) | 100% (68/68) | 100% (45/45) | 0 |
| Certified EHR (CEHRT) | New England | 2018 | 100% (120/120) | 100% (72/72) | 100% (48/48) | 0 |
| Certified EHR (CEHRT) | New England | 2020 | 98% (96/98) | 100% (61/61) | 95% (35/37) | -5.4 |
| Certified EHR (CEHRT) | New England | 2021 | 99% (107/108) | 100% (64/64) | 98% (43/44) | -2.3 |
| Certified EHR (CEHRT) | New England | 2022 | 100% (90/90) | 100% (53/53) | 100% (37/37) | 0 |
| Certified EHR (CEHRT) | New England | 2023 | 100% (93/93) | 100% (55/55) | 100% (38/38) | 0 |
| Certified EHR (CEHRT) | Pacific | 2009 | 65% (149/229) | 67% (109/162) | 60% (40/67) | -7.6 |
| Certified EHR (CEHRT) | Pacific | 2011 | 78% (176/226) | 85% (131/154) | 62% (45/72) | -22.6 |
| Certified EHR (CEHRT) | Pacific | 2012 | 87% (217/249) | 89% (154/173) | 83% (63/76) | -6.1 |
| Certified EHR (CEHRT) | Pacific | 2013 | 96% (238/247) | 97% (170/176) | 96% (68/71) | -0.8 |
| Certified EHR (CEHRT) | Pacific | 2014 | 95% (248/260) | 96% (179/187) | 95% (69/73) | -1.2 |
| Certified EHR (CEHRT) | Pacific | 2015 | 99% (304/308) | 99% (216/219) | 99% (88/89) | 0.2 |
| Certified EHR (CEHRT) | Pacific | 2016 | 99% (268/271) | 99% (185/187) | 99% (83/84) | -0.1 |
| Certified EHR (CEHRT) | Pacific | 2017 | 100% (273/274) | 100% (199/199) | 99% (74/75) | -1.3 |
| Certified EHR (CEHRT) | Pacific | 2018 | 98% (253/257) | 98% (179/183) | 100% (74/74) | 2.2 |
| Certified EHR (CEHRT) | Pacific | 2020 | 100% (215/215) | 100% (154/154) | 100% (61/61) | 0 |
| Certified EHR (CEHRT) | Pacific | 2021 | 99% (179/180) | 100% (137/137) | 98% (42/43) | -2.3 |
| Certified EHR (CEHRT) | Pacific | 2022 | 100% (201/201) | 100% (147/147) | 100% (54/54) | 0 |
| Certified EHR (CEHRT) | Pacific | 2023 | 100% (249/249) | 100% (176/176) | 100% (73/73) | 0 |
| Certified EHR (CEHRT) | South Atlantic | 2009 | 73% (217/299) | 74% (147/200) | 71% (70/99) | -2.8 |
| Certified EHR (CEHRT) | South Atlantic | 2011 | 84% (277/331) | 82% (198/241) | 88% (79/90) | 5.6 |
| Certified EHR (CEHRT) | South Atlantic | 2012 | 96% (340/355) | 96% (236/245) | 95% (104/110) | -1.8 |
| Certified EHR (CEHRT) | South Atlantic | 2013 | 99% (327/331) | 99% (236/239) | 99% (91/92) | 0.2 |
| Certified EHR (CEHRT) | South Atlantic | 2014 | 99% (389/394) | 99% (291/293) | 97% (98/101) | -2.3 |
| Certified EHR (CEHRT) | South Atlantic | 2015 | 99% (412/415) | 99% (304/307) | 100% (108/108) | 1 |
| Certified EHR (CEHRT) | South Atlantic | 2016 | 100% (460/462) | 99% (333/335) | 100% (127/127) | 0.6 |
| Certified EHR (CEHRT) | South Atlantic | 2017 | 100% (453/454) | 100% (343/344) | 100% (110/110) | 0.3 |
| Certified EHR (CEHRT) | South Atlantic | 2018 | 99% (449/452) | 99% (329/331) | 99% (120/121) | -0.2 |
| Certified EHR (CEHRT) | South Atlantic | 2020 | 100% (442/442) | 100% (327/327) | 100% (115/115) | 0 |
| Certified EHR (CEHRT) | South Atlantic | 2021 | 100% (391/391) | 100% (287/287) | 100% (104/104) | 0 |
| Certified EHR (CEHRT) | South Atlantic | 2022 | 100% (407/408) | 100% (274/274) | 99% (133/134) | -0.7 |
| Certified EHR (CEHRT) | South Atlantic | 2023 | 100% (355/356) | 100% (247/248) | 100% (108/108) | 0.4 |
| Certified EHR (CEHRT) | West North Central | 2009 | 68% (155/228) | 65% (35/54) | 69% (120/174) | 4.2 |
| Certified EHR (CEHRT) | West North Central | 2011 | 82% (387/471) | 87% (117/135) | 80% (270/336) | -6.3 |
| Certified EHR (CEHRT) | West North Central | 2012 | 92% (434/471) | 92% (122/132) | 92% (312/339) | -0.4 |
| Certified EHR (CEHRT) | West North Central | 2013 | 97% (451/467) | 98% (131/133) | 96% (320/334) | -2.7 |
| Certified EHR (CEHRT) | West North Central | 2014 | 98% (485/494) | 99% (132/134) | 98% (353/360) | -0.5 |
| Certified EHR (CEHRT) | West North Central | 2015 | 99% (512/518) | 100% (134/134) | 98% (378/384) | -1.6 |
| Certified EHR (CEHRT) | West North Central | 2016 | 97% (490/504) | 97% (132/136) | 97% (358/368) | 0.2 |
| Certified EHR (CEHRT) | West North Central | 2017 | 99% (474/480) | 100% (134/134) | 98% (340/346) | -1.7 |
| Certified EHR (CEHRT) | West North Central | 2018 | 99% (475/482) | 100% (133/133) | 98% (342/349) | -2 |
| Certified EHR (CEHRT) | West North Central | 2020 | 100% (409/411) | 99% (120/121) | 100% (289/290) | 0.5 |
| Certified EHR (CEHRT) | West North Central | 2021 | 100% (377/378) | 99% (108/109) | 100% (269/269) | 0.9 |
| Certified EHR (CEHRT) | West North Central | 2022 | 100% (418/419) | 100% (116/116) | 100% (302/303) | -0.3 |
| Certified EHR (CEHRT) | West North Central | 2023 | 99% (418/421) | 99% (110/111) | 99% (308/310) | 0.3 |
| Certified EHR (CEHRT) | West South Central | 2009 | 56% (177/315) | 57% (90/159) | 56% (87/156) | -0.8 |
| Certified EHR (CEHRT) | West South Central | 2011 | 84% (219/260) | 81% (112/138) | 88% (107/122) | 6.5 |
| Certified EHR (CEHRT) | West South Central | 2012 | 97% (351/363) | 97% (198/205) | 97% (153/158) | 0.3 |
| Certified EHR (CEHRT) | West South Central | 2013 | 98% (343/350) | 97% (186/191) | 99% (157/159) | 1.4 |
| Certified EHR (CEHRT) | West South Central | 2014 | 98% (369/378) | 97% (209/215) | 98% (160/163) | 1 |
| Certified EHR (CEHRT) | West South Central | 2015 | 99% (340/342) | 99% (187/188) | 99% (153/154) | -0.1 |
| Certified EHR (CEHRT) | West South Central | 2016 | 99% (362/367) | 100% (201/202) | 98% (161/165) | -1.9 |
| Certified EHR (CEHRT) | West South Central | 2017 | 98% (326/333) | 98% (182/186) | 98% (144/147) | 0.1 |
| Certified EHR (CEHRT) | West South Central | 2018 | 95% (357/376) | 97% (193/198) | 92% (164/178) | -5.3 |
| Certified EHR (CEHRT) | West South Central | 2020 | 98% (359/368) | 99% (199/202) | 96% (160/166) | -2.1 |
| Certified EHR (CEHRT) | West South Central | 2021 | 98% (279/284) | 100% (158/158) | 96% (121/126) | -4 |
| Certified EHR (CEHRT) | West South Central | 2022 | 98% (289/294) | 99% (154/155) | 97% (135/139) | -2.2 |
| Certified EHR (CEHRT) | West South Central | 2023 | 98% (285/290) | 99% (148/150) | 98% (137/140) | -0.8 |
| Comprehensive | East North Central | 2008 | 2% (9/514) | 3% (8/272) | 0% (1/242) | -2.5 |
| Comprehensive | East North Central | 2009 | 3% (17/560) | 5% (14/298) | 1% (3/262) | -3.6 |
| Comprehensive | East North Central | 2010 | 5% (24/507) | 7% (18/269) | 3% (6/238) | -4.2 |
| Comprehensive | East North Central | 2011 | 11% (52/460) | 15% (37/250) | 7% (15/210) | -7.7 |
| Comprehensive | East North Central | 2012 | 21% (104/507) | 28% (80/284) | 11% (24/223) | -17.4 |
| Comprehensive | East North Central | 2013 | 35% (168/485) | 46% (125/270) | 20% (43/215) | -26.3 |
| Comprehensive | East North Central | 2014 | 42% (195/460) | 53% (136/259) | 29% (59/201) | -23.2 |
| Comprehensive | East North Central | 2015 | 53% (251/478) | 62% (167/270) | 40% (84/208) | -21.5 |
| Comprehensive | East North Central | 2016 | 57% (279/486) | 69% (186/269) | 43% (93/217) | -26.3 |
| Comprehensive | East North Central | 2017 | 65% (303/468) | 78% (207/267) | 48% (96/201) | -29.8 |
| Comprehensive | East North Central | 2018 | 73% (360/490) | 86% (248/289) | 56% (112/201) | -30.1 |
| Comprehensive | East North Central | 2020 | 81% (369/455) | 89% (227/256) | 71% (142/199) | -17.3 |
| Comprehensive | East South Central | 2008 | 2% (4/204) | 0% (0/88) | 3% (4/116) | 3.4 |
| Comprehensive | East South Central | 2009 | 2% (5/268) | 1% (1/109) | 3% (4/159) | 1.6 |
| Comprehensive | East South Central | 2010 | 2% (5/210) | 3% (3/91) | 2% (2/119) | -1.6 |
| Comprehensive | East South Central | 2011 | 4% (8/190) | 4% (3/80) | 5% (5/110) | 0.8 |
| Comprehensive | East South Central | 2012 | 13% (25/199) | 10% (8/81) | 14% (17/118) | 4.5 |
| Comprehensive | East South Central | 2013 | 18% (33/179) | 16% (11/69) | 20% (22/110) | 4.1 |
| Comprehensive | East South Central | 2014 | 29% (50/175) | 35% (28/79) | 23% (22/96) | -12.5 |
| Comprehensive | East South Central | 2015 | 47% (77/164) | 55% (42/77) | 40% (35/87) | -14.3 |
| Comprehensive | East South Central | 2016 | 52% (100/191) | 64% (59/92) | 41% (41/99) | -22.7 |
| Comprehensive | East South Central | 2017 | 49% (93/188) | 54% (49/91) | 45% (44/97) | -8.5 |
| Comprehensive | East South Central | 2018 | 68% (119/176) | 74% (67/90) | 60% (52/86) | -14 |
| Comprehensive | East South Central | 2020 | 69% (127/184) | 79% (63/80) | 62% (64/104) | -17.2 |
| Comprehensive | Middle Atlantic | 2008 | 2% (5/271) | 2% (4/207) | 2% (1/64) | -0.4 |
| Comprehensive | Middle Atlantic | 2009 | 1% (2/298) | 1% (2/218) | 0% (0/80) | -0.9 |
| Comprehensive | Middle Atlantic | 2010 | 2% (6/294) | 3% (6/219) | 0% (0/75) | -2.7 |
| Comprehensive | Middle Atlantic | 2011 | 5% (13/268) | 5% (10/200) | 4% (3/68) | -0.6 |
| Comprehensive | Middle Atlantic | 2012 | 6% (17/278) | 8% (17/211) | 0% (0/67) | -8.1 |
| Comprehensive | Middle Atlantic | 2013 | 21% (59/277) | 23% (49/214) | 16% (10/63) | -7 |
| Comprehensive | Middle Atlantic | 2014 | 29% (77/264) | 32% (64/203) | 21% (13/61) | -10.2 |
| Comprehensive | Middle Atlantic | 2015 | 39% (104/264) | 42% (87/206) | 29% (17/58) | -12.9 |
| Comprehensive | Middle Atlantic | 2016 | 50% (142/282) | 54% (117/217) | 38% (25/65) | -15.5 |
| Comprehensive | Middle Atlantic | 2017 | 50% (139/278) | 56% (123/219) | 27% (16/59) | -29 |
| Comprehensive | Middle Atlantic | 2018 | 64% (176/276) | 68% (142/210) | 52% (34/66) | -16.1 |
| Comprehensive | Middle Atlantic | 2020 | 75% (180/240) | 80% (152/190) | 56% (28/50) | -24 |
| Comprehensive | Mountain | 2008 | 2% (3/198) | 4% (3/77) | 0% (0/121) | -3.9 |
| Comprehensive | Mountain | 2009 | 2% (5/229) | 2% (2/92) | 2% (3/137) | 0 |
| Comprehensive | Mountain | 2010 | 2% (5/204) | 5% (4/77) | 1% (1/127) | -4.4 |
| Comprehensive | Mountain | 2011 | 5% (10/187) | 8% (6/76) | 4% (4/111) | -4.3 |
| Comprehensive | Mountain | 2012 | 15% (29/196) | 25% (20/80) | 8% (9/116) | -17.2 |
| Comprehensive | Mountain | 2013 | 31% (64/209) | 48% (44/91) | 17% (20/118) | -31.4 |
| Comprehensive | Mountain | 2014 | 34% (70/203) | 51% (47/92) | 21% (23/111) | -30.4 |
| Comprehensive | Mountain | 2015 | 34% (70/207) | 41% (35/85) | 29% (35/122) | -12.5 |
| Comprehensive | Mountain | 2016 | 53% (109/204) | 75% (73/97) | 34% (36/107) | -41.6 |
| Comprehensive | Mountain | 2017 | 56% (122/217) | 79% (80/101) | 36% (42/116) | -43 |
| Comprehensive | Mountain | 2018 | 63% (133/212) | 79% (78/99) | 49% (55/113) | -30.1 |
| Comprehensive | Mountain | 2020 | 68% (143/210) | 81% (85/105) | 55% (58/105) | -25.7 |
| Comprehensive | New England | 2008 | 0% (0/130) | 0% (0/73) | 0% (0/57) | 0 |
| Comprehensive | New England | 2009 | 2% (3/139) | 3% (3/90) | 0% (0/49) | -3.3 |
| Comprehensive | New England | 2010 | 4% (6/144) | 6% (5/89) | 2% (1/55) | -3.8 |
| Comprehensive | New England | 2011 | 12% (14/115) | 15% (11/75) | 8% (3/40) | -7.2 |
| Comprehensive | New England | 2012 | 11% (13/116) | 14% (10/73) | 7% (3/43) | -6.7 |
| Comprehensive | New England | 2013 | 23% (24/105) | 25% (17/68) | 19% (7/37) | -6.1 |
| Comprehensive | New England | 2014 | 30% (32/105) | 35% (25/71) | 21% (7/34) | -14.6 |
| Comprehensive | New England | 2015 | 30% (31/105) | 35% (23/66) | 21% (8/39) | -14.3 |
| Comprehensive | New England | 2016 | 47% (56/119) | 58% (42/72) | 30% (14/47) | -28.5 |
| Comprehensive | New England | 2017 | 47% (54/114) | 65% (44/68) | 22% (10/46) | -43 |
| Comprehensive | New England | 2018 | 65% (81/125) | 76% (57/75) | 48% (24/50) | -28 |
| Comprehensive | New England | 2020 | 72% (71/98) | 84% (51/61) | 54% (20/37) | -29.6 |
| Comprehensive | Pacific | 2008 | 3% (7/279) | 3% (5/194) | 2% (2/85) | -0.2 |
| Comprehensive | Pacific | 2009 | 3% (10/317) | 5% (10/221) | 0% (0/96) | -4.5 |
| Comprehensive | Pacific | 2010 | 4% (11/289) | 5% (11/201) | 0% (0/88) | -5.5 |
| Comprehensive | Pacific | 2011 | 13% (32/255) | 16% (27/172) | 6% (5/83) | -9.7 |
| Comprehensive | Pacific | 2012 | 21% (61/297) | 27% (56/206) | 5% (5/91) | -21.7 |
| Comprehensive | Pacific | 2013 | 26% (67/256) | 29% (53/184) | 19% (14/72) | -9.4 |
| Comprehensive | Pacific | 2014 | 36% (95/264) | 41% (77/187) | 23% (18/77) | -17.8 |
| Comprehensive | Pacific | 2015 | 43% (134/314) | 48% (106/222) | 30% (28/92) | -17.3 |
| Comprehensive | Pacific | 2016 | 55% (152/277) | 64% (121/190) | 36% (31/87) | -28.1 |
| Comprehensive | Pacific | 2017 | 56% (161/285) | 61% (126/205) | 44% (35/80) | -17.7 |
| Comprehensive | Pacific | 2018 | 70% (183/261) | 72% (134/185) | 64% (49/76) | -8 |
| Comprehensive | Pacific | 2020 | 74% (165/224) | 78% (124/159) | 63% (41/65) | -14.9 |
| Comprehensive | South Atlantic | 2008 | 1% (6/434) | 2% (6/309) | 0% (0/125) | -1.9 |
| Comprehensive | South Atlantic | 2009 | 4% (16/424) | 4% (12/282) | 3% (4/142) | -1.4 |
| Comprehensive | South Atlantic | 2010 | 4% (15/418) | 4% (11/276) | 3% (4/142) | -1.2 |
| Comprehensive | South Atlantic | 2011 | 11% (42/368) | 14% (37/263) | 5% (5/105) | -9.3 |
| Comprehensive | South Atlantic | 2012 | 21% (79/377) | 25% (65/260) | 12% (14/117) | -13 |
| Comprehensive | South Atlantic | 2013 | 33% (111/341) | 39% (97/246) | 15% (14/95) | -24.7 |
| Comprehensive | South Atlantic | 2014 | 38% (151/397) | 40% (119/296) | 32% (32/101) | -8.5 |
| Comprehensive | South Atlantic | 2015 | 41% (173/425) | 44% (137/312) | 32% (36/113) | -12.1 |
| Comprehensive | South Atlantic | 2016 | 61% (286/471) | 67% (228/342) | 45% (58/129) | -21.7 |
| Comprehensive | South Atlantic | 2017 | 64% (298/465) | 69% (240/349) | 50% (58/116) | -18.8 |
| Comprehensive | South Atlantic | 2018 | 74% (340/461) | 80% (268/336) | 58% (72/125) | -22.2 |
| Comprehensive | South Atlantic | 2020 | 83% (374/449) | 86% (284/330) | 76% (90/119) | -10.4 |
| Comprehensive | West North Central | 2008 | 2% (8/429) | 4% (4/100) | 1% (4/329) | -2.8 |
| Comprehensive | West North Central | 2009 | 4% (24/539) | 10% (14/142) | 3% (10/397) | -7.3 |
| Comprehensive | West North Central | 2010 | 5% (26/539) | 11% (15/140) | 3% (11/399) | -8 |
| Comprehensive | West North Central | 2011 | 8% (44/528) | 15% (21/142) | 6% (23/386) | -8.8 |
| Comprehensive | West North Central | 2012 | 18% (95/519) | 27% (38/140) | 15% (57/379) | -12.1 |
| Comprehensive | West North Central | 2013 | 23% (116/502) | 36% (50/137) | 18% (66/365) | -18.4 |
| Comprehensive | West North Central | 2014 | 37% (187/502) | 49% (66/135) | 33% (121/367) | -15.9 |
| Comprehensive | West North Central | 2015 | 43% (227/532) | 58% (80/137) | 37% (147/395) | -21.2 |
| Comprehensive | West North Central | 2016 | 53% (275/518) | 77% (105/137) | 45% (170/381) | -32 |
| Comprehensive | West North Central | 2017 | 58% (286/495) | 78% (106/136) | 50% (180/359) | -27.8 |
| Comprehensive | West North Central | 2018 | 68% (337/492) | 87% (117/134) | 61% (220/358) | -25.9 |
| Comprehensive | West North Central | 2020 | 68% (287/421) | 88% (106/121) | 60% (181/300) | -27.3 |
| Comprehensive | West South Central | 2008 | 1% (5/407) | 1% (2/220) | 2% (3/187) | 0.7 |
| Comprehensive | West South Central | 2009 | 2% (7/462) | 2% (5/239) | 1% (2/223) | -1.2 |
| Comprehensive | West South Central | 2010 | 3% (14/412) | 4% (8/220) | 3% (6/192) | -0.5 |
| Comprehensive | West South Central | 2011 | 7% (22/322) | 8% (13/164) | 6% (9/158) | -2.2 |
| Comprehensive | West South Central | 2012 | 18% (80/434) | 22% (52/237) | 14% (28/197) | -7.7 |
| Comprehensive | West South Central | 2013 | 25% (93/369) | 26% (53/200) | 24% (40/169) | -2.8 |
| Comprehensive | West South Central | 2014 | 32% (125/385) | 36% (78/216) | 28% (47/169) | -8.3 |
| Comprehensive | West South Central | 2015 | 30% (107/360) | 36% (72/199) | 22% (35/161) | -14.4 |
| Comprehensive | West South Central | 2016 | 48% (182/376) | 62% (127/205) | 32% (55/171) | -29.8 |
| Comprehensive | West South Central | 2017 | 52% (183/354) | 65% (126/195) | 36% (57/159) | -28.8 |
| Comprehensive | West South Central | 2018 | 58% (222/381) | 69% (140/202) | 46% (82/179) | -23.5 |
| Comprehensive | West South Central | 2020 | 71% (267/377) | 81% (166/206) | 59% (101/171) | -21.5 |

For each core EHR capability, Census division, and survey year, the table presents adoption percentages and counts (n/N) for all hospitals, urban hospitals, and rural hospitals, along with the rural minus urban percentage-point gap.

**Supplemental Table 10. Logistic regression models for core EHR adoption by Census division, 2008-2023**

| **Measure** | **Census division** | **Model** | **Term** | **Odds ratio (95% CI)** |
| --- | --- | --- | --- | --- |
| Basic - with notes | East North Central | Adjusted | Year | 1.82 (1.72-1.93) |
| Basic - with notes | East North Central | Adjusted | Rural (reference: urban) | 0.90 (0.84-0.97) |
| Basic - with notes | East North Central | Unadjusted | Year | 1.80 (1.70-1.91) |
| Basic - with notes | East North Central | Unadjusted | Rural (reference: urban) | 0.91 (0.84-0.97) |
| Basic - with notes | East South Central | Adjusted | Year | 1.73 (1.60-1.88) |
| Basic - with notes | East South Central | Adjusted | Rural (reference: urban) | 0.89 (0.81-0.99) |
| Basic - with notes | East South Central | Unadjusted | Year | 1.73 (1.60-1.88) |
| Basic - with notes | East South Central | Unadjusted | Rural (reference: urban) | 0.89 (0.81-0.99) |
| Basic - with notes | Middle Atlantic | Adjusted | Year | 1.58 (1.51-1.65) |
| Basic - with notes | Middle Atlantic | Adjusted | Rural (reference: urban) | 1.01 (0.93-1.09) |
| Basic - with notes | Middle Atlantic | Unadjusted | Year | 1.55 (1.49-1.62) |
| Basic - with notes | Middle Atlantic | Unadjusted | Rural (reference: urban) | 1.00 (0.93-1.08) |
| Basic - with notes | Mountain | Adjusted | Year | 1.84 (1.68-2.01) |
| Basic - with notes | Mountain | Adjusted | Rural (reference: urban) | 0.85 (0.77-0.95) |
| Basic - with notes | Mountain | Unadjusted | Year | 1.83 (1.67-2.00) |
| Basic - with notes | Mountain | Unadjusted | Rural (reference: urban) | 0.85 (0.77-0.95) |
| Basic - with notes | New England | Adjusted | Year | 1.62 (1.50-1.76) |
| Basic - with notes | New England | Adjusted | Rural (reference: urban) | 0.98 (0.87-1.10) |
| Basic - with notes | New England | Unadjusted | Year | 1.60 (1.48-1.72) |
| Basic - with notes | New England | Unadjusted | Rural (reference: urban) | 0.97 (0.87-1.09) |
| Basic - with notes | Pacific | Adjusted | Year | 1.63 (1.54-1.73) |
| Basic - with notes | Pacific | Adjusted | Rural (reference: urban) | 0.98 (0.89-1.08) |
| Basic - with notes | Pacific | Unadjusted | Year | 1.62 (1.53-1.71) |
| Basic - with notes | Pacific | Unadjusted | Rural (reference: urban) | 0.98 (0.89-1.07) |
| Basic - with notes | South Atlantic | Adjusted | Year | 1.68 (1.61-1.75) |
| Basic - with notes | South Atlantic | Adjusted | Rural (reference: urban) | 0.98 (0.91-1.05) |
| Basic - with notes | South Atlantic | Unadjusted | Year | 1.67 (1.60-1.74) |
| Basic - with notes | South Atlantic | Unadjusted | Rural (reference: urban) | 0.97 (0.91-1.04) |
| Basic - with notes | West North Central | Adjusted | Year | 1.68 (1.55-1.81) |
| Basic - with notes | West North Central | Adjusted | Rural (reference: urban) | 0.94 (0.87-1.02) |
| Basic - with notes | West North Central | Unadjusted | Year | 1.65 (1.54-1.78) |
| Basic - with notes | West North Central | Unadjusted | Rural (reference: urban) | 0.95 (0.87-1.02) |
| Basic - with notes | West South Central | Adjusted | Year | 1.73 (1.62-1.80) |
| Basic - with notes | West South Central | Adjusted | Rural (reference: urban) | 0.89 (0.83-0.96) |
| Basic - with notes | West South Central | Unadjusted | Year | 1.68 (1.59-1.78) |
| Basic - with notes | West South Central | Unadjusted | Rural (reference: urban) | 0.90 (0.83-0.96) |
| Basic - without notes | East North Central | Adjusted | Year | 1.87 (1.75-1.99) |
| Basic - without notes | East North Central | Adjusted | Rural (reference: urban) | 0.89 (0.82-0.97) |
| Basic - without notes | East North Central | Unadjusted | Year | 1.84 (1.73-1.96) |
| Basic - without notes | East North Central | Unadjusted | Rural (reference: urban) | 0.89 (0.82-0.97) |
| Basic - without notes | East South Central | Adjusted | Year | 1.79 (1.62-1.98) |
| Basic - without notes | East South Central | Adjusted | Rural (reference: urban) | 0.87 (0.77-0.98) |
| Basic - without notes | East South Central | Unadjusted | Year | 1.78 (1.61-1.96) |
| Basic - without notes | East South Central | Unadjusted | Rural (reference: urban) | 0.87 (0.77-0.98) |
| Basic - without notes | Middle Atlantic | Adjusted | Year | 1.67 (1.57-1.77) |
| Basic - without notes | Middle Atlantic | Adjusted | Rural (reference: urban) | 0.99 (0.89-1.09) |
| Basic - without notes | Middle Atlantic | Unadjusted | Year | 1.63 (1.54-1.73) |
| Basic - without notes | Middle Atlantic | Unadjusted | Rural (reference: urban) | 0.98 (0.88-1.08) |
| Basic - without notes | Mountain | Adjusted | Year | 1.82 (1.65-2.00) |
| Basic - without notes | Mountain | Adjusted | Rural (reference: urban) | 0.85 (0.76-0.95) |
| Basic - without notes | Mountain | Unadjusted | Year | 1.82 (1.65-2.00) |
| Basic - without notes | Mountain | Unadjusted | Rural (reference: urban) | 0.85 (0.76-0.95) |
| Basic - without notes | New England | Adjusted | Year | 1.59 (1.47-1.72) |
| Basic - without notes | New England | Adjusted | Rural (reference: urban) | 1.00 (0.87-1.13) |
| Basic - without notes | New England | Unadjusted | Year | 1.56 (1.45-1.69) |
| Basic - without notes | New England | Unadjusted | Rural (reference: urban) | 0.99 (0.88-1.13) |
| Basic - without notes | Pacific | Adjusted | Year | 1.65 (1.56-1.75) |
| Basic - without notes | Pacific | Adjusted | Rural (reference: urban) | 0.98 (0.89-1.08) |
| Basic - without notes | Pacific | Unadjusted | Year | 1.64 (1.55-1.74) |
| Basic - without notes | Pacific | Unadjusted | Rural (reference: urban) | 0.97 (0.88-1.07) |
| Basic - without notes | South Atlantic | Adjusted | Year | 1.70 (1.62-1.78) |
| Basic - without notes | South Atlantic | Adjusted | Rural (reference: urban) | 0.95 (0.88-1.02) |
| Basic - without notes | South Atlantic | Unadjusted | Year | 1.67 (1.60-1.76) |
| Basic - without notes | South Atlantic | Unadjusted | Rural (reference: urban) | 0.95 (0.88-1.02) |
| Basic - without notes | West North Central | Adjusted | Year | 1.80 (1.66-1.96) |
| Basic - without notes | West North Central | Adjusted | Rural (reference: urban) | 0.87 (0.80-0.96) |
| Basic - without notes | West North Central | Unadjusted | Year | 1.77 (1.63-1.93) |
| Basic - without notes | West North Central | Unadjusted | Rural (reference: urban) | 0.87 (0.80-0.96) |
| Basic - without notes | West South Central | Adjusted | Year | 1.70 (1.61-1.80) |
| Basic - without notes | West South Central | Adjusted | Rural (reference: urban) | 0.89 (0.84-0.96) |
| Basic - without notes | West South Central | Unadjusted | Year | 1.67 (1.57-1.76) |
| Basic - without notes | West South Central | Unadjusted | Rural (reference: urban) | 0.90 (0.84-0.95) |
| Certified EHR (CEHRT) | East North Central | Adjusted | Year | 1.99 (1.75-2.28) |
| Certified EHR (CEHRT) | East North Central | Adjusted | Rural (reference: urban) | 0.85 (0.72-1.01) |
| Certified EHR (CEHRT) | East North Central | Unadjusted | Year | 1.95 (1.72-2.21) |
| Certified EHR (CEHRT) | East North Central | Unadjusted | Rural (reference: urban) | 0.86 (0.73-1.02) |
| Certified EHR (CEHRT) | East South Central | Adjusted | Year | 2.23 (1.68-2.97) |
| Certified EHR (CEHRT) | East South Central | Adjusted | Rural (reference: urban) | 0.69 (0.50-0.94) |
| Certified EHR (CEHRT) | East South Central | Unadjusted | Year | 2.21 (1.67-2.93) |
| Certified EHR (CEHRT) | East South Central | Unadjusted | Rural (reference: urban) | 0.69 (0.50-0.95) |
| Certified EHR (CEHRT) | Middle Atlantic | Adjusted | Year | 1.61 (1.40-1.85) |
| Certified EHR (CEHRT) | Middle Atlantic | Adjusted | Rural (reference: urban) | 0.87 (0.63-1.22) |
| Certified EHR (CEHRT) | Middle Atlantic | Unadjusted | Year | 1.60 (1.39-1.83) |
| Certified EHR (CEHRT) | Middle Atlantic | Unadjusted | Rural (reference: urban) | 0.87 (0.63-1.21) |
| Certified EHR (CEHRT) | Mountain | Adjusted | Year | 1.60 (1.39-1.83) |
| Certified EHR (CEHRT) | Mountain | Adjusted | Rural (reference: urban) | 0.91 (0.75-1.10) |
| Certified EHR (CEHRT) | Mountain | Unadjusted | Year | 1.60 (1.40-1.83) |
| Certified EHR (CEHRT) | Mountain | Unadjusted | Rural (reference: urban) | 0.90 (0.75-1.09) |
| Certified EHR (CEHRT) | New England | Adjusted | Year | 1.76 (1.50-2.06) |
| Certified EHR (CEHRT) | New England | Adjusted | Rural (reference: urban) | 0.80 (0.61-1.04) |
| Certified EHR (CEHRT) | New England | Unadjusted | Year | 1.74 (1.48-2.06) |
| Certified EHR (CEHRT) | New England | Unadjusted | Rural (reference: urban) | 0.81 (0.62-1.05) |
| Certified EHR (CEHRT) | Pacific | Adjusted | Year | 1.69 (1.52-1.87) |
| Certified EHR (CEHRT) | Pacific | Adjusted | Rural (reference: urban) | 1.04 (0.88-1.23) |
| Certified EHR (CEHRT) | Pacific | Unadjusted | Year | 1.69 (1.53-1.86) |
| Certified EHR (CEHRT) | Pacific | Unadjusted | Rural (reference: urban) | 1.03 (0.87-1.22) |
| Certified EHR (CEHRT) | South Atlantic | Adjusted | Year | 1.85 (1.67-2.05) |
| Certified EHR (CEHRT) | South Atlantic | Adjusted | Rural (reference: urban) | 1.00 (0.81-1.24) |
| Certified EHR (CEHRT) | South Atlantic | Unadjusted | Year | 1.83 (1.66-2.03) |
| Certified EHR (CEHRT) | South Atlantic | Unadjusted | Rural (reference: urban) | 0.99 (0.80-1.22) |
| Certified EHR (CEHRT) | West North Central | Adjusted | Year | 1.70 (1.37-2.10) |
| Certified EHR (CEHRT) | West North Central | Adjusted | Rural (reference: urban) | 0.93 (0.74-1.17) |
| Certified EHR (CEHRT) | West North Central | Unadjusted | Year | 1.67 (1.37-2.04) |
| Certified EHR (CEHRT) | West North Central | Unadjusted | Rural (reference: urban) | 0.93 (0.75-1.15) |
| Certified EHR (CEHRT) | West South Central | Adjusted | Year | 1.73 (1.49-2.00) |
| Certified EHR (CEHRT) | West South Central | Adjusted | Rural (reference: urban) | 0.78 (0.64-0.95) |
| Certified EHR (CEHRT) | West South Central | Unadjusted | Year | 1.67 (1.46-1.90) |
| Certified EHR (CEHRT) | West South Central | Unadjusted | Rural (reference: urban) | 0.79 (0.66-0.95) |
| Comprehensive | East North Central | Adjusted | Year | 1.64 (1.57-1.72) |
| Comprehensive | East North Central | Adjusted | Rural (reference: urban) | 0.92 (0.87-0.98) |
| Comprehensive | East North Central | Unadjusted | Year | 1.62 (1.55-1.69) |
| Comprehensive | East North Central | Unadjusted | Rural (reference: urban) | 0.93 (0.88-0.99) |
| Comprehensive | East South Central | Adjusted | Year | 1.68 (1.57-1.81) |
| Comprehensive | East South Central | Adjusted | Rural (reference: urban) | 0.88 (0.80-0.96) |
| Comprehensive | East South Central | Unadjusted | Year | 1.66 (1.55-1.79) |
| Comprehensive | East South Central | Unadjusted | Rural (reference: urban) | 0.87 (0.80-0.95) |
| Comprehensive | Middle Atlantic | Adjusted | Year | 1.61 (1.54-1.68) |
| Comprehensive | Middle Atlantic | Adjusted | Rural (reference: urban) | 0.94 (0.87-1.02) |
| Comprehensive | Middle Atlantic | Unadjusted | Year | 1.60 (1.53-1.67) |
| Comprehensive | Middle Atlantic | Unadjusted | Rural (reference: urban) | 0.94 (0.87-1.01) |
| Comprehensive | Mountain | Adjusted | Year | 1.58 (1.47-1.69) |
| Comprehensive | Mountain | Adjusted | Rural (reference: urban) | 0.93 (0.85-1.02) |
| Comprehensive | Mountain | Unadjusted | Year | 1.56 (1.46-1.67) |
| Comprehensive | Mountain | Unadjusted | Rural (reference: urban) | 0.93 (0.85-1.01) |
| Comprehensive | New England | Adjusted | Year | 1.61 (1.48-1.75) |
| Comprehensive | New England | Adjusted | Rural (reference: urban) | 0.90 (0.80-1.02) |
| Comprehensive | New England | Unadjusted | Year | 1.59 (1.47-1.72) |
| Comprehensive | New England | Unadjusted | Rural (reference: urban) | 0.90 (0.80-1.02) |
| Comprehensive | Pacific | Adjusted | Year | 1.50 (1.44-1.56) |
| Comprehensive | Pacific | Adjusted | Rural (reference: urban) | 1.06 (0.98-1.14) |
| Comprehensive | Pacific | Unadjusted | Year | 1.48 (1.43-1.54) |
| Comprehensive | Pacific | Unadjusted | Rural (reference: urban) | 1.04 (0.96-1.12) |
| Comprehensive | South Atlantic | Adjusted | Year | 1.58 (1.52-1.63) |
| Comprehensive | South Atlantic | Adjusted | Rural (reference: urban) | 0.98 (0.92-1.04) |
| Comprehensive | South Atlantic | Unadjusted | Year | 1.57 (1.51-1.62) |
| Comprehensive | South Atlantic | Unadjusted | Rural (reference: urban) | 0.98 (0.92-1.05) |
| Comprehensive | West North Central | Adjusted | Year | 1.62 (1.52-1.73) |
| Comprehensive | West North Central | Adjusted | Rural (reference: urban) | 0.91 (0.84-0.97) |
| Comprehensive | West North Central | Unadjusted | Year | 1.59 (1.50-1.70) |
| Comprehensive | West North Central | Unadjusted | Rural (reference: urban) | 0.91 (0.85-0.98) |
| Comprehensive | West South Central | Adjusted | Year | 1.57 (1.50-1.63) |
| Comprehensive | West South Central | Adjusted | Rural (reference: urban) | 0.89 (0.84-0.94) |
| Comprehensive | West South Central | Unadjusted | Year | 1.56 (1.50-1.62) |
| Comprehensive | West South Central | Unadjusted | Rural (reference: urban) | 0.89 (0.84-0.94) |

Logistic regression estimates stratified by U.S. Census division, summarizing time trends and rural–urban differences in core EHR adoption.

**Supplemental Table 11. Core EHR adoption by four‑category RUCA rurality, 2008-2023**

| **Measure** | **Year** | **Rurality** | **Adoption % (n/N)** | **Gap** |
| --- | --- | --- | --- | --- |
| Basic - with notes | 2008 | Micropolitan | 8% (41/504) | -3.7 |
| Basic - with notes | 2009 | Micropolitan | 12% (70/564) | -3.4 |
| Basic - with notes | 2010 | Micropolitan | 14% (74/537) | -6.6 |
| Basic - with notes | 2011 | Micropolitan | 24% (111/468) | -10 |
| Basic - with notes | 2012 | Micropolitan | 43% (212/494) | -8.9 |
| Basic - with notes | 2013 | Micropolitan | 58% (265/456) | -7.7 |
| Basic - with notes | 2014 | Micropolitan | 75% (341/452) | -5.2 |
| Basic - with notes | 2015 | Micropolitan | 86% (409/475) | -0.9 |
| Basic - with notes | 2016 | Micropolitan | 90% (445/497) | -0.9 |
| Basic - with notes | 2017 | Micropolitan | 72% (348/480) | -12.4 |
| Basic - with notes | 2018 | Micropolitan | 89% (433/488) | -5.2 |
| Basic - with notes | 2020 | Micropolitan | 93% (441/473) | -3 |
| Basic - with notes | 2008 | Small town | 6% (33/538) | -5.7 |
| Basic - with notes | 2009 | Small town | 7% (43/653) | -9.3 |
| Basic - with notes | 2010 | Small town | 12% (68/587) | -8.8 |
| Basic - with notes | 2011 | Small town | 25% (132/523) | -8.7 |
| Basic - with notes | 2012 | Small town | 36% (205/573) | -16.1 |
| Basic - with notes | 2013 | Small town | 50% (228/453) | -15.5 |
| Basic - with notes | 2014 | Small town | 67% (297/441) | -11.3 |
| Basic - with notes | 2015 | Small town | 80% (350/438) | -6.7 |
| Basic - with notes | 2016 | Small town | 84% (369/441) | -7.8 |
| Basic - with notes | 2017 | Small town | 70% (299/429) | -14.8 |
| Basic - with notes | 2018 | Small town | 85% (360/423) | -8.5 |
| Basic - with notes | 2020 | Small town | 92% (349/380) | -4.4 |
| Basic - with notes | 2008 | Rural | 5% (17/321) | -6.9 |
| Basic - with notes | 2009 | Rural | 9% (32/347) | -7.3 |
| Basic - with notes | 2010 | Rural | 14% (43/298) | -7.1 |
| Basic - with notes | 2011 | Rural | 25% (70/282) | -8.6 |
| Basic - with notes | 2012 | Rural | 42% (111/265) | -11.2 |
| Basic - with notes | 2013 | Rural | 55% (124/225) | -12.8 |
| Basic - with notes | 2014 | Rural | 77% (163/211) | -6.8 |
| Basic - with notes | 2015 | Rural | 88% (186/212) | -3.2 |
| Basic - with notes | 2016 | Rural | 91% (199/219) | -3.6 |
| Basic - with notes | 2017 | Rural | 80% (173/217) | -6.8 |
| Basic - with notes | 2018 | Rural | 93% (207/222) | -2.8 |
| Basic - with notes | 2020 | Rural | 96% (182/190) | -1.4 |
| Basic - without notes | 2008 | Micropolitan | 8% (41/504) | -4.6 |
| Basic - without notes | 2009 | Micropolitan | 14% (78/564) | -3.4 |
| Basic - without notes | 2010 | Micropolitan | 19% (103/537) | -5 |
| Basic - without notes | 2011 | Micropolitan | 36% (167/468) | -7.8 |
| Basic - without notes | 2012 | Micropolitan | 54% (266/494) | -6.7 |
| Basic - without notes | 2013 | Micropolitan | 65% (297/456) | -10 |
| Basic - without notes | 2014 | Micropolitan | 84% (379/452) | -4.2 |
| Basic - without notes | 2015 | Micropolitan | 92% (436/475) | -1.4 |
| Basic - without notes | 2016 | Micropolitan | 95% (472/497) | -1.3 |
| Basic - without notes | 2017 | Micropolitan | 84% (404/480) | -7.7 |
| Basic - without notes | 2018 | Micropolitan | 96% (468/488) | -2.4 |
| Basic - without notes | 2020 | Micropolitan | 98% (463/473) | -0.8 |
| Basic - without notes | 2021 | Micropolitan | 99% (459/464) | -0.2 |
| Basic - without notes | 2022 | Micropolitan | 99% (460/466) | -0.4 |
| Basic - without notes | 2023 | Micropolitan | 99% (460/463) | -0.1 |
| Basic - without notes | 2008 | Small town | 7% (36/538) | -6.5 |
| Basic - without notes | 2009 | Small town | 9% (58/653) | -7.8 |
| Basic - without notes | 2010 | Small town | 15% (90/587) | -7.2 |
| Basic - without notes | 2011 | Small town | 39% (205/523) | -5.9 |
| Basic - without notes | 2012 | Small town | 55% (314/573) | -13.5 |
| Basic - without notes | 2013 | Small town | 68% (307/453) | -12 |
| Basic - without notes | 2014 | Small town | 84% (372/441) | -9.3 |
| Basic - without notes | 2015 | Small town | 92% (400/438) | -4.8 |
| Basic - without notes | 2016 | Small town | 95% (418/441) | -4.7 |
| Basic - without notes | 2017 | Small town | 85% (364/429) | -9.6 |
| Basic - without notes | 2018 | Small town | 96% (405/423) | -4.6 |
| Basic - without notes | 2020 | Small town | 97% (368/380) | -2.3 |
| Basic - without notes | 2021 | Small town | 97% (377/387) | -2 |
| Basic - without notes | 2022 | Small town | 97% (379/390) | -2 |
| Basic - without notes | 2023 | Small town | 97% (369/382) | -2.4 |
| Basic - without notes | 2008 | Rural | 7% (22/321) | -6.7 |
| Basic - without notes | 2009 | Rural | 10% (36/347) | -6.5 |
| Basic - without notes | 2010 | Rural | 14% (42/298) | -8 |
| Basic - without notes | 2011 | Rural | 36% (101/282) | -7.3 |
| Basic - without notes | 2012 | Rural | 51% (136/265) | -13 |
| Basic - without notes | 2013 | Rural | 65% (146/225) | -14.8 |
| Basic - without notes | 2014 | Rural | 82% (172/211) | -8.8 |
| Basic - without notes | 2015 | Rural | 93% (196/212) | -3.4 |
| Basic - without notes | 2016 | Rural | 95% (208/219) | -3.3 |
| Basic - without notes | 2017 | Rural | 86% (186/217) | -6.2 |
| Basic - without notes | 2018 | Rural | 97% (216/222) | -2.5 |
| Basic - without notes | 2020 | Rural | 98% (186/190) | -0.9 |
| Basic - without notes | 2021 | Rural | 99% (197/200) | -0.2 |
| Basic - without notes | 2022 | Rural | 99% (236/239) | 0.1 |
| Basic - without notes | 2023 | Rural | 99% (221/223) | -0.1 |
| Comprehensive | 2015 | Micropolitan | 8% (38/475) | -8.8 |
| Comprehensive | 2016 | Micropolitan | 9% (43/497) | -7.2 |
| Comprehensive | 2017 | Micropolitan | 12% (59/480) | -7.7 |
| Comprehensive | 2018 | Micropolitan | 17% (82/488) | -9 |
| Comprehensive | 2020 | Micropolitan | 37% (173/473) | -6.2 |
| Comprehensive | 2021 | Micropolitan | 56% (262/464) | -8.8 |
| Comprehensive | 2022 | Micropolitan | 67% (310/466) | -9.3 |
| Comprehensive | 2023 | Micropolitan | 71% (331/463) | -9.7 |
| Comprehensive | 2015 | Small town | 7% (31/438) | -10.2 |
| Comprehensive | 2016 | Small town | 8% (36/441) | -8.1 |
| Comprehensive | 2017 | Small town | 8% (34/429) | -12.1 |
| Comprehensive | 2018 | Small town | 16% (66/423) | -8.7 |
| Comprehensive | 2020 | Small town | 29% (112/380) | -13.6 |
| Comprehensive | 2021 | Small town | 51% (196/387) | -13 |
| Comprehensive | 2022 | Small town | 60% (235/390) | -13.4 |
| Comprehensive | 2023 | Small town | 66% (251/382) | -11.8 |
| Comprehensive | 2015 | Rural | 10% (20/212) | -8.7 |
| Comprehensive | 2016 | Rural | 11% (23/219) | -7.5 |
| Comprehensive | 2017 | Rural | 9% (19/217) | -15.7 |
| Comprehensive | 2018 | Rural | 21% (47/222) | -11.5 |
| Comprehensive | 2020 | Rural | 33% (62/190) | -14.3 |
| Comprehensive | 2021 | Rural | 50% (100/200) | -14.2 |
| Comprehensive | 2022 | Rural | 57% (136/239) | -16.2 |
| Comprehensive | 2023 | Rural | 62% (138/223) | -13.8 |
| Certified EHR (CEHRT) | 2009 | Micropolitan | 42% (211/504) | -1.4 |
| Certified EHR (CEHRT) | 2011 | Micropolitan | 71% (334/468) | -6.1 |
| Certified EHR (CEHRT) | 2012 | Micropolitan | 87% (429/494) | -4.2 |
| Certified EHR (CEHRT) | 2013 | Micropolitan | 94% (427/456) | -3.2 |
| Certified EHR (CEHRT) | 2014 | Micropolitan | 96% (434/452) | -2.1 |
| Certified EHR (CEHRT) | 2015 | Micropolitan | 97% (464/475) | -2 |
| Certified EHR (CEHRT) | 2016 | Micropolitan | 98% (488/497) | -1.6 |
| Certified EHR (CEHRT) | 2017 | Micropolitan | 98% (472/480) | -2 |
| Certified EHR (CEHRT) | 2018 | Micropolitan | 98% (478/488) | -1.4 |
| Certified EHR (CEHRT) | 2020 | Micropolitan | 98% (462/473) | -1.6 |
| Certified EHR (CEHRT) | 2021 | Micropolitan | 99% (459/464) | -0.7 |
| Certified EHR (CEHRT) | 2022 | Micropolitan | 99% (463/466) | -0.6 |
| Certified EHR (CEHRT) | 2023 | Micropolitan | 99% (459/463) | -0.8 |
| Certified EHR (CEHRT) | 2009 | Small town | 35% (189/538) | -8.1 |
| Certified EHR (CEHRT) | 2011 | Small town | 68% (357/523) | -9.5 |
| Certified EHR (CEHRT) | 2012 | Small town | 85% (486/573) | -6.2 |
| Certified EHR (CEHRT) | 2013 | Small town | 93% (422/453) | -4.2 |
| Certified EHR (CEHRT) | 2014 | Small town | 95% (421/441) | -3.7 |
| Certified EHR (CEHRT) | 2015 | Small town | 96% (422/438) | -3.5 |
| Certified EHR (CEHRT) | 2016 | Small town | 97% (428/441) | -2.7 |
| Certified EHR (CEHRT) | 2017 | Small town | 97% (415/429) | -3.2 |
| Certified EHR (CEHRT) | 2018 | Small town | 97% (411/423) | -2.8 |
| Certified EHR (CEHRT) | 2020 | Small town | 98% (374/380) | -1.7 |
| Certified EHR (CEHRT) | 2021 | Small town | 99% (237/240) | -1.1 |
| Certified EHR (CEHRT) | 2022 | Small town | 100% (277/278) | -0.3 |
| Certified EHR (CEHRT) | 2023 | Small town | 100% (260/260) | 0.3 |
| Certified EHR (CEHRT) | 2009 | Rural | 54% (99/184) | -13.1 |
| Certified EHR (CEHRT) | 2011 | Rural | 75% (168/223) | -8.7 |
| Certified EHR (CEHRT) | 2012 | Rural | 92% (217/235) | -2.2 |
| Certified EHR (CEHRT) | 2013 | Rural | 97% (230/237) | -1.3 |
| Certified EHR (CEHRT) | 2014 | Rural | 97% (229/237) | -1.6 |
| Certified EHR (CEHRT) | 2015 | Rural | 98% (256/261) | -0.7 |
| Certified EHR (CEHRT) | 2016 | Rural | 97% (253/262) | -2.4 |
| Certified EHR (CEHRT) | 2017 | Rural | 97% (229/235) | -1.9 |
| Certified EHR (CEHRT) | 2018 | Rural | 96% (245/256) | -3 |
| Certified EHR (CEHRT) | 2020 | Rural | 99% (204/207) | -0.8 |
| Certified EHR (CEHRT) | 2021 | Rural | 98% (331/338) | -1.9 |
| Certified EHR (CEHRT) | 2022 | Rural | 99% (396/402) | -1.4 |
| Certified EHR (CEHRT) | 2023 | Rural | 99% (389/392) | -0.4 |

Year-specific adoption of core EHR measures across Rural–Urban Commuting Area (RUCA) four-category rurality groups (metropolitan, micropolitan, small town, rural).

**Supplemental Table 12. Logistic regression models for core EHR adoption using four-category RUCA rurality, 2008-2023**

| **Measure** | **Model** | **Term** | **Odds ratio (95% CI)** |
| --- | --- | --- | --- |
| Basic - with notes | Adjusted | Year | 1.68 (1.64-1.71) |
| Basic - with notes | Adjusted | Micropolitan (reference: urban) | 0.97 (0.94-1.01) |
| Basic - with notes | Adjusted | Rural (reference: urban) | 0.92 (0.88-0.96) |
| Basic - with notes | Adjusted | Small town (reference: urban) | 0.94 (0.91-0.97) |
| Basic - with notes | Unadjusted | Year | 1.66 (1.63-1.69) |
| Basic - with notes | Unadjusted | Micropolitan (reference: urban) | 0.97 (0.94-1.00) |
| Basic - with notes | Unadjusted | Rural (reference: urban) | 0.92 (0.89-0.96) |
| Basic - with notes | Unadjusted | Small town (reference: urban) | 0.94 (0.91-0.97) |
| Basic - without notes | Adjusted | Year | 1.71 (1.68-1.75) |
| Basic - without notes | Adjusted | Micropolitan (reference: urban) | 0.96 (0.93-1.00) |
| Basic - without notes | Adjusted | Rural (reference: urban) | 0.89 (0.85-0.93) |
| Basic - without notes | Adjusted | Small town (reference: urban) | 0.92 (0.88-0.95) |
| Basic - without notes | Unadjusted | Year | 1.69 (1.65-1.73) |
| Basic - without notes | Unadjusted | Micropolitan (reference: urban) | 0.96 (0.93-1.00) |
| Basic - without notes | Unadjusted | Rural (reference: urban) | 0.89 (0.86-0.93) |
| Basic - without notes | Unadjusted | Small town (reference: urban) | 0.92 (0.89-0.95) |
| Certified EHR (CEHRT) | Adjusted | Year | 1.76 (1.67-1.85) |
| Certified EHR (CEHRT) | Adjusted | Micropolitan (reference: urban) | 0.91 (0.83-1.00) |
| Certified EHR (CEHRT) | Adjusted | Rural (reference: urban) | 0.82 (0.74-0.92) |
| Certified EHR (CEHRT) | Adjusted | Small town (reference: urban) | 0.88 (0.80-0.96) |
| Certified EHR (CEHRT) | Unadjusted | Year | 1.73 (1.65-1.82) |
| Certified EHR (CEHRT) | Unadjusted | Micropolitan (reference: urban) | 0.91 (0.83-1.00) |
| Certified EHR (CEHRT) | Unadjusted | Rural (reference: urban) | 0.83 (0.75-0.92) |
| Certified EHR (CEHRT) | Unadjusted | Small town (reference: urban) | 0.88 (0.81-0.97) |
| Comprehensive | Adjusted | Year | 1.58 (1.56-1.61) |
| Comprehensive | Adjusted | Micropolitan (reference: urban) | 0.96 (0.93-0.99) |
| Comprehensive | Adjusted | Rural (reference: urban) | 0.90 (0.87-0.94) |
| Comprehensive | Adjusted | Small town (reference: urban) | 0.93 (0.90-0.95) |
| Comprehensive | Unadjusted | Year | 1.56 (1.54-1.59) |
| Comprehensive | Unadjusted | Micropolitan (reference: urban) | 0.96 (0.93-0.99) |
| Comprehensive | Unadjusted | Rural (reference: urban) | 0.90 (0.87-0.94) |
| Comprehensive | Unadjusted | Small town (reference: urban) | 0.93 (0.90-0.96) |

Logistic regression estimates summarizing time trends and rural gradients in core EHR adoption using the four-category RUCA classification.

**Supplemental Table 13. Health IT survey response rates, 2011-2015**

| **Year** | **Overall response rate** | **Urban response rate** | **Rural response rate** | **Rural - Urban (percentage points)** |
| --- | --- | --- | --- | --- |
| 2011 | 2,680/4,759 (56.31%) | 1,415/2,591 (54.61%) | 1,265/2,161 (58.54%) | 3.93 |
| 2012 | 2,876/4,665 (61.65%) | 1,537/2,519 (61.02%) | 1,339/2,142 (62.51%) | 1.50 |
| 2013 | 2,702/4,647 (58.15%) | 1,462/2,503 (58.41%) | 1,240/2,140 (57.94%) | -0.47 |
| 2014 | 2,724/4,573 (59.57%) | 1,511/2,447 (61.75%) | 1,213/2,122 (57.16%) | -4.59 |
| 2015 | 2,823/4,552 (62.02%) | 1,561/2,444 (63.87%) | 1,262/2,103 (60.01%) | -3.86 |

Overall, urban, and rural response rates. Rural-urban differences are shown in percentage points.

**Supplemental Table 14. Distribution of nonresponse weights, 2011-2015**

| **Year** | **n_resp** | **mean_w** | **sd_w** | **p01** | **p05** | **p50** | **p95** | **p99** | **min_w** | **max_w** |
| --- | --- | --- | --- | --- | --- | --- | --- | --- | --- | --- |
| 2011 | 2,693 | 1.78 | 0.64 | 1.02 | 1.04 | 1.62 | 3.07 | 4.45 | 1.02 | 4.45 |
| 2012 | 2,923 | 1.62 | 0.47 | 1.03 | 1.06 | 1.51 | 2.54 | 3.72 | 1.03 | 3.72 |
| 2013 | 2,723 | 1.72 | 0.65 | 1.01 | 1.03 | 1.55 | 3.05 | 4.6 | 1.01 | 4.61 |
| 2014 | 2,755 | 1.68 | 0.51 | 1.02 | 1.04 | 1.54 | 2.65 | 3.73 | 1.02 | 3.73 |
| 2015 | 2,849 | 1.61 | 0.45 | 1.02 | 1.04 | 1.51 | 2.51 | 3.36 | 1.02 | 3.36 |

Summary statistics for trimmed, scaled inverse-probability response weights among respondents. Abbreviations: n_resp, number of responding hospitals; sd_w, standard deviation; p01, p05, p50, p95, and p99, 1st, 5th, 50th, 95th, and 99th percentiles.

**Supplemental Table 15. Weighted vs unweighted core adoption models, 2011-2015**

| **Measure** | **Term** | **Unweighted** | **Weighted** | **Delta OR** |
| --- | --- | --- | --- | --- |
| Basic - without notes | Rural vs Urban (at baseline) | 0.57 (0.50, 0.65) | 0.61 (0.53, 0.70) | 0.04 |
| Basic - without notes | Rural-by-year interaction | 0.90 (0.85, 0.96) | 0.92 (0.86, 0.99) | 0.02 |
| Basic - without notes | Year (annual change, Urban) | 2.11 (2.01, 2.21) | 2.10 (2.00, 2.21) | -0.01 |
| Basic - with notes | Rural vs Urban (at baseline) | 0.57 (0.50, 0.66) | 0.62 (0.54, 0.72) | 0.05 |
| Basic - with notes | Rural-by-year interaction | 1.01 (0.95, 1.06) | 1.00 (0.94, 1.06) | 0.00 |
| Basic - with notes | Year (annual change, Urban) | 1.93 (1.85, 2.01) | 1.96 (1.88, 2.05) | 0.04 |
| Comprehensive | Rural vs Urban (at baseline) | 0.45 (0.37, 0.55) | 0.49 (0.40, 0.59) | 0.03 |
| Comprehensive | Rural-by-year interaction | 1.05 (0.99, 1.11) | 1.04 (0.98, 1.11) | 0.00 |
| Comprehensive | Year (annual change, Urban) | 1.57 (1.51, 1.62) | 1.56 (1.50, 1.62) | 0.00 |
| Certified EHR (CEHRT) | Rural vs Urban (at baseline) | 0.74 (0.60, 0.92) | 0.80 (0.64, 1.00) | 0.05 |
| Certified EHR (CEHRT) | Rural-by-year interaction | 1.13 (0.94, 1.36) | 1.14 (0.94, 1.39) | 0.01 |
| Certified EHR (CEHRT) | Year (annual change, Urban) | 2.32 (2.05, 2.63) | 2.41 (2.12, 2.75) | 0.09 |

Odds ratios are from logistic regression models estimated with and without response weights. Δ OR is the weighted minus unweighted odds ratio. Abbreviations: CEHRT, certified EHR technology; CI, confidence interval; OR, odds ratio.

**Supplemental Table 16. Sensitivity analyses for public health reporting and structured electronic social needs capture, 2021-2023**

| **Measure** | **Year** | **All hospitals** | **Urban hospitals** | **Rural hospitals** | **Gap (Rural - Urban, percentage points)** |
| --- | --- | --- | --- | --- | --- |
| Public health: electronic case reporting (production submission) | 2021 | 60% (1,055/1,751) | 66% (674/1,016) | 52% (381/735) | -14.5 |
| Public health: electronic case reporting (production submission) | 2022 | 53% (1,189/2,240) | 59% (724/1,224) | 46% (465/1,016) | -13.4 |
| Public health: electronic case reporting sent directly from EHR | 2021 | 37% (813/2,200) | 43% (538/1,255) | 29% (275/945) | -13.8 |
| Public health: electronic case reporting sent directly from EHR | 2022 | 52% (1,245/2,382) | 59% (766/1,294) | 44% (479/1,088) | -15.2 |
| Public health: electronic case reporting sent directly from EHR | 2023 | 60% (1,449/2,408) | 71% (953/1,341) | 46% (496/1,067) | -24.6 |
| Public health: electronic case reporting production and sent from EHR | 2021 | 44% (776/1,749) | 52% (523/1,015) | 34% (253/734) | -17.1 |
| Public health: electronic case reporting production and sent from EHR | 2022 | 41% (922/2,230) | 48% (588/1,221) | 33% (334/1,009) | -15.1 |
| Public health: immunization registry reporting (production submission) | 2021 | 93% (2,025/2,176) | 97% (1,200/1,243) | 88% (825/933) | -8.1 |
| Public health: immunization registry reporting (production submission) | 2022 | 92% (2,229/2,418) | 95% (1,236/1,307) | 89% (993/1,111) | -5.2 |
| Public health: immunization registry reporting sent directly from EHR | 2021 | 74% (1,656/2,243) | 79% (1,008/1,278) | 67% (648/965) | -11.7 |
| Public health: immunization registry reporting sent directly from EHR | 2022 | 80% (1,956/2,444) | 86% (1,129/1,320) | 74% (827/1,124) | -12.0 |
| Public health: immunization registry reporting sent directly from EHR | 2023 | 80% (1,969/2,446) | 87% (1,179/1,354) | 72% (790/1,092) | -14.7 |
| Public health: immunization registry reporting production and sent from EHR | 2021 | 75% (1,612/2,155) | 80% (989/1,229) | 67% (623/926) | -13.2 |
| Public health: immunization registry reporting production and sent from EHR | 2022 | 78% (1,881/2,405) | 83% (1,082/1,299) | 72% (799/1,106) | -11.1 |
| Public health: electronic laboratory reporting (production submission) | 2021 | 87% (1,808/2,082) | 92% (1,098/1,194) | 80% (710/888) | -12.0 |
| Public health: electronic laboratory reporting (production submission) | 2022 | 90% (2,105/2,335) | 94% (1,204/1,279) | 85% (901/1,056) | -8.8 |
| Public health: electronic laboratory reporting sent directly from EHR | 2021 | 61% (1,362/2,245) | 66% (840/1,278) | 54% (522/967) | -11.7 |
| Public health: electronic laboratory reporting sent directly from EHR | 2022 | 72% (1,754/2,432) | 77% (1,013/1,316) | 66% (741/1,116) | -10.6 |
| Public health: electronic laboratory reporting sent directly from EHR | 2023 | 73% (1,768/2,421) | 79% (1,063/1,347) | 66% (705/1,074) | -13.3 |
| Public health: electronic laboratory reporting production and sent from EHR | 2021 | 61% (1,262/2,068) | 67% (799/1,186) | 52% (463/882) | -14.9 |
| Public health: electronic laboratory reporting production and sent from EHR | 2022 | 73% (1,676/2,305) | 77% (982/1,268) | 67% (694/1,037) | -10.5 |
| SDOH: structured electronic recording of social needs | 2022 | 76% (1,636/2,142) | 77% (953/1,234) | 75% (683/908) | -2.0 |
| SDOH: structured electronic recording of social needs | 2023 | 80% (1,803/2,259) | 80% (1,057/1,327) | 80% (746/932) | 0.4 |
| SDOH: screening with structured electronic recording | 2022 | 72% (1,636/2,277) | 75% (953/1,267) | 68% (683/1,010) | -7.6 |
| SDOH: screening with structured electronic recording | 2023 | 76% (1,803/2,387) | 78% (1,057/1,355) | 72% (746/1,032) | -5.7 |

Public health measures distinguish production submission and EHR-direct submission when available. Social-needs measures are restricted to structured electronic recording. Values are percentages (n/N); gaps are rural minus urban in percentage points. Abbreviations: EHR, electronic health record; ELR, electronic laboratory reporting; eCR, electronic case reporting; SDOH, social determinants of health.

**Supplemental Table 17. Annualized rural and urban growth rates in advanced EHR use measures, 2012-2023**

| **Measure** | **Years** | **Urban annualized change (percentage points per year)** | **Rural annualized change (percentage points per year)** | **Gap first year (Rural - Urban, percentage points)** | **Gap last year (Rural - Urban, percentage points)** | **Change in gap (last - first, percentage points)** |
| --- | --- | --- | --- | --- | --- | --- |
| Interoperability: Rx benefit info integrated | 2022-2023 | 6.0 | 9.0 | -10.9 | -8.3 | 2.6 |
| Interoperability: integrates received summaries | 2014-2023 | 4.6 | 4.4 | -10.0 | -11.5 | -1.5 |
| Interoperability: outside info available electronically | 2014-2023 | 4.1 | 3.7 | -13.2 | -17.0 | -3.8 |
| Interoperability: queries external records | 2012-2023 | 4.4 | 4.5 | -14.0 | -11.2 | 2.8 |
| Interoperability: uses outside info often/sometimes | 2015-2023 | 3.0 | 4.0 | -22.9 | -15.2 | 7.7 |
| Portal: FHIR-based app access | 2021-2023 | 5.0 | 6.5 | -10.9 | -7.8 | 3.1 |
| Portal: app access via API | 2021-2023 | 7.0 | 3.0 | -2.5 | -10.6 | -8.1 |
| Portal: patients can submit PGD | 2021-2023 | 3.0 | 1.5 | -1.3 | -4.9 | -3.6 |
| Portal: patients can view clinical notes | 2021-2023 | 2.5 | 4.5 | -8.7 | -3.8 | 4.9 |
| Portal: secure messaging with providers | 2021-2023 | 1.0 | 2.0 | -5.3 | -2.8 | 2.5 |
| Public health: ELR engaged | 2021-2022 | 5.0 | 3.0 | -4.3 | -6.9 | -2.6 |
| Public health: electronic case reporting engaged | 2021-2022 | 28.0 | 28.0 | -8.9 | -9.3 | -0.4 |
| Public health: immunization registry engaged | 2021-2022 | 4.0 | 2.0 | -3.1 | -5.0 | -1.9 |
| SDOH: collects social needs data | 2022-2023 | 4.0 | 4.0 | -15.3 | -14.5 | 0.8 |
| SDOH: used for analytics/population health | 2022-2023 | 7.0 | 9.0 | -6.3 | -4.5 | 1.8 |
| SDOH: used for clinical decision making | 2022-2023 | 1.0 | 0.0 | -6.8 | -7.5 | -0.7 |

Annualized change is reported in percentage points per year. Gaps are rural minus urban; positive change in gap indicates narrowing. Abbreviations: API, application programming interface; EHR, electronic health record; ELR, electronic laboratory reporting; eCR, electronic case reporting; FHIR, Fast Healthcare Interoperability Resources; PGD, patient-generated data; SDOH, social determinants of health.

**Supplemental Figure 1. Core EHR adoption over time - overall**

**
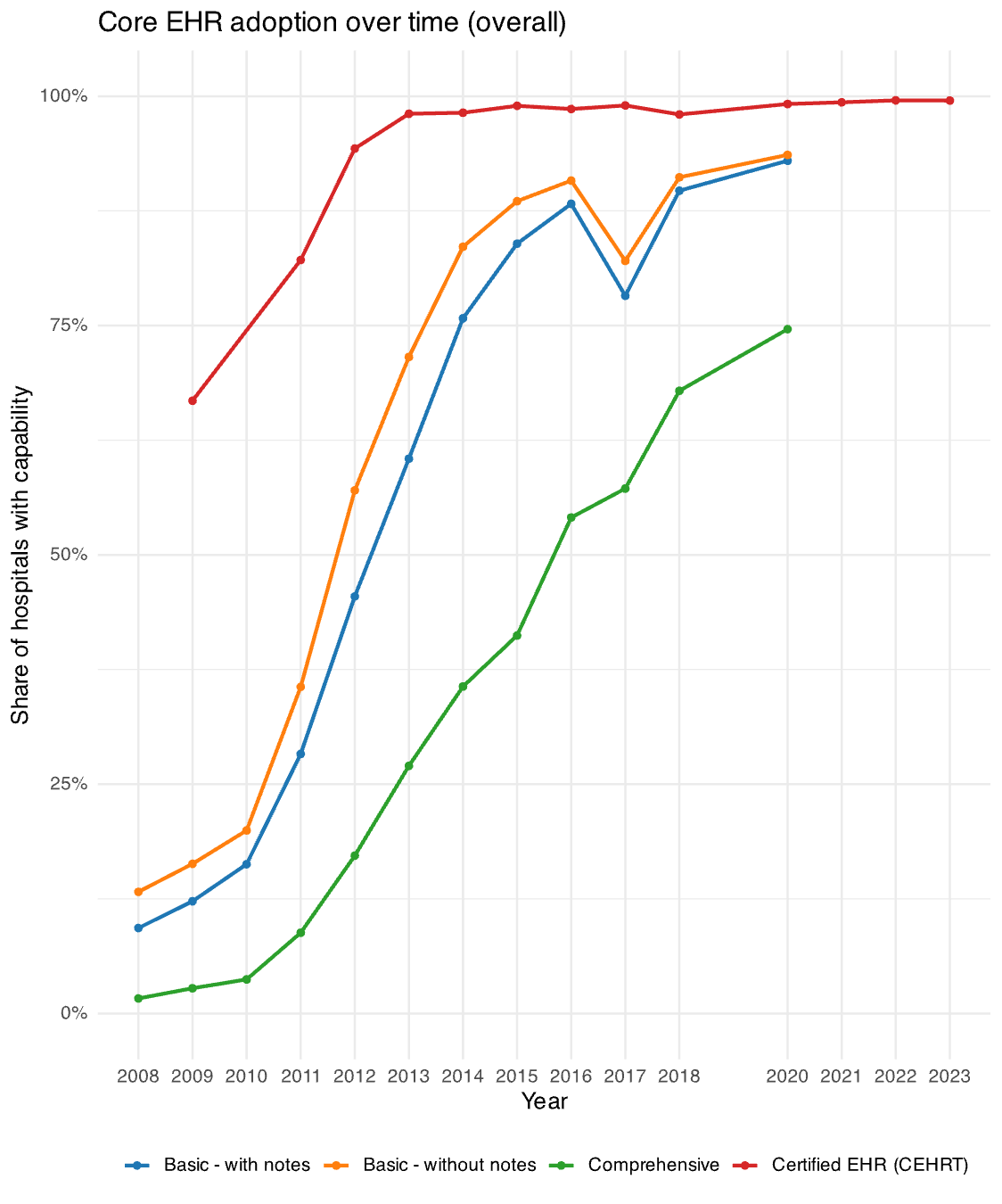
**

Overall percentages of hospitals reporting basic EHR without notes, basic EHR with notes, comprehensive EHR, and CEHRT by survey year.

**Supplemental Figure 2. Advanced EHR use over time - overall**


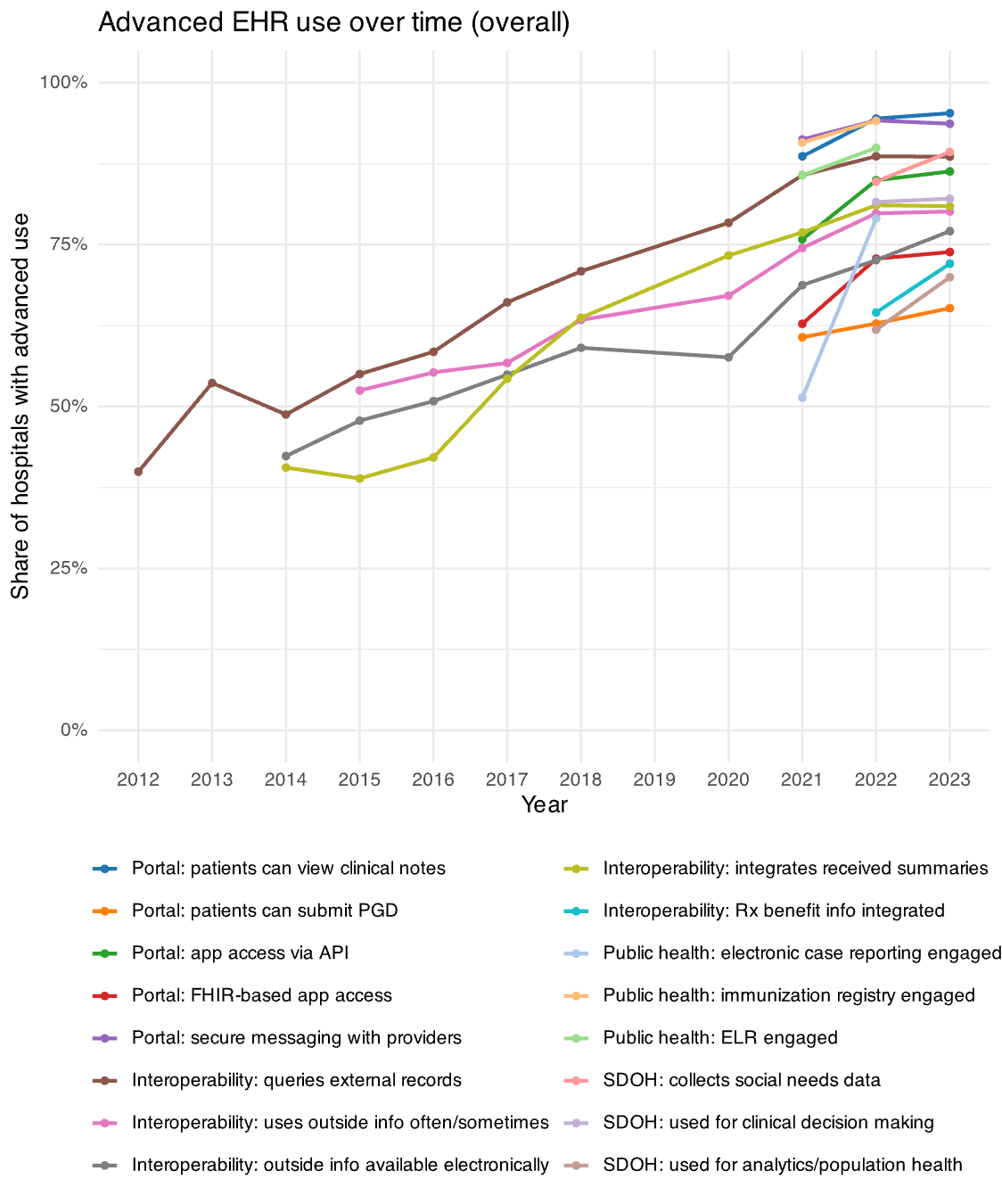


Overall percentages of hospitals reporting patient portal, interoperability, public health reporting, and social-needs measures by survey year. Measures are shown only for years each item was fielded.
